# DNA-based genealogy reconstruction of Nebbiolo, Barbera and other ancient grapevine cultivars from northwestern Italy

**DOI:** 10.1038/s41598-020-72799-6

**Published:** 2020-09-25

**Authors:** Stefano Raimondi, Giorgio Tumino, Paola Ruffa, Paolo Boccacci, Giorgio Gambino, Anna Schneider

**Affiliations:** 1 National Research Council of Italy-Institute for Sustainable Plant Protection (CNR-IPSP), Strada delle Cacce 73, 10135 Turin, Italy; 2 Council for Agricultural Research and Economics-Research Centre for Genomics and Bioinformatics, Via San Protaso 302, 29017 Fiorenzuola d’Arda, PC Italy; 3 University of Turin-Department of Agricultural, Forest and Food Sciences (UNITO-DiSAFA), L. Braccini 2, 10095 Grugliasco, Turin Italy

**Keywords:** Genetics, Molecular biology, Plant sciences

## Abstract

Northwestern Italy is a wine region of the world with the highest of reputations, where top quality wines of remarkable economic value are produced from traditional, long-cultivated varieties. Kinship analyses were performed using 32 microsatellite loci and more than 10 K single-nucleotide polymorphism markers on 227 traditional grapes mostly from Northwestern Italy—including those that have been neglected or are threatened. This was done to better understand the genetic grapevine origins and history of this reputable wine producing area, thus enhancing its cultural value and the marketing appeal of its wines. The work revealed a complex network of genetic relationships among varieties, with little contribution of genotypes from other areas. It revealed the major role played by a few ancient grape varieties as parents of numerous offspring, including some that are endangered today. The ancestry of many cultivars is proposed. Among these are Dolcetto, Barbera and Riesling italico. Through the inference of parent–offspring and sibling relations, marker profiles of ungenotyped putative parents were reconstructed, suggesting kinship relations and a possible parentage for Nebbiolo, one of the most ancient wine grapes worldwide. Historic and geographic implications from the resulting kinships are discussed.

## Introduction

Grapevines are one of the most economically valuable agricultural crops in the world, accounting for a total area of 7,450,000 ha and a grape production of 75,500,000 t (O.I.V, data 2016, www.oiv.int/en/statistiques). Current production is based on cultivars selected or created for fresh consumption, and on a wide range of varieties for wine production, both vegetatively propagated. The natural diversity in this species was most likely the key to its great spread over time throughout the Mediterranean and Continental Europe, where modern wine industry expanded, further broadening from the Old to the New World^1^.

Within a few millennia, thousands of domesticated varieties had developed in Europe and western Asia, then migrated or disappeared. In so doing they shaped the local varietal assortment in every established wine region. Up to now, wine production has rested mostly on centuries-old *Vitis vinifera* varieties, as expressed in its traditional and historical use. Despite some successful achievements through modern breeding, traditional ancient cultivars still represent by far most of the wine industry’s base, especially for wines of high renown and high price.

Some of the finest grape varieties have been well documented since the Middle Ages. According to Viala and Vermorel^2^ the cultivar Pinot was first mentioned in the second half of XIV century, while historical references to Garganega and Nebbiolo go back to early 1300 and the second half of 1200, respectively. They are among the first grapes to be described in the written word in Italy, although it should be noted that the absence of historical documentation does not necessarily imply that any specific variety did not exist prior to these references.

Both the geographic and genetic origins of ancient *vinifera* cultivated grapes, as well as more generally the transition process from wild to domesticated forms, have been debated for a very long time. In the early 1990s, the creation from sexual reproduction by natural hybridization between two older genitors was demonstrated for the first time in traditional cultivars using the development of grape DNA typing by molecular markers^3,4^. This proved what many scholars had long presumed. Parentage studies provide evidence of: (1) the likely geographic origin of ancient grape varieties^5^, (2) their descendants and relatives^6–10^, and (3) their history and migrations over time. Forgotten grapes, currently of little importance, were discovered and in some cases provided missing links that reveal the history of more relevant progenies^11–13^.

Factors and events that contribute to the high variation among today’s cultivars (and wines) have long been a matter of speculation, not only in regard to the economic relevance of grapevine as a crop, but also in regard to their indubitable cultural heritage. This speculation has not only contributed to scientific advancement in grapevine genetics, but also to a better understanding about the origin of cultivated grapes, complementing the high interest on variety history for wine marketing.

When applied on a large scale, genotyping offers information about cultivated grapevine genetic clusters^14–18^, and relation between currently cultivated varieties and wild *Vitis vinifera* subsp. *sylvestris* genetic pool^19–23^. In turn, this provides insight into domestication processes and helps us trace the evolution and dissemination of viticulture and wine culture.

Until the development of Single Nucleotide Polymorphisms (SNPs) for grapevine typing^24^, Simple Sequence Repeats (SSRs) or microsatellites have been favoured molecular markers for varietal identification along with parentage analyses for several decades^25–28^. Due to their stability, high reproducibility and polymorphism combined with co-dominant Mendelian inheritance, nuclear-SSRs (nSSRs) were widely applied to grapevines. They were also used for building reference databases (*Vitis* International Variety Catalogue, https://www.vivc.de; European *Vitis* Database, https://www.eu-vitis.de; Italian *Vitis* Database, https://vitisdb.it/).

More recently, several SNP sets have been developed for *Vitis* spp.^29–31^. Their use is increasing, taking advantage of automated processing, high repeatability, and the richness of information they provide, depending on the number of SNPs scored^14,16,22,32,33^.

The analysis of chloroplast DNA has been used in phylogenetic studies^34^. Grapevine chloroplasts are maternally inherited. Chlorotype identification within *V. vinifera* germplasm has allowed the definition of the maternal lineage of cultivars, along with the investigation on their relations to the *sylvestris* subspecies and their geographic distribution^20,35^. Therefore, despite the low genetic diversity, grape chlorotypes can give insight to the origin of ancient cultivars and their dissemination patterns^26^.

Among grape growing countries, Italy is one of the richest in *vinifera* genetic diversity, both in wild and cultivated forms. Eco-geographic factors (central Mediterranean location, high ecological fragmentation, mild climate, and significant coast development dotted with sheltered ports suitable for mariners’ landing), together with human history (migrations, colonization and dominations) have, in all likelihood, contributed to the large number of grapevine cultivars found on the Italian peninsula.

Italy is the third largest wine grape growing country after Spain and France, with 710,000 ha under vines (O.I.V., data 2016, https://www.oiv.int/en/statistiques/?year=2016&countryCode=ITA). However, according to the *Vitis* International Variety Catalogue (VIVC) (https://www.vivc.de, March 2020^36^) the number of traditional *vinifera* wine varieties designated to be of Italian origin are 1,038, compared to 348 and 552 for Spain and France, respectively. The Italian varietal assortment for wine production is wide and complex, with more than 500 wines under Protected Denominations of Origin (PDO) and Protected Geographical Indications (PGI) currently recognized. This amounts to a third of European appellation wines.

Piedmont (northwestern Italy) is one of the most renowned wine areas of the world where premium wines from the centuries-old cultivar Nebbiolo such as Barolo, Barbaresco, Ghemme and Gattinara are produced. The region is also the likely home of Barbera and Dolcetto, respectively the major and the third regional black grapes that contribute to fine PDO wines. Moreover, Piedmont harbors White muscat (Moscato bianco, alias Muscat à petits grains blancs), a fragrant grape that goes into the popular sparkling wine Asti, as well as several other aromatic varieties. There are 47 local varieties permitted for cultivation on the current 45,000 ha under vine of the region. They all arise from natural hybridization and selection rather than breeding. Twenty other wine grapes grown in the region have been introduced from abroad. Nearly 200, however, are the grape varieties of presently very scarce cultural importance, neglected, or even severely threatened of extinction. They have been recovered and collected over the last four decades, mostly in ancient vineyards located in today’s marginal wine areas and are all gathered in a central regional ex situ collection located in Grinzane Cavour (south western Piedmont). This ancient, local grape germplasm is a potential key to a reconstructed genetic history of today’s most popular cultivars from Piedmont.

With this in mind we have focused on traditional grape varieties cultivated today or in the past in northwestern Italy. Our research had three main objectives: (1) to assess the correct identification of traditional varieties, using DNA fingerprinting, together with ampelographic resources and historical reports; (2) to investigate the genealogy and geographic origin of traditional grapes (taking into account potential migration from other areas), with the final aim of (3) revealing local wine history and culture.

The work relies on the inheritance of both nuclear and chloroplast SSR markers along with SNPs. An original approach was applied for inferring un-genotyped individuals and this played a key role in the resulting reconstructed pedigree.

## Results

### Varietal identification

**Figure 1:**
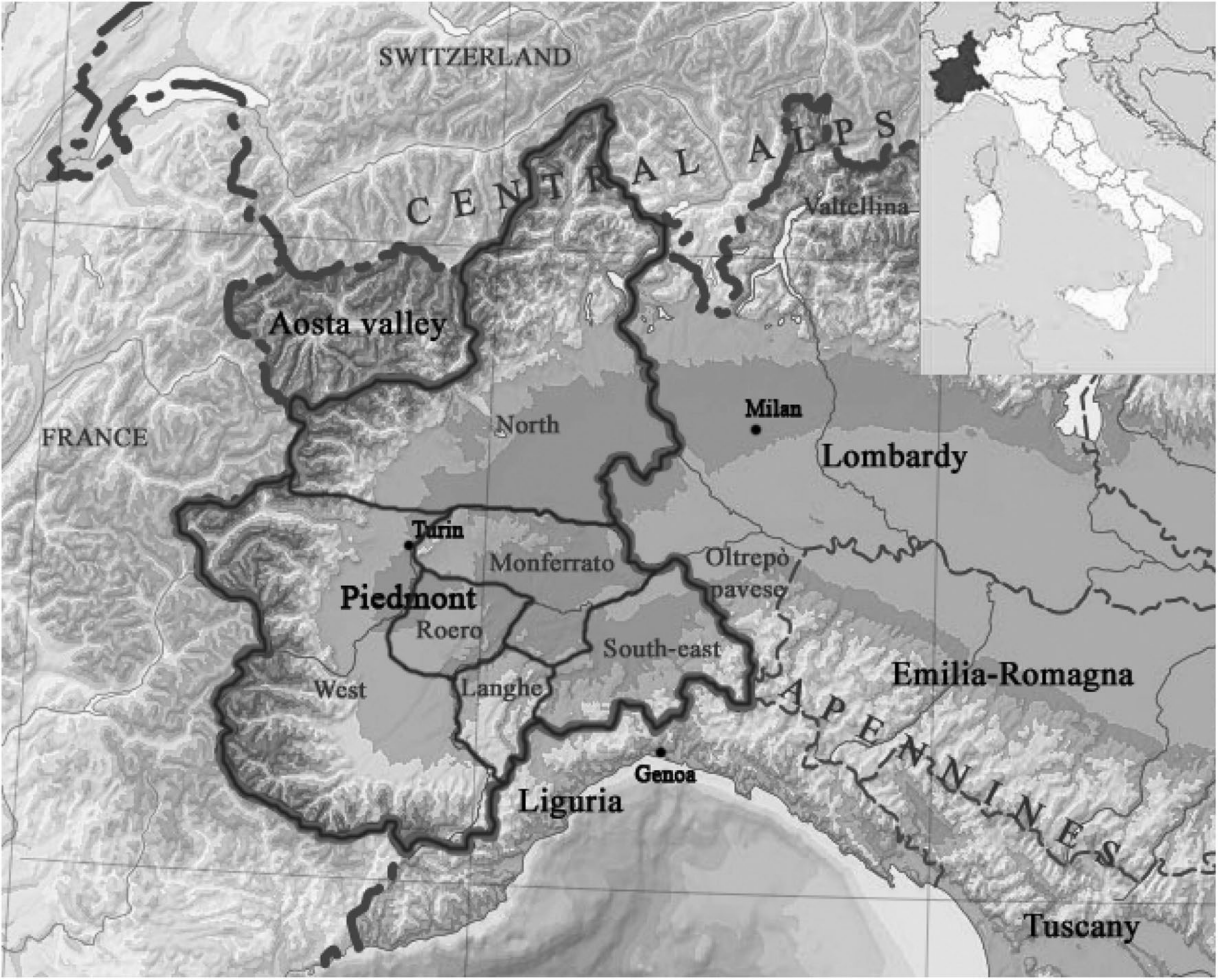
The region of Piedmont in north western Italy and its major wine districts. Image modified from Blank topographic map from Wikimedia Commons. © Eric Gaba for Wikimedia Commons. Licensed under CC BY-SA 3.0 license (https://commons.wikimedia.org/wiki/File:Italy_topographic_map-blank.svg).

All the 186 unique genotypes from north-western Italy included in this study (Supplementary Table S1; Fig. 1) were first examined for their varietal identity. Sixty genotypes corresponded to varieties registered in the Italian National Grapevine Variety Catalogue (https://catalogoviti.politicheagricole.it/catalogo.php), as indicated in Supplementary Table S1.

Most of the varieties bearing local names were identified and designated according to historical and ampelographic evidence. Vines belonging to a single variety but lacking a name were identified by comparing vine features with reliable historical descriptions and/or plates. The un-named varieties identified by this process (10 out of 22) were specified as “presumed”. For the remaining 12 genotypes no name could be found or assigned. Therefore, they were indicated as “unknown white” or “unknown black” according to their berry colour.

The accession Coccalona nera, for example, a key grape in our work, was recovered in southeastern Piedmont as an un-named variety. Some key traits found in historical descriptions of Coccalona nera^37^ led us to identify this unknown accession as the historical Coccalona from Piedmont. This conclusion arose from several factors, including the extraordinary abundance of leaf hairiness, the attribute to over-crop when grown in deep soils, many morphological features in common with varieties resulting from our data genetically related, and similar places of current recovery and growth in the past. Through SSRs comparison, we discovered this variety was identical to Orsolina from Reggio Emilia province (described by Bignami et al.^38^) and to Rohrtraube blaurot included in VIVC (https://www.vivc.de/index.php?r=passport%2Fview&id=10162). It was reported in the first half of the 19th century by Babo and Metzger^39^ as an ancient variety from south western Germany.

The specification “false” was used to indicate a mislabeled accession. For example, Aleatico (from Paderna) is wrongly named, as the true Aleatico is a different cultivar mainly grown in central and southern Italy. Hence, Aleatico (from Paderna) was designated as “false”. Bottagera, another key grape in our work, is probably in-correctly labeled as well. Unlike the accession we collected under this name, the true historical Bottagera is reported to exhibit large berries and poor quality^40^.

### Population genetic parameters and chlorotype frequency

The sample consisted of: 196 traditional grape varieties from northwestern Italy and from other European regions linked by parent/offspring relationships, 12 cultivars used as references for the pedigree study, along with 19 outgroup *V. vinifera* genotypes.

Population genetics parameters (Supplementary Table S2) obtained from the nSSR allelic frequencies (32 loci) (Supplementary Table S3) indicated the individuals were in Hardy–Weinberg equilibrium. The observed heterozygosity^41^ was slightly higher than the expected one for most of the loci. Polymorphic Information Content^42^ was very high, ranging from 17 to 5, as expected especially for the markers with a high number of alleles. The average non-exclusion probabilities of the cumulative nSSR marker set were very high, strengthening parentage analysis outcomes. As for the estimated null allele frequency, it is worth noting the poor performance of VvMD36, with a value slightly higher than the threshold of 0.5 generally suggested for locus exclusion.

Chlorotypes were defined using either four cpSSR or four plastidial SNPs, independently (for SNP outputs see Supplementary Table S4). For 97 samples both marker types were used (Supplementary Table S1), providing identical chlorotypes. The four main chlorotypes usually described in *V. vinifera* were found with very different frequencies (Table 1): 15% of varieties (13% for cultivars from Piedmont) carried chlorotype A; chlorotype B was found in Zibibbo (alias Muscat of Alexandria) only; chlorotype C occurred in a very limited number of grapes, such as Gouais blanc and most of its progeny; chlorotype D was the most frequent of chlorotypes, either in the whole sample set (81%) or in Piedmont varieties (85%).

**Table 1:** Chlorotype frequencies observed in a set of 235 cultivars.

Chlorotype	Cultivars from Piedmont		All analyzed cultivars	
	Number	%	Number	%
A	21	13.0	36	15.3
B	0	0.0	1	0.4
C	3	1.9	8	3.4
D	138	85.2	190	80.9
Total	162	100.0	235	100.0

Chlorotype labels are after Arroyo-García et al.^20, 80^^.^

### Direct kinship relationships

**Figure 2:**
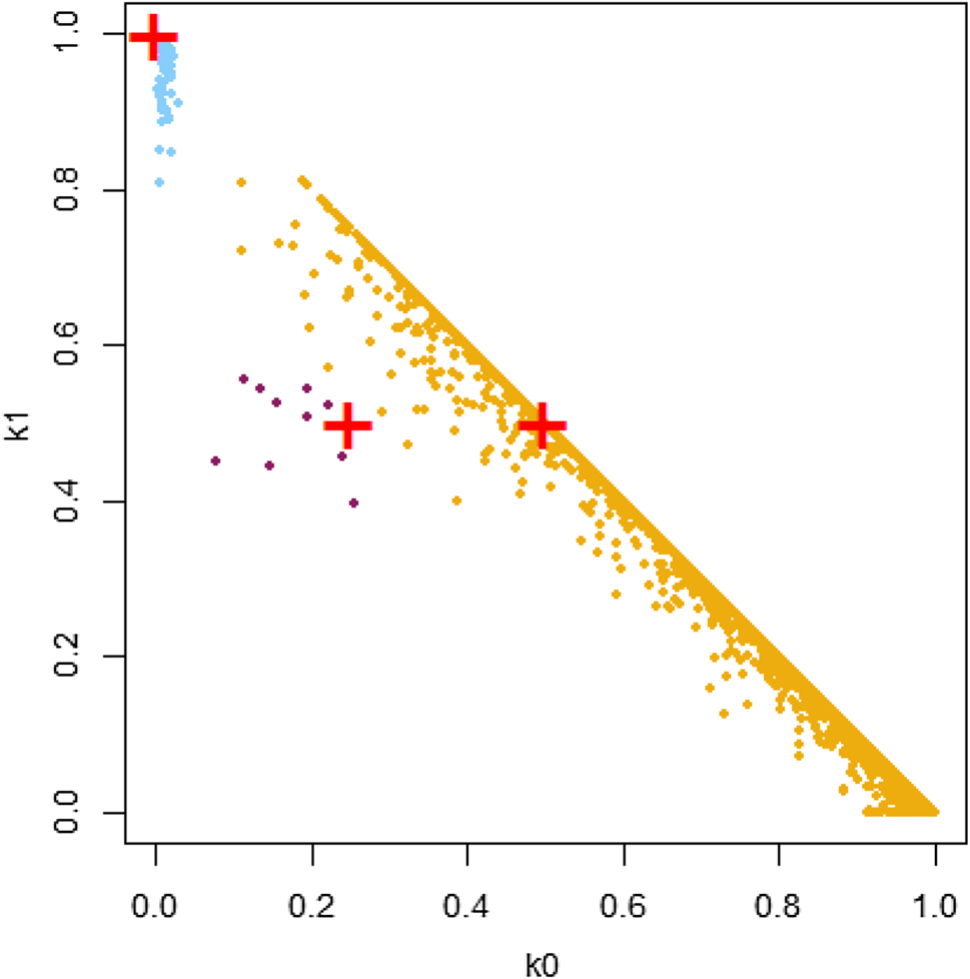
Pairwise IBD coefficients (k0 versus k1) estimated by the PLINK method of moments. Plus symbols indicate theoretical values for PO (k0 = 0, k1 = 1), FS (k0 = 0.25, k1 = 0.5) and HS (k0 = 0.5, k1 = 0.5) relationships. Points are coloured according to the assigned type of relationship: PO (blue), FS (brown), HS and higher degrees of relationship (orange). The discrimination between FS and HS was based on an arbitrary-chosen conservative threshold of k2 = 0.25.

A total of 176 parent/offspring (PO) kinship pairs involving 169 grapevine varieties were detected by using 32 nSSRs and about 10 K SNPs (Supplementary Table S5). LOD scores ranged from 7,02E+14 to 3,41E+15 for nSSR, with a high proportion of matching loci. SNP-derived IBD coefficients clearly discriminated PO relations, based on k0 values lower than 0.03 and k1 values greater than 0.8 (Fig. 2).

One hundred and twelve (112) of the PO related cultivars were grown or collected in Piedmont (for information on growing or collecting region see Supplementary Table S1), while 57 were typical of other geographic areas but nevertheless linked to varieties from Piedmont. Consequently, most of the related kin groups involved genotypes from the same region. In other words, some traditional wine growing regions like Piedmont developed a variety assortment unique and strongly interconnected, thus entailing typical, highly representative local wines.

A group of grape varieties belonging to the original sample set from Piedmont did not display any PO relationships. However, some of them (e.g. Arneis, Grignolino and Erbaluce) were still related at a higher degree. Quagliano, Pelaverga piccolo (the variety behind Verduno wine), Verdea and Favorita (alias Vermentino from Liguria and Sardinia) are examples of unrelated varieties.

PO relationships were consistently detected by nSSR and SNPs, except for few cases. For the few pairs showing 1 or 2 SSR mismatches (out of the 32 loci), IBD coefficients and SNP Mendelian errors strongly supported parent/offspring relationships. Fifty-six percent of total microsatellite discrepancies were observed at locus VvMD36 (Supplementary Table S5). nSSR failed to reveal the two historical varieties Albana and Garganega as PO related, since discrepancies were detected on 3 of 32 loci. This could be caused by slightly divergent profiles arising from the accumulation of somatic mutations following the numberless propagation cycles in very ancient seedlings—both Albana and Garganega go back to XIII century. Conversely, the PO relationship detected by nSSR between Neyret and Petit rouge was not confirmed by SNP markers. Moreover, SNPs brought into question the parent/offspring kinship between Nebbiolo and Nebbiolo rosé, suggesting a full-sibling relationship might be possible.

Forty-four trios (each made up by the two parents and the offspring) were detected by Cervus using SSR data (Table 2). Most pedigrees were corroborated by Colony, except for 5 trios resulting inconsistent with the “best configuration” proposed by this software. Twenty-one families, genotyped with SNPs, were confirmed by negligible Mendelian errors (maximum 23 errors, Table 2) while the minimum number of errors from the distribution of putatively false trios was 283.

**Table 2:** The 44 resulting pedigrees (putative offspring/parent 1/parent 2) and related statistics based on nSSR (32 loci) and SNPs (around 10 K).

Offspring	Parent 1	Parent 2	SSR			SNPs		Confirmation of prior publication
			Cervus		Confirmed by Colony	Mendelian errors	Mendelian errors (%)	
			Matching loci	LOD score				
Avarenchetto	Bottagera (false) ♂	Gouais blanc (Liseiret B.) ♀	32/32	4.48E+15	Yes			
Barbera bianca B.	Crovin (from Perti)	Timorasso B.	32/32	5.63E+15	Yes	12	0.26	
Barbera di Patrunat	Moscato bianco B.	Neretto duro (Balau)	31/32	3.83E+15	No			
Berla grossa	Gouais blanc (Liseiret B.) ♀	Lambrusca vittona ♂	32/32	4.98E+15	Yes			
Bianchetta (from Frascata)	Dolcetto bianco	Patlassa (long internodes, presumed)	32/32	4.82E+15	Yes			
Biestro (presumed)	Dolcetto bianco	Moissan	31/32	3.93E+15	No			
Bonardina	Croatina N. ♂	Malvasia aromatica di Parma ♀	31/32	4.17E+15	Yes	23	0.47	
Brachetto Migliardi	Lambrusca di Alessandria N. ♂	Malvasia aromatica di Parma ♀	32/32	3.78E+15	Yes	12	0.25	^43^
Bubbierasco	Bianchetto (from Saluzzo)	Nebbiolo N.	32/32	5.17E+15	Yes	9	0.19	^81^
**Cabernet sauvignon N.**	**Cabernet franc N.**	**Sauvignon B.**	32/32	7.30E+15	Yes			^4^
**Chardonnay B.**	**Gouais blanc (Liseiret B.)** ♀	**Pinot nero N.** ♂	32/32	6.02E+15	Yes	20	0.43	^5^
**Charmont B**	**Chardonnay B.**	**Chasselas blanc B**	32/32	4.71E+15	Yes	16	0.33	^82^
Dolcetto N.	Dolcetto bianco	Moissan	32/32	4.68E+15	Yes	10	0.2	^44^
Durasa N.	Coccalona nera (presumed) ♂	Marzemino N. ♀	31/32	4.36E+15	Yes	11	0.24	
Feral accession	Dolcetto N. ♀	Riesling italico B. ♂	32/32	5.49E+15	Yes			
**Gamay N.**	**Gouais blanc (Liseiret B.)** ♀	**Pinot nero N.** ♂	32/32	5.40E+15	Yes	15	0.33	^5^
Gris	Avarengo N.	Moissan	32/32	4.62E+15	Yes			
Grisa (from Cumiana)	Avarengo N. ♀	Bottagera (false) ♂	30/32	3.26E+15	Yes	16	0.32	
Lacrima Cristi	Avarengo N. ♂	Doux d'Henry N. ♀	31/32	3.29E+15	Yes			
Lambrusca di Alessandria N.	Crovin (from Perti)	Neretto di Marengo	32/32	5.59E+15	Yes	12	0.25	
Lambrusca pignata	Chatus N. ♂	Doux d'Henry N. ♀	32/32	4.28E+15	Yes			
Luglienga moscata	Moscato bianco B.	Luglienga bianca	32/32	5.58E+15	Yes			^43^
Malvasia bianca (from Vignale)	Coccalona bianca ♂	Malvasia aromatica di Parma ♀	32/32	4.62E+15	Yes			^43^
Malvasia di Casorzo N.	Lambrusca di Alessandria N. ♂	Malvasia aromatica di Parma ♀	32/32	5.59E+15	Yes	12	0.25	^43^
Malvasia nera (from Costa V.)	Lambrusca di Alessandria N. ♂	Malvasia aromatica di Parma ♀	32/32	4.69E+15	Yes			
Malvasia nera lunga N.	Freisa N. ♂	Malvasia aromatica di Parma ♀	31/32	4.03E+15	Yes	9	0.18	^43^
Moscato bianco precoce	Dolcetto bianco	Moscato bianco B.	32/32	4.83E+15	Yes	23	0.43	
Moscato nero (Borbera valley)	Coccalona nera (presumed) ♂	Moscato bianco B. ♀	31/32	4.11E+15	Yes			
Moscato nero di Acqui N.	Coccalona nera (presumed) ♂	Moscato bianco B. ♀	32/32	4.87E+15	Yes	15	0.31	
Mossano (from Canavese area)	Bottagera (false) ♂	Moissan ♀	32/32	4.23E+15	Yes			
**Muscat rouge de Madère**	**Moscato bianco B.**	**Sciaccarello N**	32/32	4.44E+15	Yes			^63^
Nebbiolo di Dronero (false)	Chatus N. ♂	Doux d'Henry N. ♀	30/32	3.85E+15	Yes	12	0.26	
Nebbiolo gabardin	Chatus N.	Neretta cuneese N.	32/32	5.26E+15	No			
Nebùe	Malvasia aromatica di Parma ♀	Neretto di Marengo ♂	32/32	4.52E+15	Yes			^43^
**Orange muscat**	**Chasselas blanc B**	**Moscato bianco B.**	32/32	3.90E+15	Yes			^83^
Primaticcia	Croatina N.	Lambrusca di Alessandria N.	31/32	5.13E+15	Yes			
Ruché N.	Croatina N.	Malvasia aromatica di Parma ♀	32/32	5.02E+15	Yes	10	0.21	^43^
Tadone 1 (from Saluzzo)	Grec rouge ♂	Tadone 2 (from Saluzzo) ♀	32/32	4.80E+15	Yes	10	0.21	
Teinturier ad acino rotondo	Neretto duro (Balau) ♂	Teinturier du Cher ♀	31/32	4.58E+15	No	7	0.13	
Unknown black (from Fresonara)	Barbera N. ♂	Cortese B. ♀	31/32	5.50E+15	No			
Unknown black (from Pomaretto)	Chiavennasca bianca	Moissan	31/32	3.51E+15	Yes	8	0.18	
Uva delle cascine	Bianchetta (from Frascata)	Moradella N.	32/32	4.73E+15	Yes			
Vespolina N.	Coccalona nera (presumed) ♂	Nebbiolo N. ♀	31/32	3.82E+15	Yes	14	0.32	
**Zibibbo B. (M. of Alexandria)**	**Heptakilo**	**Moscato bianco B.**	32/32	6.40E+15	Yes			^28^

Whenever possible, the male or female parental contribution is indicated. Known pedigrees used as references are in bold. Pedigrees non confirmed by Colony are inconsistent with its suggested "Best Configuration".

Some resulting parentages refer to well-known pedigrees, already published and repeatedly confirmed, like Cabernet Sauvignon, Chardonnay, Muscat rouge de Madère. They were used as reference for LOD scores and IBD coefficient evaluation. Others refer to traditional varieties from Piedmont already investigated (among which Malvasia di Casorzo, Ruchè), often sharing as genitor Malvasia aromatica di Parma^43^. As to the group of flavored grape varieties descending from Moscato bianco, the study revealed the second parent of Moscato nero di Acqui, a fragrant specialty from south east of the region.

Our results did not confirm some pedigrees already proposed. Freisa was believed descended from Nebbiolo and Avanà (Hibou noir in France)^27^. Our findings support Nebbiolo as true parent, definitely excluding Avanà as Freisa’s second genitor.

Most of the proposed trios (28 out of 44) are new insights, involving highly reputed and widely cultivated varieties. Dolcetto, whose pedigree was documented in a preliminary work^44^, was confirmed here. It arose from two ancient grapes no longer cultivated, now surviving in the regional repository. Lambrusca di Alessandria, a robust dark-coloured variety used for blending, originated from today’s minor or even neglected genitors. Nebbiolo’s descendant Vespolina is an original specialty used in northern Piedmont for varietal wines or in blends with Nebbiolo. Barbera bianca (White Barbera)—not the white somatic variant of Barbera but a distinct seedling—did not reveal any genetic proximity to Barbera. The findings confirmed the parentage of Teinturier ad acino rotondo (Round berry Teinturier), a red-fleshed variety distributed across northern Italy and mentioned by Crespan et al.^45^ as Tintoria Lloyd. This suggests Piedmont is its cradle due to: (1) its closeness to France where its genitor Teinturier du Cher has been widely spread in the past, and (2) the scattered presence in Piedmont of its second parent Neretto duro (syn. Balau), quoted here since XVII century.

In 14 of the proposed trios one of the two parents displayed a chlorotype different from the other, allowing us to define motherhood and fatherhood (Table 2). Motherhood was clear for Malvasia aromatica di Parma and Doux d’Henry, both affected by male sterility.

### Full-sibling relations

**Figure 3:**
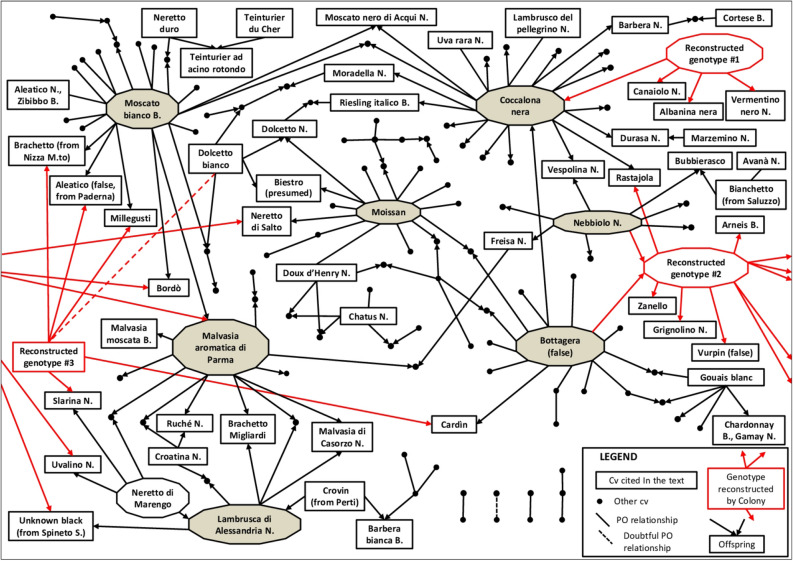
Kinship network of the traditional grape varieties from north western Italy and their close relatives. Only main parents and varieties mentioned along the text are labeled. Black connector lines indicate parent/offspring relationships; the arrows point to the offspring. Red connectors and red arrows refer to reconstructed genotypes (not included into the sample set). Dashed lines represent doubtful PO relationships.

Evidence of full-sibling (FS) relationships among the investigated varieties was obtained by nSSRs and SNPs (Supplementary Table S6 for FS; Figs. 2, 3).

The total number of indirect relationships (i.e. half-sibling, grandparent-grandchild, avuncular) were around 930 for the whole sample set when inferred by SSR data (data not shown), while more than 20 pairs were linked by full-sibling kinship (Supplementary Table S6). The probability of a full-sibling link provided by Colony is based on nSSR data, while for 18 pairs SNP-based IBD coefficients were presented as well (expected IBD values for FS relations are 0.25, 0.50, 0.25 for k0, k1 and k2, respectively). IBD coefficients were unable to clearly distinguish full-from half-sibling (HS) (Fig. 2), although using a conservative threshold of k2 = 0.25 it was possible to highlight relations that are likely FS (Fig. 2, brown points). The classification of relations with k2 values just below 0.25 was considered uncertain.

The pair Chardonnay–Gamay, used as reference, was correctly confirmed as full-brothers, whilst an uncertain interpretation remains for the debated FS relationship between Syrah and Viognier. The full-brother relationship between Biestro (presumed) and Dolcetto, both offspring of Dolcetto bianco and Moissan according to pedigree outcomes by Cervus (Table 2), was inconsistent with the “best configuration” proposed by Colony for this pedigree reconstruction (Supplementary Table S6). Furthermore, SNP relatedness coefficients did not support the full sibling relationships for Fumin–Neyret and Fumin–Petit rouge (the latter being asserted by Vouillamoz and Moriondo^46^). They suggest instead a half-brothers relationship for both pairs, as in the one between Neyret and Petit rouge. As to Nebbiolo’s kingroup, relatedness analyses from SNP and SSR profiles clearly matched, indicating Nebbiolo, Nebbiolo rosé, Pignola and Rossola nera share both parents.

### The reconstructed pedigree network

A kin network of grapevine cultivars mainly from north western Italy was inferred using the likelihood-based algorithm implemented in Colony (Figs. 3; Supplementary S1). The outcomes were based on SSR data, while the SNP data set was used for a subset of individuals to confirm the most relevant HS clusters. Five main genitors clearly emerge from the overall network, accounting for 11–21 parent/offspring relations each: Moscato bianco and Malvasia aromatica di Parma, already known as founders of most of the aromatic grapes from Piedmont^43^, Moissan, Coccalona nera and Bottagera (false). Two further varieties, Lambrusca di Alessandria and Nebbiolo, have also an important genetic role as parents, showing 5–8 presumed descendants (Table 3). Except for Moscato bianco and Nebbiolo, the other founders are grapes of minor commercial importance (like Lambrusca di Alessandria), neglected if not endangered, such as Malvasia aromatica di Parma, Moissan, Coccalona nera and Bottagera (false). These were recovered from single plants often in only one location.

**Table 3:** Main genitors of grape traditional cultivars from north western Italy.

Cultivar name	N. of offspring	N. of further PO relationships	Likely pedigree	First historic mention	Main synonyms (synonym’s geographic locations)
Moscato bianco	10	11	Unknown	Unknown (1303 in Piedmont)	Muscat à petits grains blancs (France), etc. (other 50 synonyms reported in VIVC)
Malvasia aromatica di Parma	11	–	Moscato bianco’s offspring	1798	Malvasia Casalini (Italy), Muscadelle du Bordelais (faux) (France, but introduced from abroad)
Moissan	6	8	Unknown	1606	Morcian (Liguria), Mauzanetto (Piedmont, Italy, where it was cited the first time)
Coccalona nera	20	–	Bottagera (false)’s offspring	1800	Orsolina (Emilia-Romagna, Italy), Rohrtraube blaurot, Rother Hudler (Germany)
Bottagera (false)	6	8	Unknown	Unknown	Unknown
Lambrusca di Alessandria	5	–	Neretto di Marengo x Crovin (from Perti)	1798	Moretto, Pezzé, Croetto (Italy)
Nebbiolo	8	–	Unknown	1266	Spanna, Chiavennasca, Picoltener, Prunent (Italy)

The number of further PO relationships in the third column does not include the number of offspring.

The reconstruction of genotypes #2 and #3 (indicated by red connectors and arrows in Figs. 3 and supplementary S1) permitted adding hypothetical key missing links into the parentage network. Genotype #2 could be involved in as many as 6 trios, giving rise with 5 different partners to Rastajola and (in the left side of Figs. 3, Supplementary Fig. S1) to Neretto di Salto, Uvalino, Unknown black (from Spineto), Bordò and Malvasia aromatica di Parma (Fig. 3). The latter two putative offspring would share both parents, in line with SNP IBD coefficients expected for full-siblings (Supplementary Table S6). Genotype #2 could also be PO related to Arneis, Vurpin (false), Grignolino, Zanello, Bottagera and Nebbiolo. The genotype #2 cluster was identically inferred by using SNP data. Moreover, the reconstructed nSSR profile of genotype #2 (Supplementary Table S7) matched at 32 SSR loci as offspring of Bottagera and Nebbiolo. Thus, the other suggested PO relationships (Arneis, Grignolino, etc.) would turn out to be genotype #2’s offspring (hence Nebbiolo’s and Bottagera’s grandchildren). These findings were confirmed by SNP data, counting Mendelian errors in all the possible pedigrees that involved an offspring, a parent and two grandparents for the other lineage (Table 4). However, the relationship between Arneis and Bottagera remains uncertain, since a FS relation was suggested by Colony, that could be compatible with SNP IBD values (Supplementary Table S6).

**Table 4:** Mendelian errors (by SNPs) supporting kinships via grandparents, when one parent is missing.

Parent_1	Grandparent_1	Grandparent_2	Child	Mendelian errors	# loci compared	Mendelian errors (%)
Moscato bianco	Nebbiolo	Bottagera (false)	Malvasia aromatica di Parma	1	2,869	0.03
Coccalona nera (presumed)	Nebbiolo	Bottagera (false)	Rastajola	2	3,136	0.06
Moissan	Nebbiolo	Bottagera (false)	Neretto di Salto	2	2,733	0.07
Moscato bianco	Nebbiolo	Bottagera (false)	Bordò	2	2,870	0.07
Lambrusca di Alessandria	Nebbiolo	Bottagera (false)	Unknown black (from Spineto)	2	2,733	0.07
Moscato bianco	Avarengo	Bottagera (false)	Brachetto	3	3,003	0.10
Neretto di Marengo	Nebbiolo	Bottagera (false)	Uvalino	4	2,875	0.14

Reconstructed genotype #3 finds a place in the parentages of several minor aromatic varieties in partnership with Moscato bianco: Millegusti, Brachetto (from Nizza M.to) and Aleatico (false, from Paderna). These are, in fact, compatible with a full-siblings relationship (Supplementary Table S6). Genotype #3 is also involved in the origin of Cardìn and Slarina, the latter an historical, presently revived variety for fine wines.

### Nebbiolo’s kingroup reconstruction

Nebbiolo is one of the earliest European grapes, first mentioned in 1266 near Turin^47^. Nebbiolo’s kingroup comprises 7 varieties linked by PO relationships to Nebbiolo itself (Figs. 3, Supplementary S1). However, for none of these grapes is there a high probability of being a Nebbiolo’s parent, rather the progeny. The second genitor for Vespolina and Bubbierasco is established, for the others it is still missing. Both Nebbiolo’s parents are probably lost in time. This is plausible given the very old age of this grape.

**Figure 4:**
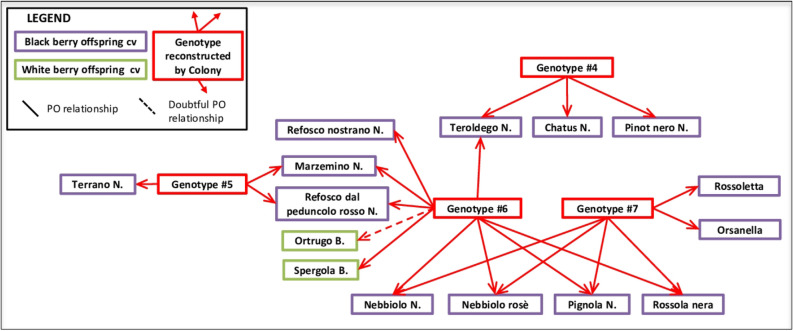
Pedigree of Nebbiolo and related grape varieties. White and coloured grape cultivars are framed in green and purple lines respectively. Reconstructed genotypes are framed in red. Red arrows point to the supposed offspring. Dashed lines represent doubtful parent–offspring relationships.

The reconstructed HS clusters of Nebbiolo involved other important grape varieties (Fig. 4). According to this hypothesis, Nebbiolo rosé, Pignola and Rossola nera fit as Nebbiolo’s full-siblings, in agreement with IBD coefficients (Supplementary Table S6). Pignola and Rossola nera are both typical of Valtellina (the alpine grape growing region of Lombardy, Fig. 1), but the former variety was also spread in north east Piedmont under the name of Pignolo spano. Nebbiolo rosè, once believed to be a Nebbiolo’s sub-variety but later identified as a different seedling^48^, is present wherever Nebbiolo is grown, including Valtellina, where it is called Chiavennaschino. The role of Nebbiolo’s half-siblings is played by Refosco nostrano, Marzemino, Refosco dal peduncolo rosso, Ortrugo (the latter not confirmed in the cluster of genotype #6 by SNP data), Spergola, Rossoletta and Orsanella. The first three mentioned varieties are typical of north eastern Italy, Ortrugo and Spergola of western Emilia Romagna, while Rossoletta and Orsanella are minor grapes of the central foot Alps. According to this assumed family tree, Teroldego, the popular specialty of Trentino (the alpine region on Italy north east), might also be Nebbiolo’s half-sibling, and in some ways linked to Pinot (Fig. 4).

### Main founder’s historic background

The present study strongly emphasizes the key role of cultivars that are rare, and often endangered, in the development of the present-day varietal assortment. Malvasia aromatica di Parma, Moissan, Bottagera (false), and Coccalona nera are unimportant grapes today, but would have been widespread or intensely cultivated in the past.

Malvasia aromatica di Parma seems the closest fit to the Malvasia bianca, shown to be in Piedmont from the XVI Century and definitely not later than early 1900. It was Sannino^49^ who first realized the morphology of its flowers. Later lost from Piedmont, it luckily survived under the name of Malvasia odorosissima (“highly fragrant Malvasia”) around Parma^50^, where it has been more recently brought back to cultivation.

Moissan was likely identified as the Mauzanetto (“small Moissan”), described by Croce^51^ emphasizing the red stem bunch, a feature inherited by its descendants.

Bottagera (false) recovered under the name of Bottagera in Valtellina (north Lombardy) had apparently disappeared from Piedmont. As already mentioned, the name Bottagera is questionable, so the history of this genotype is still unknown. However, one would expect it to have been widespread in the past in northern Italy, since it gave rise to a large number of cultivars geographically distributed across a large area from Lombardy to central and western Piedmont.

Coccalona nera, highly neglected today, because of the revealed synonyms and the large geographic distribution of its offspring or relatives, was likely prevalent across a large area in previous times. Among the relevant PO related to this grape are Barbera and Riesling italico, the latter better known as Welschriesling in Austria and Graševina or Olasz Rizling in the Balkan area. Our findings excluded Barbera and Graševina as putative parents of Coccalona, whose origin seems to lie in the crossing of Bottagera (false) with a genotype (#1) reconstructed by Colony (Fig. 3). Hence Barbera and Graševina are both Coccalona’s progeny. It is unlikely they are full siblings (Supplementary Table S6), but more likely they are half-siblings with only the parent Coccalona in common. The absence of historical references for this ancestor (likely much widespread in the past) might be due to the inferior quality of its grapes that in all likelihood went into the blend for ordinary rather than varietal wines. Both in Germany and Italy, Coccalona nera was considered a disgusting “wine ruin”, worthy of eliminating from cultivation^37,39^. It is certainly not uncommon that genotypes of bad grape quality turn out to give rise to premium varieties.

### Variety’s presumed geographic origin

The discovery of a grape variety’s geographic origin, especially when referring to traditional renowned wine grapes, is significant for the enhancement and marketing of wines and wine territories. In this regard, the detection of cultivar genetic origin opens the way to speculate on geographic implications. An ancestor’s geographic distribution, especially when retrieved from historic documents, allows one to assume the possible place of birth of descendants. In this work, all the resulting parentages (Table 2) likely occurred in Piedmont or in the neighboring areas, except for reference pedigree. A more precise geographic origin (as for example close to the alpine range, or into the south of the region) might sometimes be speculated. This evidence is not without interest in a region studded with more than 60 PDO wines, based on various local, traditional varieties—mostly highly reputed.

Dolcetto, the third most common variety by surface area in Piedmont covering 6,000 ha, resulted the offspring of Moissan and Dolcetto bianco (i.e. White Dolcetto, but obviously not Dolcetto’s colour variant). Historical documentation locates Moissan and its other descendants in the most western part of Piedmont, and in the region of Liguria nearby. Dolcetto bianco would have been grown in a more limited area in the South of Piedmont. Hence the likely cradle for Dolcetto would correspond to a region where the parents’ distribution areas overlap, and this broadly occurs in the present Langhe (Fig. 1). This inference also correlates with the place where Dolcetto was firstly mentioned in 1593: Dogliani (Cuneo province).

Coccalona nera was supposed to be born from Bottagera and an un-known genotype that, in turn, generated cultivars typical of both sides of the northern Apennines (Albanina nera, Canaiolo nero and Vermentino nero) (Fig. 3). When combining the geographic distribution of Coccalona nera, its synonyms and progeny, it follows this grape was spread across the central part of northern Italy, from the north Apennine range to the foot-Alps. It is not known whether it was also cultivated in the Swiss territory (as the German synonym Schweizertraube suggests) or its presence beyond the Alps remained limited to the region of today's Baden-Württenberg in Germany. The historical synonyms Wuellewälsch and Zottelwelscher mentioned in that area^52^ would seem to indicate a possible origin in the south, as the terms "wälsch" or “welsch” let assume. Similarly, an origin from the southern side of the Alps could be inferred for Coccalona’s progeny Riesling italico, called Welschriesling in Germany and Austria. The absence of its second parent prevent us from establishing if its cradle was central Europe or north western Italy. Here, Riesling italico could be identified with the local ancient “Trebbianino dall’occhio” (“Little Trebbiano with the eye”), term that refers to the much evident pistil scar on the berry. The Coccalona nera PO relationship seems to exclude its cradle from the Balkans, unless Coccalona would have been present there, an un-proved growing place as far as we know.

The genetic origin of Lambrusca di Alessandria suggests its homeland. It is attested that the traditional area of cultivation of its parents Neretto di Marengo and Crovìn is in south eastern Piedmont and the neighboring region of Liguria respectively. This suggests these areas are the cradle of Lambrusca. Its name, like that of Lambrusco (grasparossa, salamino, di Sorbara, etc.), refers to *Labruscae*, the Latin designation of wild or feral grapevines. Some features, like vine endurance and grape content high in colour and tannins, are in fact in common between *V. vinifera* subsp. *sylvestris* and cultivated Lambrusco or Lambrusca di Alessandria. But this last grape is genetically very far from Lambrusco.

The inferred sibship clusters of Nebbiolo (Fig. 4) would suggest a possible geographic origin for this variety on the southern side of central foot-Alps area. Although based on reconstructed genotypes (not included in the sample set, and possibly extinct), the hypothetical pedigree network of Nebbiolo is composed of varieties from northern Italy. These varieties are geographically associated with the central foothills of the Alps area, on the southern alpine side, as also speculated by Gambino et al.^53^.

Our findings add further clarification points about the homeland of Spergola, another variety genetically linked to Nebbiolo. Spergola’s area of cultivation is the northern part of the Apennine range. Nevertheless Spergola resulted identical to Vernaccia di Oristano, considered an historical grape from Sardinia^54^. Spergola-Vernaccia is also PO related to Citronino and Rapallino, two minor grapes attested in Piedmont and in the neighboring Liguria respectively (Supplementary Table S5). All these findings would suggest that Spergola’s cradle must have been the Italian mainland rather than Sardinia.

## Discussion

Genealogical reconstruction of grape varieties has been the goal of numerous studies, either done on a large scale^14,19,27,28^, related to individual cultivars^12,55^, or even to specific geographic areas^9,56,57^. This is the first systematic analysis concerning northwestern Italy, a region particularly renowned for its wines. Robinson et al.^47^ proposed pedigree diagrams for many wine grapes around the world. Due to the large number of genotypes involved, our work revealed an extremely complex network of genetic relations. This network confirms a scenario already described for other European wine areas, where few main genitors played a key role in the origin of other traditional varieties. On the Iberian Peninsula, Marufo, Hebén and Alfrocheiro produced a large component of the local cultivated germplasm^58–60^. Cabernet franc and Sauvignon blanc were indicated as ancestors of many French south western varieties^11^. The copious progeny of Savagnin, Pinot and/or Gouais blanc (Weisser Heunisch) includes many present-day cultivars from central and eastern France, central Europe and the Balkan peninsula^19,61^. In the last region there has been a further contribution as genitors of Coarna alba, Vulpea, and a few others^9,14,27^.

Explanations for such pattern frequently repeated in different areas are not completely clear. Some varieties could have had higher chances of generating a larger offspring number because of their ancient and widespread cultivation or because of the transmission of favorable characters (e.g. plantlet vigour).

Quite a few of the mentioned European genetic founders, namely Marufo, Hebén, Corna alba, exhibit female flowers. So too does one of the main genitors shown in our work, Malvasia aromatica di Parma. A number of possible reasons can account for these varieties being successful as mothers. Female varieties could be, on average, more ancient (having more chances to generate offspring), since hermaphroditism was one of the traits selected during domestication. Moreover, a female plant’s progeny derives from outcrossing only, and it is expected to be less prone to inbreeding depression. Finally there is also the fascinating, but unproven role, of grape breeders played by viticulturists centuries ago (monks in abbeys?) and the ease of pollinating a female inflorescence, hence the choice to use a female individual as a genitor.

Local grapevine resources have often proved to be a great source of unexplored genetic diversity^62^. Minor or even currently neglected grapes were shown to be the founders of present-day renowned cultivars, as also emerged by other studies^11–13^. Local germplasm collection and preservation is therefore an essential step for retrace grape and wine history of a region. In Piedmont, the genetic contribution from varieties introduced from abroad seems limited to a few genotypes (Table 3, Supplementary Figure S1). Among these Moscato bianco (alias White Muscat or Muscat à petits grains blancs), a vine of unknown origin widely planted along the Mediterranean basin and central Europe, and noted in Piedmont since the early XIV century (Table 3). No other historic grapes crucial for the varietal assortment developed in the Italian peninsula, such as Garganega, Mantonico bianco, Sangiovese and Sciaccarello^7,28,55,63^, seem to affect the north western domesticated population.

It might be reasonable to consider the contribution of Gouais blanc, one of the principal ancestors of French traditional grapes^61^, to the origin of several varieties from Piedmont (Table 2). With local names such as Liseiret, Preveyral and Lisöra^64^, this ancient founder of western European viticulture has been present in northern Italy for a long time. A similar consideration might also apply to the historic French Chatus, long cultivated under local names in the Italian alpine foothills^65^.

Some investigated varieties, like Verdea, Pelaverga piccolo, Quagliano and Favorita (syn. Vermentino), were genetically unrelated to the sample set from north western Italy. So, we can assume an outside origin for them. However, it is most likely Favorita/Vermentino was introduced in ancient times, being documented in Piedmont as early as 1658 under the name of Fermentino^66^. Quagliano, grown in the south western pocket of the region, is identical to Bouteillan from Provence in France. This same grape might have migrated from (or to?) the southeast of Italy, where it was found under the local name of Arciprete^67^ and where several local varieties were revealed as its offspring^27,68^. A link with the Greece’s colonization in ancient times of both southern Italy (*Magna Graecia*) and Marseilles, in Provence, is a fascinating yet un-proven possibility.

Recent paleogenomic analyses of grape seeds from French archeological sites indicated direct genetic links between cultivars from Roman period and present varieties^69^, reinforcing the evidence that few generations, if not one, separate many cultivars of today from those of ancient times. This would imply a considerable age for the putative parents of the centuries-old varieties revealed by our study.

The combination of SSRs and SNPs in the investigation of grapevine parentages presents an interesting opportunity. Although the recent application of SNPs to grapevine genotyping showed clear advantages^24,26^, up to now most reference genetic profiles have consisted of SSR markers which have been widely used over the last 20 years^25^. Available *Vitis* genetic databases and literature, as well as internal laboratory databases are, in fact, mostly based on SSR markers. Hence the first steps of kinship analyses, as well as a variety’s true-to-type controls were performed relying on SSRs. Variety trueness to type establishment is crucial for further insights into parentage^9,10^. After checking synonyms through SSR markers, in our work varietal identity of the examined genotypes was established based on plant morphology, historical documents, ampelographic descriptions and local surveys. Genotypes were labeled as “unknown” when varietal identity could not be achieved.

In our analyses SNP markers strengthened kinship evidence and reinforced outcomes especially when related to second-degree relationships. Direct relationships (duos and trios) were consistently detected by the two marker types (Tables 2, Supplementary S6), while some discrepancies remain regarding full-sib relations. For most of those ambiguous cases, FS relations appeared unlikely considering the low k2 IBD coefficient inferred by SNPs (e.g. Fumin-Neyret, Fumin-Petit rouge, Gamba rossa-Calora bianca and Syrah-Viognier). This might indicate that the high number of SNP loci was more effective in capturing the genetic diversity between those pairs of related individuals.

As to the debated FS relationship between Syrah and Viognier proposed by Myles et al*.*^19^ but rejected by other authors^47^, a full-sibling link was an eligible consideration as revealed by statistics related to SSR data (although with a very low probability, Supplementary Table S6). The SNP IBD coefficients showed clearer evidence for higher degree relationship. Meanwhile our findings (data not shown) definitely excluded a PO link between Viognier and Dureza, one of Syrah’s genitor. Similarly, the outcomes of both marker sets did not completely resolve whether Arneis and Bottagera are full-siblings, half-siblings, grandparent–grandchild or avuncular.

Pedigree reconstruction through half-sib clustering had a limited application on plant genetics up to now^70,71^, mainly due to the high computational demand. This approach is particularly useful for studying grapevine genealogy, because ancient key varieties could be extinct or just not yet recovered. Recently, pedigree reconstruction has been applied for outlining the origin of grape varieties from northeastern Italy^57^. In our work three reconstructed genitors usefully completed the pedigree network linking most of the related varieties. Further four reconstructed genotypes helped to draw Nebbiolo’s hypothetical genetic group. It is worth noting we investigated as many as 186 unique recovered varieties in a relatively limited area, mostly from centuries-old, rare local germplasm; some ancestors might have become lost.

Both SNP and SSR markers were used to define sibship clusters. Reconstructed SSR profiles were used to test additional PO relations to corroborate half-sib relationships and uncover putative grandparents. This has produced, for example, solid evidence for the role of reconstructed genotype #2 as descendants from Nebbiolo and Bottagera and genitor of Malvasia aromatica di Parma, Rastajola and others (Table 4). Moreover, the grandparent–grandchild relationships between Nebbiolo, Bottagera and the progeny of genotype #2 were confirmed by counting SNP Mendelian errors in an extended pedigree (offspring, known parent and two candidate grandparents), which does not rely on probabilistic genotypes inferred by Colony.

The four most relevant chlorotypes found within the *V. vinifera* cultivated gene pool^22,72,73^ were detected in 235 investigated grapevine cultivars mostly from northwestern Italy. Their distribution follows the pattern reported for the Italian peninsula^20^, including a high predominance of haplotype D, shared with Sangiovese and Vermentino among the varieties of presumed Italian origin, with Cabernet franc and with Sauvignon for French varieties. It also correlates with the minor contribution of haplotype A, typical of the wild *vinifera* from western and central Europe and of wine cultivars from the same areas such as Pinot, Riesling and Syrah.

In comparison to the chlorotype profile reported for cultivars from France, central Europe and Balkans, Italy shows a negligible impact of type C. This is related just to Gouais blanc (present in the area for long times) and to its local descendants. It is even more true for the northwestern region of Piedmont. The major genetic influence of Gouais, as an ancestor of a large number of progeny in France and Central Europe, may have played a role in spreading chlorotype C in those areas. Due to the maternal inheritance of chloroplast markers, it was possible to infer the male or the female contribution of genitors in a few parentages (Tables 2; Supplementary Figure S1). Due to the prevalence of halotype D, female and male parent could not be identified in most other family trios.

### Conclusive remarks

This work has demonstrated that the combination of different markers as nSSRs and SNPs can give reliable results in a parentage study. The probabilistic reconstruction of “missing links” (cross-validated by independent SSR and SNP analyses) appears promising in grapevine genealogy studies: the extinction of some key genotypes is highly probable within a long-cultivated gene pool.

The recovery of every possible local variety, even the severely neglected or currently nameless, turned to be crucial to reconstructing the greatest number of genetic relationships between cultivars, including those of high relevance today. Therefore, the activities of endangered resources collection and preservation, implemented across grape growing regions, would greatly contribute not only to the conservation of threatened genetic resources, but also to variety parentage studies. The act of uncovering a grape’s historical background (genetic origin, place of birth, migrations, ancient use, etc.) remains of great appeal in promoting wines, especially within the context of developing better engagement marketing oriented to grape variety and wine culture dissemination.

The disclosure of genetic relations between the cultivated gene pool examined in this work and the one of other European wine areas, might be a further development of this study, as the reconstructed parentage of Nebbiolo suggests.

## Materials and methods

### Plant material

The genetic relationships of 186 unique grape *vinifera* cultivars from Piedmont (the foot-Alps region in north western Italy, Fig. 1) were investigated (Supplementary Table S1). According to their cultural relevance, the set of examined genotypes included major, minor, rare, neglected varieties, and even those presently in danger of extinction. They are all traditional cultivars of presumed local origin or cultivated in the region since long ago. None was a modern variety issued from breeding or a grape recently introduced from abroad.

All the examined accessions are grown in an ex-situ germplasm collection located in Grinzane Cavour (Piedmont) (https://www.ipsp.cnr.it/grape-collection/?lang=en). Here major plant morphological traits were observed that focused on recognizing inherited traits supporting parentage and kinship. Moreover, these observations were addressed to outline leaf and bunch morphology. This proved useful to confirm the identification with historical descriptions and plates.

Historical documents quoting or describing grape varieties involved in the genealogy study were examined to retrace the most reliable and/or the most probable history of each cultivar. Synonyms, areas of cultivation in the past, eventual migrations from and to other regions, the most significant traits, the likely age by the earliest quotations were examined. All this information greatly enhanced discussion and interpretation of genetic evidence of grape parentage and kinship.

More than six-hundred (600) *V. vinifera* cultivars from other regions, mainly surrounding Piedmont, were also included in the SSR genotyped sample set, in order to avoid neglecting any possible kinship disclosure with varieties grown elsewhere.

### Genotyping and data curation

DNA was extracted from young green tissues or from woody canes following the procedure described by Thomas et al.^74^.

Nuclear SSR were applied in analyzing from 9 up to 32 loci depending on the set of samples as specified below. The first 9 SSR markers were the ones recommended by the international scientific community and include: the 6 loci selected as genetic descriptors for *Vitis*^75^, and the 3 markers suggested as common reference for grape variety identification within the European *Vitis* Database (www.eu-vitis.de—“Descriptors/file format—detailed SSR-marker specific information”). The other nSSRs were chosen according to polymorphism, stability and their membership of different linkage groups (Supplementary Table S8). nSSR analysis conditions were according to Ruffa et al.^43^.

Four chloroplast SSR (cpSSR) markers, enough to identify the eight major chlorotypes present in *Vitis vinifera* germplasm^26^, were analysed according to the protocol used for nSSR described by Ruffa et al.^43^.

Around 2/3 of the samples (Supplementary Table S1) were also genotyped by the Illumina Infinium 20 K Chip developed by the GrapeReSeq Consortium^30^. Genotypes were called using the standard procedure for diploids implemented in GenomeStudio (Illumina Inc., San Diego, CA, USA). SNP markers with missingness rate > 1% and minor allele frequency (MAF) < 5% were removed. After SNP curation, missingness of individuals was checked and individuals with more than 1% of missing values were excluded. Data curation resulted in a dataset containing 140 individuals and 11,419 nuclear SNPs—this was used for pedigree reconstruction.

Moreover, genotype calling for 24 plastidial SNPs was manually curated. This was done to account for the haploid nature of the chloroplast genome. After curation, a set of four plastidial SNPs were polymorphic and uncorrelated to other markers. The selected plastidial SNPs combined with the cpSSR were used to infer chlorotype in 235 out of 272 examined varieties (Supplementary Table S1) and for inference of father- or motherhood.

### nSSR stepwise sample set definition

1. Putative POs at 9 nSSR loci. The 9 international nSSR markers were assayed on the 792 grape varieties examined in this study (186 collected or cultivated in Piedmont, and 606 from other regions). Where possible, several accessions for each variety were analyzed, producing a genetic consensus profile.
2. Putative POs at 20 nSSR loci. Grape cultivars sharing at least one allele at 9 nSSR markers were then analyzed at further 11 loci (totally 20 loci), discarding at this step all the genotypes showing less than 19 common alleles.
3. Putative POs at 32 nSSR loci. Further 12 nSSR loci (totally 32 markers, well distributed on the different linkage groups) were assayed on a set of 227 genotypes (Supplementary Table S3) consisting of:
  196 cvs (out of the 186 + 606) resulted putatively linked by PO at steps 1 and 2;
  12 genotypes (used as references for family relationships) notoriously related from prior publications based on molecular markers and/or from confirmed breeders’ notations: Cabernet franc, Cabernet Sauvignon, Charmont, Chasselas blanc, Garanoir, Heptakilo, Moscato d’Amburgo, Perla di Csaba, Pinot, Sauvignon blanc, Savagnin, Sciaccarello (indicated in bold in Supplementary Tables S1, S3);
  19 outgroup *vinifera* cultivars selected among the unrelated by direct genetic relationship and belonging to different and various geographic pools (indicated in italic in Supplementary Tables S1, S3).

### Pedigree reconstruction

In the present study, pedigree reconstruction of ancient grape varieties was based on both SSR and SNP markers. Different approaches were used for the two marker sets, due to the different characteristics of SSR and SNPs and to the difference in the relative dataset sizes.

For nSSR markers, parent/offspring relationships and full parentage (family trios) were investigated by the exclusion method (based on counts of Mendelian inconsistencies) and by the likelihood-based method implemented in the software Cervus^76^. Additionally, information from second-degree relationships was used via the multi-generation likelihood method of Colony^77^. By using this approach parent/offspring (PO), full sib (FS) and half sib (HS) relations were simultaneously inferred in order to identify the highest-likelihood pedigree configuration. Colony parameters were set up in order to perform one medium-length run with the full likelihood method, allelic dropout rate 0 and other genotyping error rate 0.0050. Although the occurrence of selfing is rare in grapevine, an inbreeding model has been chosen to account for parental relatedness and population structure. Reconstructed parental genotypes were tested for additional PO relationships.

As for the SNP dataset, identity by descent (IBD) Cotterman’s coefficients were estimated using PLINK’s Method of Moments^78^, in order to detect PO relationships. Following the PCA-based approach of Morrison^79^, we removed ancestrally informative markers and checked that our sample genetic structure did not represent a bias for the PLINK estimator. Then, family trios were identified using the exclusion method based on the count of Mendelian errors. Due to high computational demand, Colony sibship clustering was not performed on the whole SNP dataset, rather on a subset of individuals resulting in HS relationships by the SSR analysis, with the main aim of supporting those results. A long run (length = 3) with the full likelihood method, the highest likelihood precision and a genotyping error rate of 0.00015 was performed. Allele frequencies observed in the whole dataset (140 individuals) were provided as known. The exclusion method based on Mendelian errors was used to test all possible pairs of grandparents (from a list of candidate second degree relatives or HS-like) for a certain offspring, in a pedigree including offspring, known parent and two candidate grandparents (putative parents of the un-genotyped parent).

## Supplementary information

Supplementary file1

Supplementary file2
