# Supplementary material for: DNA-based genealogy reconstruction of Nebbiolo, Barbera and other ancient grapevine cultivars from northwestern Italy
Source: Sci Rep. 2020 Sep 25;10:15782. doi: 10.1038/s41598-020-72799-6 (PMC7519648; doi:10.1038/s41598-020-72799-6)

# DNA-based genealogy reconstruction of Nebbiolo, Barbera and other ancient grapevine cultivars from northwestern Italy

Stefano Raimondi[1], Giorgio Tumino[2], Paola Ruffa[1,3], Paolo Boccacci[1], Giorgio Gambino[1], Anna Schneider[1]*

1 National Research Council of Italy – Institute for Sustainable Plant Protection (CNR-IPSP), Strada delle Cacce 73 - 10135 Torino (Italy)

2 Council for Agricultural Research and Economics - Research Centre for Genomics and Bioinformatics, Via San Protaso 302, 29017 - Fiorenzuola d'Arda (PC), Italy

3 University of Turin - Department of Agricultural, Forest and Food Sciences (UNITO-DiSAFA), L. Braccini 2 - 10095 Grugliasco, Torino (Italy)

* Corresponding author: anna.schneider@ipsp.cnr.it

Table S1 - List of the grape cultivars analysed in this study, their major features, places of collection or cultivation and whether they also genotyped by SNPs. The list includes all the examined varieties from Piedmont (186), those from other regions genetically related, some cultivars choosen as an outgroup (in italic) and 12 varieties as references for relationships (in bold) for a total of 272 genotypes. Specifications in brackets (when they do not indicate synonyms) distinguish homonym varieties usually by their collecting places. Cultivar names followed by the initial of berry colour (B.= white, N. =black, Rs.=rose) are included into the National Italian Catalogue. Berry color is coded as follows: b=white, n=black, r=red, rs=rose, v=violet. Sex of the flower may be H=hermafrodite or F=female. Current planting relevance is indicated as Major: > 100 ha, Minor: < 100 ha, Local: < 10 ha, Endangered: few plants.

| **Cultivar name** | **Berry color** | **Sex of the flower** | **Berry flavour** | **Area of recovery / cultivation** | **Current planting relevance** | **Number of analysed accessions** | **Observations on vine morphology** | **Analysed at 32 nSSR loci** | **Analysed also by the SNP 20K Illumina chip** | **Chlorotype** |
|---|---|---|---|---|---|---|---|---|---|---|
| *Albana B.* | b | H | None | Emilia-Romagna | Major | 2 | yes | yes | yes | D |
| Albanina nera | n | - | None | Emilia-Romagna | Endangered | 1 | no | yes | no | D |
| *Albarola B.* | b | H | None | Liguria | Major | 6 | yes | yes | yes | D |
| Aleatico (false, from Paderna) | r | H | Muscat type | Piedmont (south-east) | Endangered | 1 | yes | yes | no | D |
| Aleatico N. | n | H | Muscat type | Central and Southern Italy | Major | 7 | yes | yes | no | A |
| Arneis B. | b | H | None | Piedmont (Roero) | Major | 2 | yes | yes | yes | D |
| Arsetto bianco (presumed) | b | H | Muscat type | Piedmont (Monferrato) | Endangered | 1 | no | no | no | - |
| Avanà N. | n | H | None | Piedmont (west) | Local | 2 | yes | yes | yes | A |
| Avanà rosso | n | H | None | Piedmont (west) | Endangered | 1 | yes | no | no | D |
| Avarenchetto | n | H | None | Piedmont (west) | Endangered | 1 | yes | yes | no | C |
| Avarengo N. | n | H | None | Piedmont (west) | Local | 2 | yes | yes | yes | D |
| Balsamina (from Reggio Emilia area) | n | H | None | Emilia-Romagna | Endangered | 1 | yes | yes | no | A |
| Baratuciat B. | b | H | None | Piedmont (west) | Local | 2 | yes | yes | yes | A |
| Barbarossa di Piemonte | r | H | None | Piedmont (Monferrato) | Endangered | 2 | yes | yes | yes | D |
| Barbassese | b | H | None | Piedmont (south-east) | Endangered | 1 | yes | yes | yes | D |
| Barbera bianca B. | b | H | None | Piedmont (south-east) | Local | 2 | yes | yes | yes | D |
| Barbera ciarìa | n | H | None | Piedmont (Roero) | Endangered | 1 | yes | yes | no | A |
| Barbera 'd Davi | n | H | None | Piedmont (west) | Endangered | 1 | yes | no | no | D |
| Barbera di Patrunat | n | H | None | Piedmont (Roero) | Endangered | 1 | yes | yes | no | D |
| Barbera N. | n | H | None | Piedmont, many places in Italy | Major | 8 | yes | yes | yes | D |
| Barberùn | n | H | None | Piedmont (north) | Endangered | 1 | yes | yes | no | D |
| Barbrassa | n | H | None | Piedmont (west) | Endangered | 1 | yes | yes | no | D |
| Becuet N. (Persan N) | n | H | None | Piedmont (west), France | Local | 2 | yes | no | no | A |
| Berla grossa | n | H | None | Piedmont (west) | Endangered | 3 | yes | yes | no | C |
| *Besgano bianco* | b | - | None | Emilia-Romagna | Endangered | 2 | no | yes | no | - |
| Bian ver B. | b | H | None | Piedmont (west), France | Local | 1 | yes | no | no | D |
| Bianchetta (from Frascata) | b | H | None | Piedmont (south-east) | Endangered | 1 | yes | yes | no | D |
| Bianchetto (from Saluzzo) | b | H | None | Piedmont (west) | Endangered | 1 | yes | yes | yes | D |
| Bibiola | n | H | None | Piedmont (west) | Endangered | 1 | yes | yes | yes | D |
| Biestro (presumed) | b | - | None | Liguria | Endangered | 1 | no | yes | no | D |
| Blanchet | b | H | None | Piedmont (west) | Endangered | 1 | yes | yes | no | D |
| Bonarda N. | n | H | None | Piedmont (Monferrato) | Major | 3 | yes | yes | yes | D |
| Bonardina | n | H | None | Piedmont (west) | Endangered | 2 | yes | yes | yes | D |
| Bonda N. | n | H | None | Piedmont (north) | Local | 2 | yes | yes | no | D |
| Bondola | n | H | None | Piedmont (north) | Local | 2 | yes | yes | yes | D |
| Bordò | n | H | Muscat type | Piedmont (Monferrato) | Endangered | 3 | yes | yes | yes | D |
| *Bosco B.* | b | H | None | Liguria | Minor | 3 | yes | yes | yes | D |
| Bottagera (false) | n | H | None | Valtellina | Endangered | 1 | yes | yes | yes | A |
| Bracciola nera N. | n | H | None | Liguria | Local | 1 | yes | yes | no | D |
| Brachetto (false, from Nizza M.to) | v | H | Muscat type | Piedmont (Monferrato) | Endangered | 3 | yes | yes | yes | D |
| Brachetto Migliardi | n | H | Muscat type | Piedmont (south-east) | Local | 1 | yes | yes | yes | D |
| Brachetto N. | n | H | Muscat type | Piedmont (south-east) | Major | 2 | yes | yes | yes | D |
| Bragat rosa N. | r | H | Muscat type | Piedmont (Roero) | Local | 1 | yes | yes | yes | D |
| Bressana | n | H | None | Valtellina | Endangered | 2 | yes | yes | yes | D |
| Brugnola | n | H | None | Valtellina | Local | 1 | yes | yes | yes | D |
| Brunetta (from Rivoli) | n | H | None | Piedmont (west) | Endangered | 3 | yes | yes | no | D |
| Bubbierasco | n | H | None | Piedmont (west) | Endangered | 2 | yes | yes | yes | D |
| Bubia | n | H | None | Piedmont (west) | Endangered | 1 | yes | no | no | D |
| **Cabernet franc N.** | n | H | Herbaceous | France, International | Major | 9 | yes | yes | yes | D |
| **Cabernet sauvignon N.** | n | H | Herbaceous | France, International | Major | 17 | yes | yes | yes | D |
| Cagambraga | rs | H | None | Piedmont (north) | Endangered | 1 | yes | yes | yes | D |
| Calora (false) | n | H | None | Piedmont (south-east), Oltrepò pavese | Endangered | 2 | no | no | no | D |
| Calora (presumed) | v | H | None | Piedmont (south-east) | Endangered | 2 | no | yes | no | - |
| Calora bianca (presumed) | b | F | None | Piedmont (south-east) | Endangered | 1 | no | yes | yes | D |
| *Canaiolo nero N.* | n | H | None | Tuscany | Major | 4 | yes | yes | yes | A |
| Cardìn | n | H | None | Piedmont (west) | Local | 3 | yes | yes | yes | D |
| Cascarolo | b | H | None | Piedmont (Monferrato) | Local | 1 | yes | yes | yes | A |
| Cavazzina | n | - | None | Emilia-Romagna | Endangered | 2 | yes | yes | no | D |
| Chardonnay B. | b | H | None | International | Major | 3 | yes | yes | yes | C |
| **Charmont B.** | b | H | None | Switzerland | Local | 1 | yes | yes | yes | D |
| **Chasselas blanc B.** | b | H | None | Switzerland, Central Europe | Major | 16 | yes | yes | yes | D |
| Chatus N. | n | H | None | Piedmont, France | Local | 4 | yes | yes | yes | D |
| Chiavennasca bianca | b | H | None | Piedmont (north), Valtellina | Endangered | 4 | yes | yes | yes | D |
| Ciamasul | n | H | None | Piedmont (west) | Endangered | 1 | yes | no | no | A |
| Cipriana (Apesorgia nera) | n | H | None | Sardinia, Piedmont (north) | Local | 2 | yes | no | no | - |
| Citronino | b | H | None | Piedmont (south-east) | Endangered | 2 | yes | yes | yes | D |
| Coccalona bianca | b | H | None | Piedmont (south-east) | Endangered | 2 | yes | yes | no | D |
| Coccalona nera (presumed, Rohrtraube blaurot) | n | H | None | Piedmont (south-east) | Endangered | 3 | yes | yes | yes | A |
| Corbeau N. | n | H | None | France, Piedmont (south-east) | Major | 3 | yes | yes | no | - |
| Cortese B. | b | H | None | Piedmont (Monferrato) | Major | 4 | yes | yes | yes | A |
| Croatina N. | n | H | None | Piedmont, Oltrepò pavese | Major | 1 | yes | yes | yes | D |
| Croetto | n | H | None | Liguria | Endangered | 3 | yes | yes | no | D |
| Crova (elliptic berry) (Uva crova) | n | H | None | Oltrepò pavese | Endangered | 2 | yes | yes | no | D |
| Crovaro | n | H | None | Piedmont (south-east) | Endangered | 2 | yes | yes | no | D |
| Crovin (from Perti) | n | H | None | Liguria | Endangered | 2 | yes | yes | yes | D |
| Dolcetto bianco | b | H | None | Piedmont (Langhe) | Endangered | 1 | yes | yes | yes | D |
| Dolcetto N. | n | H | None | Piedmont (Langhe) | Major | 6 | yes | yes | yes | D |
| Doux d'Henry N. | n | F | None | Piedmont (west) | Local | 2 | yes | yes | yes | D |
| Durà bianco (presumed) | b | H | None | Piedmont (south-east) | Endangered | 1 | yes | no | no | - |
| Durasa N. | n | H | None | Piedmont (north and south-east) | Local | 4 | yes | yes | yes | D |
| Erbaluce B. | b | H | None | Piedmont (north) | Major | 3 | yes | yes | yes | D |
| Feral accession | n.d. | - | None | Piedmont (south-east) | Endangered | 1 | no | yes | no | D |
| Fortana N. | n | H | None | Emilia-Romagna | Major | 4 | yes | yes | yes | D |
| Freisa (false) | n | H | None | Piedmont (south-east) | Endangered | 1 | no | no | no | - |
| Freisa N. | n | H | None | Piedmont (Monferrato) | Major | 6 | yes | yes | yes | D |
| Fument | n | H | None | Piedmont (west) | Endangered | 1 | no | no | no | - |
| Fumin N. | n | H | None | Aosta valley | Minor | 2 | yes | yes | yes | D |
| Galletta | n | H | None | Piedmont (Roero) | Endangered | 1 | yes | yes | no | D |
| Galletto | n | H | None | Piedmont (Monferrato) | Endangered | 1 | yes | yes | no | D |
| Gamay N. | n | H | None | France | Major | 4 | yes | yes | yes | C |
| Gamba rossa N. (Gamba di pernice) | n | H | None | Piedmont (Monferrato) | Local | 3 | yes | yes | yes | D |
| **Garanoir N.** | n | H | None | Switzerland | Major | 2 | yes | yes | yes | C |
| *Garganega B.* | b | H | None | Italy (north and south) | Major | 3 | yes | yes | yes | D |
| Giamellotto (false) | n | H | None | Piedmont (west) | Endangered | 2 | yes | yes | no | D |
| Gouais blanc (Liseiret B.) | b | H | None | Piedmont, Central Europe | Local | 6 | yes | yes | yes | C |
| Granaccia bianca | b | F | None | Liguria | Endangered | 1 | yes | yes | no | - |
| Grandurey | n | H | None | Piedmont (west) | Endangered | 3 | yes | yes | no | D |
| Grec rouge | r | H | None | Piedmont, many sites in Europe | Endangered | 4 | yes | yes | yes | A |
| Grignolino N. | n | H | None | Piedmont (Monferrato) | Major | 2 | yes | yes | yes | D |
| Gris | n | H | None | Piedmont (north) | Endangered | 1 | yes | yes | no | D |
| Grisa (from Cumiana) | n | H | None | Piedmont (west) | Endangered | 2 | yes | yes | yes | D |
| Grò blan | b | H | None | Piedmont (west) | Endangered | 1 | yes | no | no | D |
| Groppello gentile N. | n | H | None | Lombardy | Major | 1 | yes | yes | no | - |
| *Harslevelu* | b | H | None | Hungary | Major | 3 | yes | yes | no | - |
| **Heptakilo** | n | - | None | Mediterranean sea coastal areas | Endangered | 3 | yes | yes | no | - |
| Hibou blanc | b | H | None | Piedmont (west) | Endangered | 1 | yes | no | no | - |
| Invernenga B. | b | H | None | Lombardy, Piedmont (south-east) | Local | 2 | yes | no | no | - |
| Lacrima Cristi | n | H | None | Piedmont (west) | Endangered | 1 | yes | yes | no | D |
| Lambrusca di Alessandria N. | n | H | None | Piedmont (Monferrato) | Local | 3 | yes | yes | yes | D |
| Lambrusca pignata | n | H | None | Piedmont (west) | Endangered | 1 | yes | yes | no | D |
| Lambrusca vittona | n | H | None | Piedmont (west) | Endangered | 2 | yes | yes | no | A |
| Lambruschetta | n | H | None | Piedmont (south-east) | Endangered | 2 | yes | yes | no | D |
| *Lambrusco Barghi N.* | n | - | None | Emilia-Romagna | Minor | 2 | yes | yes | no | A |
| Lambrusco del pellegrino N. (L. di Fiorano) | n | H | None | Emilia-Romagna | Local | 2 | yes | yes | no | A |
| Lambrusco Maestri N. | n | H | None | Emilia-Romagna | Major | 3 | yes | yes | yes | A |
| *Lambrusco Salamino N.* | n | - | None | Emilia-Romagna | Major | 2 | yes | yes | no | A |
| Luglienga bianca (S. Anna di Lipsia B.) | b | H | None | Piedmont, many sites in Europe | Local | 9 | yes | yes | yes | D |
| Luglienga moscata | b | H | Muscat type | Piedmont (Roero) | Endangered | 1 | yes | yes | no | D |
| Luglienga nera | n | H | None | Piedmont (west) | Endangered | 2 | yes | yes | no | - |
| Luverdon (Canari) | n | H | None | France, Spain, Piedmont (west) | Endangered | 1 | yes | no | no | - |
| Maccaferro (Mornasca N.) | n | H | None | Piedmont (south-east), Oltrepò pavese, Emilia-Romagna | Local | 1 | yes | no | no | D |

| | | | | | | | | | | |
|---|---|---|---|---|---|---|---|---|---|---|
| Maiolina N. | n | H | None | Lombardy and Piedmont | Local | 2 | yes | yes | no | D |
| Malvasia aromatica di Parma (Malvasia Casalini B.) | b | F | Muscat type | Emilia-Romagna | Endangered | 2 | yes | yes | yes | D |
| Malvasia bianca (from Vignale) | b | H | Muscat type | Piedmont (Monferrato and south-east) | Endangered | 1 | yes | yes | no | D |
| *Malvasia bianca lunga B.* | b | H | None | Central and Southern Italy | Major | 13 | yes | yes | yes | D |
| Malvasia di Boca | b | H | Muscat type | Piedmont (north) | Endangered | 5 | yes | yes | no | - |
| Malvasia di Candia aromatica B. | b | H | Muscat type | Emilia-Romagna | Major | 4 | yes | yes | no | D |
| Malvasia di Casorzo N. | n | H | Muscat type | Piedmont (Monferrato) | Minor | 1 | yes | yes | yes | D |
| Malvasia di Schierano N. | n | H | Muscat type | Piedmont (Monferrato) | Minor | 1 | yes | yes | yes | D |
| Malvasia moscata B. | b | H | Muscat type | Piedmont (Monferrato) | Local | 7 | yes | yes | yes | D |
| Malvasia nera (from Costa V.) | n | H | Muscat type | Piedmont (south-east) | Endangered | 2 | yes | yes | no | D |
| Malvasia nera a grappolo corto | n | H | Muscat type | Piedmont (Monferrato) | Endangered | 2 | yes | yes | no | D |
| Malvasia nera lunga N. | n | H | Muscat type | Piedmont (Monferrato) | Minor | 2 | yes | yes | yes | D |
| Marzemino N. | n | H | None | Italy (north-east), Emilia-Romagna | Major | 6 | yes | yes | yes | D |
| Mayolet Rs. | n | H | None | Aosta valley | Local | 1 | yes | yes | yes | D |
| Millegusti | n | F | Muscat type | Piedmont (south-east) | Endangered | 2 | yes | yes | yes | D |
| Moissan | n | H | None | Piedmont (west) | Endangered | 1 | yes | yes | yes | D |
| Montanera (false, from Saluzzo) | n | H | None | Piedmont (west) | Endangered | 1 | no | no | no | - |
| Montanera N. | n | H | None | Piedmont (west), Valtellina | Local | 5 | yes | yes | yes | D |
| Moradella N. | n | H | None | Piedmont (south-east), Lombardy | Local | 1 | yes | yes | yes | D |
| Morio muskat | b | - | Muscat type | Germany | Major | 1 | yes | yes | no | D |
| Moscato bianco B. | b | H | Muscat type | Piedmont, International | Major | 35 | yes | yes | yes | D |
| Moscato bianco precoce | b | H | Muscat type | Piedmont (Monferrato) | Endangered | 1 | no | yes | yes | D |
| **Moscato d'Amburgo N. (Muscat of Hamburg)** | n | H | Muscat type | International | Major | 13 | yes | yes | yes | D |
| Moscato di Scanzo N. | n | H | Muscat type | Lombardy | Local | 2 | yes | yes | yes | D |
| Moscato giallo B. | b | H | Muscat type | Eastern Italy, Balkans, Piedmont | Major | 11 | yes | yes | yes | A |
| Moscato nero (from Borbera valley) | n | H | None | Piedmont (south-east) | Endangered | 1 | yes | yes | no | D |
| Moscato nero di Acqui N. | n | H | Muscat type | Piedmont | Local | 1 | yes | yes | yes | D |
| Moscato rosa Rs. | n | F | Muscat type | North-eastern Italy, Balkans | Minor | 3 | yes | yes | yes | C |
| Moschato mavro | v | H | Muscat type | Balkans | Local | 4 | yes | yes | no | D |
| Mossano (from Canavese area) | n | H | None | Piedmont (north) | Endangered | 3 | yes | yes | no | D |
| Mossano (from Chieri) | n | H | None | Piedmont (Monferrato) | Endangered | 1 | yes | no | no | D |
| Mostarino | v | H | None | Piedmont (south-east), Emilia-Romagna | Endangered | 2 | yes | yes | no | D |
| Mulinè | n | H | None | Piedmont (west) | Endangered | 2 | yes | yes | yes | D |
| Muscat rouge de Madère | r | H | Muscat type | Central Italy, France | Local | 8 | yes | yes | no | D |
| Nascetta B. | b | H | None | Piedmont (Langhe) | Minor | 1 | yes | yes | yes | D |
| Nebbiolo (false) 217 | n | H | None | Piedmont (Langhe) | Endangered | 2 | yes | yes | yes | A |
| Nebbiolo (false, from Aisone) | n | H | None | Piedmont (west) | Endangered | 1 | yes | no | no | - |
| Nebbiolo (false, from Stroppo) | v | H | None | Piedmont (west) | Endangered | 1 | yes | no | no | D |
| Nebbiolo d'Antom | n | H | None | Piedmont (west) | Endangered | 1 | yes | yes | no | D |
| Nebbiolo di Dronero (false) | n | H | None | Piedmont (west) | Endangered | 1 | yes | yes | yes | D |
| Nebbiolo gabardin | n | H | None | Piedmont (west) | Endangered | 1 | yes | yes | no | D |
| Nebbiolo N. | n | H | None | Piedmont, Valtellina | Major | 47 | yes | yes | yes | D |
| Nebbiolo rosé | n | H | None | Piedmont, Valtellina | Minor | 11 | yes | yes | yes | D |
| Nebùe | n | H | None | Piedmont (west) | Endangered | 1 | yes | yes | no | D |
| Negrello | n | H | None | Valtellina | Endangered | 2 | yes | yes | no | D |
| Negrera (from Valtellina) | n | H | None | Valtellina | Endangered | 1 | yes | yes | yes | A |
| Neiran (from Condove) | n | H | None | Piedmont (west) | Endangered | 2 | yes | no | no | D |
| Neirano bianco | b | H | None | Piedmont (Roero) | Endangered | 1 | no | no | no | D |
| Neirano di Spagna | n | H | None | Piedmont (Roero and Langhe) | Endangered | 2 | yes | no | no | D |
| Neirera | n | H | None | Piedmont (Roero) | Endangered | 1 | yes | yes | no | D |
| Ner d'ala N. | n | H | None | Piedmont (north), Aosta Valley | Local | 4 | yes | no | no | D |
| Neretta cuneese N. | n | H | None | Piedmont (west) | Local | 3 | yes | yes | yes | D |
| Neretto di Bairo N. | n | H | None | Piedmont (north) | Local | 3 | yes | yes | yes | D |
| Neretto di Marengo | n | H | None | Piedmont (south-east) | Endangered | 2 | yes | yes | yes | D |
| Neretto di Salto | n | H | None | Piedmont (north) | Endangered | 1 | yes | yes | yes | D |
| Neretto duro (Balau) | n | H | None | Piedmont | Endangered | 5 | yes | yes | yes | D |
| Neretto gentile | n | H | None | Piedmont (north) | Endangered | 2 | yes | no | no | A |
| Neretto nostrano | n | H | None | Piedmont (north) | Endangered | 1 | yes | no | no | A |
| *Neyret N.* | n | H | None | Aosta valley | Minor | 3 | yes | yes | yes | D |
| Orange muscat (M. fleur d'oranger) | b | H | Muscat type | International | Minor | 8 | yes | yes | no | D |
| Orsanella | n | - | None | Lombardy | Endangered | 1 | no | yes | yes | D |
| *Ortrugo B.* | b | H | None | Emilia-Romagna | Major | 2 | yes | yes | yes | D |
| Parmesana | n | H | None | Emilia-Romagna, Lombardy, Piedmont (south-east) | Endangered | 4 | yes | yes | no | D |
| Parporio | n | H | None | Piedmont (west) | Endangered | 1 | yes | no | no | D |
| Passeretta B. | b | H | None | Piedmont (Langhe), Emilia-Romagna | Local | 2 | yes | no | no | D |
| Patlassa (long internodes, presumed) | b | - | None | Piedmont (south-east) | Endangered | 1 | yes | yes | no | D |
| Pelaverga N. | n | H | None | Piedmont (west) | Minor | 5 | yes | yes | yes | A |
| Pelaverga piccolo N. | n | H | None | Piedmont (Langhe) | Local | 3 | yes | yes | yes | D |
| **Perla di Csaba B. (Csaba gyoengye)** | b | H | Muscat type | International | Minor | 6 | yes | yes | yes | C |
| Petit rouge N. | n | H | None | Aosta valley | Major | 3 | yes | yes | yes | D |
| *Petite Arvine B.* | b | H | None | Switzerland, France | Major | 2 | yes | yes | no | - |
| Picolit (false) | b | H | None | Piedmont, some sites in Northern Italy | Local | 1 | yes | yes | no | D |
| Pignola N. | n | H | None | Piedmont (north), Valtellina | Minor | 2 | yes | yes | yes | D |
| **Pinot nero N. (Pinot noir)** | n | H | None | France, International | Major | 10 | yes | yes | yes | A |
| Plassa N. | n | H | None | Piedmont (west) | Local | 2 | yes | yes | yes | A |
| Poliziana (from Vignale) | n | H | None | Piedmont (Monferrato) | Endangered | 1 | no | no | no | - |
| Preveyral nero | n | H | None | Piedmont (west) | Endangered | 1 | yes | no | no | - |
| Prié blanc B. | b | H | None | Aosta valley | Minor | 1 | yes | yes | yes | A |
| Primaticcia | n | H | None | Piedmont (north) | Endangered | 1 | yes | yes | no | D |
| Putet | n | H | None | Piedmont (west) | Endangered | 1 | yes | no | no | A |
| Quagliano N. (Bouteillan) | n | H | None | Piedmont (west) | Local | 4 | yes | yes | yes | A |
| Rachina | n | H | None | Piedmont (north) | Endangered | 1 | yes | yes | no | D |
| Rapallino | b | H | None | Liguria | Endangered | 2 | yes | yes | yes | D |
| Rastajola | n | H | None | Piedmont (north) | Endangered | 3 | yes | yes | yes | D |
| Refosco dal peduncolo rosso N. | n | H | None | Italy (north-east) | Major | 2 | yes | yes | no | - |
| Refosco gentile | n | H | None | Italy (north-east) | Endangered | 3 | yes | yes | no | - |
| Refosco nostrano N. | n | H | None | Italy (north-east) | Major | 1 | yes | yes | no | - |
| Riesling B. | b | H | None | International | Major | 1 | yes | yes | no | A |
| Riesling italico B. | b | H | None | Balkans, Oltrepò pavese, Piedmont (south-east) | Major | 2 | yes | yes | yes | A |
| Rossara N. (Schiava N.) | n | H | None | Piedmont, Lombardy | Minor | 13 | yes | yes | yes | D |
| Rossese bianco (false, from Dogliani) | b | H | None | Piedmont (Langhe) | Endangered | 1 | no | no | no | - |
| Rossese bianco (false, from Monforte) | b | H | None | Piedmont (Langhe) | Endangered | 1 | yes | no | no | D |
| Rossese bianco B. | b | H | None | Piedmont (Langhe) | Local | 2 | yes | yes | no | D |
| Rossola (false) | n | - | None | Valtellina | Endangered | 1 | no | yes | yes | D |
| Rossola nera N. | n | H | None | Valtellina | Local | 6 | yes | yes | yes | D |
| Rossola verde | n | - | None | Valtellina | Endangered | 1 | no | yes | yes | D |
| Rossoletta | v | H | None | Piedmont (north), Valtellina | Endangered | 3 | yes | yes | yes | D |
| Ruché N. | n | H | Muscat type | Piedmont (Monferrato) | Minor | 4 | yes | yes | yes | D |
| Ruietto (from Perosa) | rs | H | None | Piedmont (west) | Endangered | 1 | yes | no | no | - |
| *Sangiovese N.* | n | H | None | Central and Southern Italy | Major | 27 | yes | yes | yes | D |
| **Sauvignon blanc B.** | b | H | None | France, International | Major | 15 | yes | yes | no | - |
| **Savagnin blanc B** | b | H | None | Central Europe | Major | 4 | yes | yes | no | - |
| Schiava gentile N. | n | H | None | Italy (north-east) | Major | 1 | no | yes | no | - |
| **Sciaccarello** | v | H | None | Tuscany, Emilia-Romagna, Liguria, France (Corse) | Major | 5 | yes | yes | yes | D |
| *Scimiscià B.* | b | H | None | Liguria, Emilia-Romagna, France (Corse) | Local | 3 | yes | yes | yes | D |
| Scruss (presumed) | n | F | None | Piedmont (south-east) | Endangered | 1 | no | no | no | D |
| Servavillano | n | F | None | Piedmont (Langhe) | Endangered | 1 | yes | yes | no | D |
| Sgavetta N. | n | H | None | Emilia-Romagna | Local | 2 | yes | yes | no | A |
| Slarina N. | n | H | None | Piedmont (south-east) | Local | 3 | yes | yes | yes | D |
| Spergola B. (Vernaccia di Oristano B.) | b | H | None | Emilia-Romagna | Minor | 3 | yes | yes | yes | D |
| Stciucaera bianca | b | H | None | Piedmont (west), Piedmont (south-east), Emilia-Romagna | Endangered | 3 | no | no | no | D |
| *Syrah N.* | n | H | None | France, International | Major | 15 | yes | yes | yes | A |
| Tadone 1 (from Saluzzo) | n | H | None | Piedmont (west) | Endangered | 1 | yes | yes | yes | D |
| Tadone 2 (from Saluzzo) | n | H | None | Piedmont (west) | Endangered | 1 | yes | yes | yes | D |
| Teinturier ad acino rotondo | n | H | None | Piedmont | Endangered | 2 | yes | yes | yes | A |
| Teinturier du Cher | n | H | None | France | Endangered | 2 | yes | yes | yes | A |
| Teroldego N. | n | H | None | Italy (north-east) | Major | 2 | yes | yes | yes | D |
| *Terrano N.* | n | H | None | Italy (north-east), Slovenia, Croatia | Major | 4 | yes | yes | no | D |
| Timorasso B. | b | H | None | Piedmont (south-east) | Minor | 2 | yes | yes | yes | D |
| Trebbianino di Tortona (presumed) | b | H | None | Piedmont (south-east) | Endangered | 3 | no | yes | yes | D |
| *Trebbiano toscano B.* | b | H | None | Central Italy, France | Major | 10 | yes | yes | yes | D |
| Unknown black (from Fresonara) | n | H | None | Piedmont (south-east) | Endangered | 1 | no | yes | no | A |
| Unknown black (from Monleale) | n | F | None | Piedmont (south-east), Oltrepò pavese | Endangered | 3 | no | no | no | - |
| Unknown black (from Pomaretto) | n | H | None | Piedmont (west) | Endangered | 1 | yes | yes | yes | D |
| Unknown black (from Sale) | n | H | None | Piedmont (south-east) | Endangered | 3 | yes | yes | yes | D |
| Unknown black (from Sarezzano) | n | - | None | Piedmont (south-east), Oltrepò pavese | Endangered | 2 | no | no | no | - |
| Unknown black (from Spineto) | n | F | None | Piedmont (south-east) | Endangered | 2 | yes | yes | yes | D |
| Unknown black (from Tortona) | n | H | None | Piedmont (south-east) | Endangered | 1 | yes | yes | no | D |
| Unknown black 1 (from Abbadia L.) | n | - | None | Lombardy (north) | Endangered | 1 | no | yes | no | D |
| Unknown black 2 (from Abbadia L.) | n | - | None | Lombardy (north) | Endangered | 3 | yes | yes | yes | D |
| Unknown white (from Castellania) | b | H | None | Piedmont (south-east) | Endangered | 1 | no | no | no | - |
| Unknown white (from Paderna) | b | H | None | Piedmont (south-east) | Endangered | 1 | yes | yes | yes | D |
| Unknown white (from Villadeati) | b | - | Muscat type | Piedmont (Monferrato) | Endangered | 1 | no | no | no | D |
| Usteng | n | H | None | Piedmont (west) | Endangered | 1 | yes | no | no | D |
| Uva 'd Batita | n | H | None | Piedmont (Monferrato) | Endangered | 1 | yes | yes | no | D |

| | | | | | | | | | | |
|---|---|---|---|---|---|---|---|---|---|---|
| Uva 'd Piasa | n | H | None | Piedmont (north), Aosta Valley | Endangered | 2 | no | no | no | D |
| Uva delle cascine | n | H | None | Oltrepò pavese | Endangered | 2 | yes | yes | no | D |
| Uva rara N. | n | H | None | Piedmont (north), Oltrepò pavese | Minor | 1 | yes | yes | yes | D |
| Uvalino N. | n | H | None | Piedmont (Monferrato) | Local | 1 | yes | yes | yes | D |
| Uvetta (from Carpignano S.) | n | H | None | Piedmont (north) | Endangered | 1 | no | no | no | - |
| Verdea B. | b | H | None | Piedmont (south-east), Liguria | Local | 7 | yes | yes | no | - |
| Vermentino B. | b | H | None | Liguria, Piedmont | Major | 7 | yes | yes | yes | D |
| Vermentino nero N. | n | H | None | Tuscany | Local | 1 | yes | yes | no | - |
| *Vernaccia di S. Gimignano B.* | b | H | None | Tuscany, Liguria | Major | 5 | yes | yes | no | D |
| Vespolina N. | n | H | None | Piedmont (north), Oltrepò pavese | Major | 5 | yes | yes | yes | D |
| Vien de Nus N. | n | H | None | Aosta valley | Local | 2 | yes | yes | yes | D |
| Viognier B. | b | H | None | France, International | Major | 1 | yes | yes | yes | D |
| Vurpin (false) | n | H | None | Piedmont (west) | Endangered | 1 | yes | yes | no | D |
| Vurpin (presumed) | n | H | None | Piedmont (west) | Endangered | 1 | yes | no | no | - |
| Zanello | n | H | None | Piedmont (Monferrato) | Endangered | 3 | yes | yes | yes | D |
| Zibibbo B. (Muscat of Alexandria) | b | H | Muscat type | Mediterranean sea coastal areas | Major | 9 | yes | yes | yes | B |

Table S2 – Genetic parameters obtained from nSSR markers (32 loci) on the examined grapevine panel (227 unique cultivars).

| | | | | | Average non exclusion probabilities for | | | | | | | |
|---|---|---|---|---|---|---|---|---|---|---|---|
| **Locus** | **N° of alleles** | **HObs** | **HExp** | **PIC** | **one candidate parent** | **one candidate parent given the other parent** | **a candidate parent pair** | **identity of two unrelated individuals** | **identity of two siblings** | **Hardy-Weinberg disequilibrium** | **Null allele frequency** |
| VvMD5 | 8 | 0.890 | 0.828 | 0.805 | 0.514 | 0.341 | 0.163 | 0.052 | 0.350 | NS | -0.0400 |
| VvMD7 | 12 | 0.819 | 0.798 | 0.772 | 0.559 | 0.382 | 0.193 | 0.066 | 0.369 | NS | -0.0144 |
| VvMD25 | 11 | 0.824 | 0.777 | 0.738 | 0.621 | 0.443 | 0.262 | 0.088 | 0.384 | NS | -0.0321 |
| VvMD27 | 8 | 0.793 | 0.794 | 0.761 | 0.587 | 0.409 | 0.227 | 0.075 | 0.373 | NS | 0.0006 |
| VvMD28 | 13 | 0.921 | 0.876 | 0.861 | 0.407 | 0.254 | 0.098 | 0.029 | 0.320 | NS | -0.0261 |
| VvMD32 | 10 | 0.850 | 0.799 | 0.772 | 0.564 | 0.386 | 0.199 | 0.066 | 0.368 | NS | -0.0309 |
| VvS2 | 12 | 0.872 | 0.833 | 0.811 | 0.503 | 0.331 | 0.155 | 0.049 | 0.347 | NS | -0.0254 |
| VrZAG62 | 10 | 0.863 | 0.838 | 0.815 | 0.499 | 0.328 | 0.155 | 0.048 | 0.344 | NS | -0.0155 |
| VrZAG79 | 12 | 0.859 | 0.815 | 0.794 | 0.524 | 0.349 | 0.162 | 0.054 | 0.357 | NS | -0.0301 |
| VvMD36 | 17 | 0.714 | 0.810 | 0.788 | 0.531 | 0.357 | 0.168 | 0.057 | 0.360 | NS | 0.0570 |
| VrZAG67 | 10 | 0.837 | 0.810 | 0.783 | 0.551 | 0.375 | 0.194 | 0.063 | 0.361 | NS | -0.0168 |
| VrZAG64 | 11 | 0.881 | 0.837 | 0.815 | 0.496 | 0.326 | 0.151 | 0.047 | 0.344 | NS | -0.0265 |
| VvMD31 | 8 | 0.665 | 0.678 | 0.626 | 0.732 | 0.564 | 0.378 | 0.156 | 0.451 | NS | 0.0112 |
| VvMD24 | 6 | 0.758 | 0.696 | 0.636 | 0.734 | 0.570 | 0.400 | 0.152 | 0.441 | NS | -0.0441 |
| VRZAG21 | 9 | 0.828 | 0.745 | 0.703 | 0.660 | 0.483 | 0.297 | 0.107 | 0.405 | NS | -0.0585 |
| VvMD6 | 6 | 0.767 | 0.685 | 0.626 | 0.740 | 0.577 | 0.404 | 0.158 | 0.448 | NS | -0.0596 |
| VVMD21 | 5 | 0.595 | 0.557 | 0.514 | 0.833 | 0.671 | 0.495 | 0.239 | 0.532 | NS | -0.0345 |
| VMC5A1 | 8 | 0.762 | 0.733 | 0.688 | 0.675 | 0.501 | 0.316 | 0.116 | 0.413 | NS | -0.0246 |
| VMC2H4 | 14 | 0.775 | 0.749 | 0.720 | 0.631 | 0.448 | 0.247 | 0.091 | 0.399 | NS | -0.0212 |
| VMC1C10 | 12 | 0.784 | 0.776 | 0.746 | 0.594 | 0.415 | 0.221 | 0.079 | 0.383 | NS | -0.0054 |
| VVIB01 | 6 | 0.793 | 0.706 | 0.651 | 0.719 | 0.550 | 0.375 | 0.141 | 0.433 | NS | -0.0602 |
| VMC5G8 | 8 | 0.775 | 0.708 | 0.660 | 0.706 | 0.532 | 0.348 | 0.133 | 0.430 | NS | -0.0541 |
| VMC1E8 | 10 | 0.868 | 0.843 | 0.821 | 0.487 | 0.318 | 0.147 | 0.045 | 0.341 | NS | -0.0155 |
| VMC4C6 | 8 | 0.740 | 0.678 | 0.618 | 0.744 | 0.583 | 0.410 | 0.164 | 0.452 | NS | -0.0457 |
| VVMD34 | 5 | 0.304 | 0.286 | 0.271 | 0.958 | 0.846 | 0.730 | 0.525 | 0.738 | NS | -0.0415 |
| VMC3D12 | 11 | 0.731 | 0.702 | 0.665 | 0.697 | 0.515 | 0.316 | 0.125 | 0.431 | NS | -0.0194 |
| VvMD26 | 6 | 0.727 | 0.669 | 0.615 | 0.748 | 0.581 | 0.401 | 0.163 | 0.457 | NS | -0.0444 |
| VMC2F10 | 10 | 0.789 | 0.735 | 0.701 | 0.658 | 0.475 | 0.279 | 0.104 | 0.409 | NS | -0.0361 |
| VMC7F2 | 5 | 0.626 | 0.565 | 0.530 | 0.822 | 0.650 | 0.463 | 0.224 | 0.524 | NS | -0.0610 |
| VVIQ52 | 7 | 0.740 | 0.656 | 0.585 | 0.775 | 0.625 | 0.465 | 0.189 | 0.470 | NS | -0.0638 |
| VvIN73 | 5 | 0.295 | 0.301 | 0.286 | 0.953 | 0.836 | 0.712 | 0.504 | 0.726 | NS | 0.0182 |
| VVP31 | 12 | 0.934 | 0.878 | 0.864 | 0.400 | 0.249 | 0.094 | 0.028 | 0.319 | NS | -0.0324 |
| Average | 9.219 | 0.762 | 0.724 | 0.689 | | | | | | | |
| Cumulative | | | | | 3.07E-07 | 1.18E-08 | 3.20E-16 | 6.41E-30 | 4.90E-10 | | |

Table S3 - Allelic size (in bp) of the 227 grapevine cultivars from the core sample set analysed at 32 nSSR loci. Outgroup cultivars are indicated in italic, reference varieties are in bold.

| Cultivar name | VvMD5 | | VvMD7 | | VvMD25 | | VvMD27 | | VvMD28 | | VvMD32 | | VvS2 | | VrZAG62 | | VrZAG79 | | VvMD36 | | VrZAG67 | | VrZAG64 | | VvMD31 | | VvMD24 | | VRZAG21 | | VvMD6 | | VVMD21 | | VMC5A1 | | VMC2H4 | | VMC1C10 | | VVIB01 | | VMC5G8 | | VMC1E8 | | VMC4C6 | | VVMD34 | | VMC3D12 | | VvMD26 | | VMC2F10 | | VMC7F2 | | VVIQ52 | | VvIN73 | | VVP31 | |
|---|---|---|---|---|---|---|---|---|---|---|---|---|---|---|---|---|---|---|---|---|---|---|---|---|---|---|---|---|---|---|---|---|---|---|---|---|---|---|---|---|---|---|---|---|---|---|---|---|---|---|---|---|---|---|---|---|---|---|---|---|---|---|---|---|
| *Albana B.* | 225 | 231 | 247 | 247 | 243 | 257 | 178 | 188 | 236 | 238 | 251 | 273 | 133 | 143 | 194 | 200 | 250 | 250 | 247 | 253 | 150 | 153 | 137 | 139 | 203 | 209 | 209 | 209 | 203 | 207 | 208 | 210 | 248 | 254 | 160 | 170 | 209 | 217 | 149 | 153 | 289 | 295 | 310 | 316 | 208 | 222 | 159 | 169 | 237 | 237 | 212 | 218 | 253 | 261 | 90 | 94 | 201 | 201 | 79 | 85 | 263 | 265 | 176 | 184 |
| Albanina nera | 231 | 239 | 239 | 253 | 243 | 257 | 184 | 184 | 230 | 262 | 253 | 273 | 133 | 143 | 192 | 194 | 244 | 250 | 263 | 265 | 150 | 153 | 137 | 139 | 209 | 211 | 213 | 215 | 191 | 203 | 208 | 210 | 248 | 248 | 170 | 170 | 217 | 217 | 153 | 153 | 291 | 295 | 302 | 316 | 208 | 218 | 159 | 165 | 237 | 237 | 200 | 206 | 253 | 261 | 94 | 100 | 201 | 205 | 85 | 85 | 263 | 263 | 174 | 184 |
| *Albarola B.* | 231 | 235 | 243 | 263 | 241 | 243 | 178 | 180 | 236 | 246 | 253 | 263 | 133 | 155 | 188 | 194 | 248 | 258 | 251 | 296 | 126 | 139 | 139 | 159 | 195 | 211 | 209 | 217 | 201 | 203 | 210 | 210 | 242 | 254 | 160 | 166 | 209 | 217 | 153 | 153 | 291 | 299 | 312 | 316 | 208 | 222 | 159 | 165 | 237 | 239 | 200 | 206 | 247 | 247 | 90 | 100 | 201 | 205 | 79 | 81 | 263 | 263 | 192 | 196 |
| Aleatico (false, from Paderna) | 231 | 235 | 233 | 247 | 251 | 257 | 178 | 178 | 238 | 270 | 251 | 265 | 133 | 139 | 196 | 204 | 254 | 258 | 263 | 271 | 126 | 153 | 137 | 141 | 211 | 215 | 213 | 213 | 203 | 207 | 208 | 210 | 248 | 248 | 166 | 172 | 217 | 217 | 139 | 153 | 291 | 295 | 312 | 312 | 228 | 230 | 159 | 169 | 237 | 237 | 206 | 222 | 247 | 249 | 94 | 104 | 199 | 201 | 79 | 81 | 263 | 263 | 180 | 184 |
| Aleatico N. | 225 | 227 | 239 | 249 | 251 | 257 | 178 | 193 | 238 | 248 | 265 | 273 | 133 | 135 | 186 | 196 | 248 | 254 | 243 | 253 | 139 | 155 | 139 | 159 | 211 | 215 | 209 | 213 | 193 | 207 | 190 | 210 | 242 | 264 | 160 | 160 | 203 | 217 | 139 | 153 | 289 | 295 | 312 | 316 | 208 | 228 | 159 | 165 | 237 | 237 | 200 | 206 | 247 | 249 | 94 | 100 | 199 | 201 | 79 | 85 | 261 | 263 | 184 | 184 |
| Arneis B. | 225 | 235 | 247 | 249 | 241 | 257 | 188 | 188 | 246 | 260 | 241 | 273 | 135 | 155 | 196 | 204 | 244 | 250 | 251 | 253 | 126 | 155 | 141 | 163 | 215 | 215 | 209 | 217 | 191 | 207 | 208 | 210 | 248 | 254 | 166 | 170 | 209 | 217 | 153 | 181 | 291 | 295 | 316 | 316 | 218 | 230 | 159 | 159 | 237 | 237 | 206 | 222 | 247 | 247 | 94 | 104 | 201 | 201 | 81 | 85 | 263 | 263 | 180 | 190 |
| Avanà N. | 225 | 225 | 247 | 247 | 241 | 259 | 184 | 184 | 246 | 246 | 263 | 273 | 133 | 155 | 194 | 194 | 238 | 242 | 251 | 271 | 139 | 150 | 143 | 159 | 209 | 215 | 213 | 217 | 191 | 207 | 208 | 210 | 248 | 254 | 170 | 170 | 201 | 217 | 153 | 165 | 291 | 299 | 310 | 316 | 208 | 218 | 159 | 165 | 237 | 237 | 206 | 222 | 247 | 249 | 90 | 100 | 201 | 201 | 79 | 85 | 263 | 263 | 180 | 192 |
| Avarenchetto | 225 | 239 | 249 | 249 | 241 | 257 | 178 | 193 | 230 | 246 | 241 | 251 | 133 | 135 | 204 | 204 | 236 | 250 | 251 | 275 | 139 | 155 | 159 | 163 | 213 | 215 | 209 | 217 | 191 | 203 | 210 | 210 | 248 | 248 | 160 | 170 | 201 | 223 | 153 | 165 | 291 | 295 | 302 | 316 | 208 | 228 | 159 | 165 | 237 | 237 | 200 | 222 | 247 | 253 | 100 | 104 | 201 | 205 | 79 | 85 | 263 | 263 | 176 | 190 |
| Avarengo N. | 225 | 231 | 247 | 247 | 243 | 243 | 178 | 184 | 238 | 260 | 241 | 253 | 139 | 155 | 194 | 200 | 248 | 258 | 263 | 263 | 139 | 150 | 159 | 163 | 195 | 211 | 213 | 213 | 191 | 203 | 208 | 210 | 248 | 248 | 166 | 170 | 201 | 209 | 153 | 165 | 295 | 299 | 302 | 302 | 230 | 230 | 169 | 169 | 237 | 237 | 200 | 206 | 247 | 247 | 94 | 104 | 201 | 201 | 81 | 85 | 263 | 263 | 180 | 184 |
| Balsamina (from Reggio Emilia area) | 225 | 231 | 247 | 247 | 257 | 259 | 184 | 188 | 236 | 260 | 241 | 253 | 143 | 155 | 194 | 196 | 244 | 250 | 253 | 253 | 150 | 150 | 139 | 143 | 211 | 211 | 213 | 217 | 191 | 201 | 190 | 210 | 248 | 248 | 166 | 170 | 209 | 217 | 153 | 163 | 295 | 295 | 302 | 316 | 206 | 230 | 165 | 165 | 237 | 237 | 206 | 222 | 249 | 253 | 90 | 94 | 201 | 205 | 79 | 85 | 263 | 263 | 174 | 184 |
| Baratuciat B. | 225 | 227 | 233 | 253 | 257 | 269 | 178 | 188 | 220 | 236 | 241 | 273 | 139 | 151 | 196 | 200 | 246 | 258 | 294 | 294 | 126 | 139 | 141 | 159 | 195 | 211 | 209 | 213 | 201 | 203 | 210 | 210 | 242 | 264 | 166 | 170 | 201 | 217 | 153 | 159 | 289 | 291 | 312 | 316 | 222 | 222 | 165 | 165 | 237 | 237 | 206 | 206 | 247 | 249 | 94 | 100 | 201 | 201 | 79 | 85 | 263 | 263 | 190 | 194 |
| Barbarossa di Piemonte | 227 | 239 | 239 | 247 | 241 | 243 | 180 | 184 | 262 | 270 | 241 | 253 | 133 | 145 | 194 | 196 | 250 | 258 | 263 | 265 | 126 | 132 | 137 | 141 | 211 | 215 | 209 | 209 | 203 | 203 | 190 | 210 | 248 | 254 | 166 | 170 | 209 | 209 | 131 | 165 | 289 | 295 | 302 | 316 | 208 | 218 | 165 | 177 | 237 | 237 | 200 | 212 | 247 | 249 | 90 | 104 | 201 | 203 | 79 | 79 | 263 | 263 | 178 | 194 |
| Barbassese | 225 | 227 | 247 | 249 | 243 | 251 | 178 | 188 | 236 | 260 | 257 | 265 | 133 | 133 | 196 | 196 | 244 | 258 | 263 | 263 | 153 | 153 | 137 | 163 | 215 | 215 | 209 | 209 | 191 | 207 | 196 | 210 | 248 | 248 | 160 | 170 | 209 | 217 | 139 | 165 | 289 | 291 | 302 | 316 | 218 | 228 | 165 | 165 | 237 | 237 | 206 | 218 | 247 | 249 | 94 | 94 | 199 | 201 | 79 | 81 | 263 | 263 | 180 | 194 |
| Barbera bianca B. | 225 | 239 | 247 | 253 | 243 | 251 | 178 | 180 | 260 | 270 | 253 | 273 | 133 | 143 | 200 | 204 | 250 | 260 | 263 | 263 | 139 | 153 | 159 | 163 | 211 | 215 | 209 | 213 | 207 | 215 | 208 | 210 | 248 | 248 | 164 | 170 | 207 | 209 | 139 | 165 | 289 | 295 | 302 | 312 | 210 | 222 | 165 | 165 | 237 | 239 | 206 | 218 | 247 | 249 | 94 | 100 | 201 | 203 | 85 | 85 | 263 | 263 | 186 | 194 |
| Barbera ciarìa | 225 | 227 | 247 | 247 | 243 | 257 | 184 | 188 | 238 | 266 | 241 | 273 | 135 | 155 | 196 | 200 | 250 | 258 | 269 | 269 | 139 | 155 | 159 | 163 | 195 | 211 | 213 | 217 | 191 | 207 | 194 | 208 | 248 | 248 | 168 | 170 | 201 | 217 | 153 | 165 | 291 | 299 | 302 | 316 | 230 | 232 | 159 | 169 | 237 | 237 | 206 | 222 | 247 | 253 | 94 | 130 | 199 | 201 | 79 | 81 | 263 | 263 | 184 | 192 |
| Barbera di Patrunat | 227 | 235 | 247 | 249 | 243 | 243 | 184 | 193 | 236 | 248 | 253 | 265 | 133 | 133 | 186 | 194 | 250 | 258 | 243 | 243 | 139 | 139 | 159 | 159 | 211 | 211 | 217 | 217 | 191 | 207 | 190 | 210 | 248 | 248 | 160 | 166 | 203 | 217 | 139 | 159 | 291 | 291 | 312 | 316 | 228 | 230 | 159 | 165 | 237 | 237 | 206 | 206 | 247 | 249 | 90 | 94 | 199 | 201 | 79 | 79 | 261 | 263 | 184 | 190 |
| Barbera N. | 225 | 225 | 249 | 253 | 241 | 257 | 184 | 188 | 236 | 262 | 253 | 273 | 133 | 135 | 192 | 200 | 242 | 258 | 265 | 265 | 132 | 139 | 159 | 163 | 211 | 211 | 209 | 217 | 191 | 201 | 190 | 208 | 242 | 248 | 170 | 170 | 209 | 227 | 165 | 181 | 289 | 295 | 302 | 304 | 206 | 208 | 159 | 165 | 237 | 237 | 222 | 222 | 247 | 247 | 94 | 104 | 201 | 201 | 81 | 85 | 263 | 263 | 192 | 194 |
| Barberùn | 225 | 227 | 247 | 247 | 251 | 257 | 184 | 184 | 246 | 260 | 273 | 273 | 143 | 155 | 204 | 204 | 250 | 250 | 253 | 269 | 139 | 153 | 137 | 159 | 211 | 215 | 209 | 217 | 203 | 207 | 190 | 210 | 248 | 254 | 166 | 166 | 209 | 209 | 153 | 165 | 295 | 295 | 302 | 316 | 228 | 232 | 159 | 165 | 237 | 237 | 206 | 222 | 253 | 253 | 94 | 94 | 201 | 203 | 79 | 85 | 263 | 263 | 180 | 190 |
| Barbrassa | 225 | 235 | 239 | 243 | 243 | 257 | 178 | 188 | 236 | 238 | 273 | 273 | 143 | 145 | 186 | 188 | 244 | 254 | 247 | 263 | 139 | 139 | 153 | 159 | 203 | 211 | 209 | 217 | 191 | 203 | 208 | 208 | 248 | 254 | 166 | 170 | 217 | 227 | 131 | 153 | 295 | 295 | 302 | 312 | 228 | 232 | 159 | 165 | 237 | 237 | 206 | 206 | 247 | 253 | 94 | 100 | 201 | 201 | 81 | 85 | 263 | 263 | 180 | 194 |
| Berla grossa | 235 | 239 | 239 | 261 | 243 | 257 | 180 | 193 | 248 | 260 | 251 | 253 | 139 | 143 | 194 | 196 | 242 | 250 | 253 | 263 | 139 | 139 | 159 | 159 | 195 | 211 | 209 | 217 | 203 | 207 | 190 | 208 | 248 | 248 | 160 | 166 | 203 | 203 | 153 | 165 | 295 | 299 | 302 | 310 | 222 | 232 | 159 | 165 | 237 | 239 | 206 | 206 | 247 | 249 | 98 | 104 | 201 | 205 | 85 | 85 | 263 | 263 | 176 | 184 |
| *Besgano bianco* | 225 | 227 | 233 | 247 | 241 | 257 | 178 | 193 | 236 | 260 | 251 | 273 | 133 | 135 | 194 | 204 | 236 | 250 | 251 | 253 | 126 | 155 | 139 | 193 | 209 | 211 | 209 | 217 | 203 | 207 | 208 | 210 | 248 | 254 | 166 | 170 | 201 | 203 | 153 | 165 | 295 | 299 | 302 | 312 | 208 | 218 | 159 | 159 | 237 | 239 | 206 | 206 | 247 | 247 | 94 | 100 | 201 | 205 | 81 | 85 | 263 | 263 | 184 | 192 |
| Bianchetta (from Frascata) | 225 | 233 | 247 | 253 | 241 | 257 | 180 | 182 | 236 | 236 | 241 | 273 | 139 | 143 | 200 | 204 | 246 | 258 | 263 | 275 | 139 | 153 | 137 | 153 | 211 | 215 | 209 | 217 | 191 | 207 | 208 | 210 | 242 | 248 | 160 | 170 | 217 | 217 | 153 | 165 | 291 | 299 | 312 | 316 | 226 | 230 | 153 | 165 | 237 | 237 | 218 | 224 | 247 | 249 | 94 | 94 | 201 | 201 | 79 | 85 | 263 | 263 | 180 | 180 |
| Bianchetto (from Saluzzo) | 225 | 237 | 247 | 249 | 243 | 259 | 184 | 184 | 236 | 246 | 273 | 273 | 133 | 155 | 194 | 200 | 242 | 246 | 251 | 294 | 139 | 153 | 137 | 159 | 195 | 215 | 209 | 213 | 207 | 207 | 208 | 210 | 248 | 254 | 156 | 170 | 201 | 217 | 153 | 153 | 291 | 295 | 310 | 316 | 208 | 222 | 159 | 167 | 237 | 237 | 206 | 222 | 247 | 249 | 90 | 90 | 201 | 203 | 79 | 85 | 255 | 263 | 188 | 192 |
| Bibiola | 225 | 231 | 247 | 249 | 241 | 251 | 178 | 188 | 230 | 246 | 253 | 263 | 133 | 151 | 186 | 200 | 244 | 258 | 251 | 263 | 150 | 153 | 137 | 163 | 211 | 215 | 209 | 209 | 203 | 207 | 208 | 210 | 248 | 254 | 166 | 170 | 203 | 203 | 163 | 165 | 289 | 295 | 302 | 312 | 218 | 230 | 169 | 177 | 237 | 237 | 218 | 222 | 249 | 261 | 90 | 100 | 199 | 201 | 79 | 81 | 263 | 263 | 188 | 194 |
| Biestro (presumed) | 231 | 233 | 247 | 255 | 251 | 257 | 178 | 182 | 236 | 236 | 273 | 273 | 143 | 151 | 204 | 204 | 246 | 258 | 263 | 263 | 139 | 150 | 159 | 163 | 215 | 215 | 209 | 217 | 207 | 207 | 210 | 210 | 248 | 254 | 160 | 166 | 201 | 217 | 165 | 165 | 295 | 299 | 302 | 316 | 218 | 230 | 165 | 169 | 237 | 237 | 206 | 222 | 249 | 261 | 90 | 94 | 201 | 203 | 79 | 83 | 263 | 263 | 176 | 180 |
| Blanchet | 231 | 235 | 247 | 247 | 241 | 251 | 178 | 184 | 236 | 246 | 263 | 273 | 139 | 151 | 194 | 204 | 250 | 258 | 263 | 263 | 139 | 153 | 137 | 159 | 211 | 211 | 213 | 213 | 201 | 201 | 208 | 208 | 248 | 254 | 160 | 166 | 203 | 217 | 153 | 165 | 291 | 295 | 312 | 316 | 230 | 230 | 165 | 169 | 237 | 237 | 206 | 222 | 247 | 249 | 90 | 94 | 201 | 201 | 79 | 81 | 263 | 263 | 180 | 188 |
| Bonarda N. | 245 | 245 | 249 | 255 | 241 | 243 | 184 | 193 | 230 | 238 | 273 | 273 | 143 | 155 | 200 | 204 | 238 | 250 | 251 | 253 | 132 | 150 | 163 | 163 | 203 | 209 | 213 | 217 | 207 | 207 | 190 | 208 | 242 | 254 | 164 | 170 | 203 | 217 | 153 | 165 | 289 | 291 | 302 | 316 | 230 | 230 | 159 | 165 | 237 | 237 | 200 | 206 | 247 | 261 | 90 | 104 | 201 | 201 | 79 | 79 | 255 | 263 | 180 | 188 |
| Bonardina | 227 | 235 | 233 | 249 | 251 | 251 | 184 | 188 | 238 | 248 | 253 | 265 | 133 | 151 | 196 | 196 | 242 | 244 | 243 | 243 | 139 | 153 | 137 | 159 | 215 | 215 | 209 | 217 | 191 | 207 | 208 | 210 | 248 | 248 | 166 | 170 | 201 | 217 | 165 | 165 | 291 | 299 | 312 | 316 | 218 | 228 | 159 | 165 | 237 | 237 | 196 | 206 | 249 | 261 | 94 | 104 | 201 | 201 | 79 | 79 | 261 | 263 | 184 | 190 |
| Bonda N. | 225 | 231 | 247 | 247 | 251 | 257 | 184 | 193 | 236 | 270 | 273 | 273 | 151 | 151 | 186 | 204 | 250 | 250 | 251 | 263 | 139 | 139 | 159 | 159 | 203 | 215 | 209 | 209 | 193 | 207 | 208 | 210 | 242 | 254 | 166 | 170 | 203 | 217 | 165 | 165 | 291 | 299 | 312 | 316 | 208 | 208 | 165 | 165 | 237 | 237 | 206 | 218 | 249 | 251 | 90 | 94 | 201 | 205 | 79 | 81 | 255 | 263 | 176 | 184 |
| Bondola | 225 | 237 | 239 | 239 | 243 | 259 | 188 | 188 | 220 | 270 | 251 | 263 | 133 | 137 | 188 | 194 | 246 | 250 | 253 | 263 | 153 | 155 | 163 | 193 | 211 | 215 | 209 | 213 | 201 | 203 | 200 | 208 | 248 | 254 | 156 | 170 | 217 | 217 | 131 | 153 | 291 | 291 | 312 | 316 | 222 | 230 | 165 | 169 | 237 | 237 | 200 | 204 | 247 | 247 | 90 | 108 | 201 | 203 | 79 | 85 | 263 | 263 | 192 | 196 |
| Bordò | 225 | 227 | 247 | 249 | 241 | 243 | 178 | 184 | 260 | 270 | 241 | 265 | 133 | 155 | 186 | 196 | 242 | 250 | 253 | 263 | 126 | 139 | 141 | 159 | 215 | 215 | 209 | 213 | 191 | 207 | 208 | 210 | 248 | 264 | 160 | 170 | 201 | 203 | 139 | 153 | 291 | 299 | 312 | 316 | 218 | 228 | 159 | 169 | 237 | 237 | 206 | 206 | 247 | 249 | 100 | 104 | 199 | 201 | 79 | 85 | 261 | 263 | 184 | 192 |
| *Bosco B.* | 225 | 235 | 239 | 263 | 251 | 257 | 180 | 184 | 246 | 260 | 257 | 273 | 133 | 135 | 188 | 196 | 250 | 258 | 263 | 269 | 139 | 150 | 143 | 159 | 211 | 211 | 209 | 213 | 191 | 207 | 190 | 208 | 242 | 248 | 170 | 174 | 217 | 217 | 153 | 163 | 291 | 295 | 316 | 316 | 230 | 230 | 153 | 169 | 237 | 245 | 200 | 222 | 249 | 261 | 94 | 100 | 201 | 205 | 79 | 85 | 263 | 263 | 184 | 190 |
| Bottagera (false) | 225 | 235 | 247 | 249 | 257 | 257 | 184 | 193 | 246 | 260 | 241 | 273 | 135 | 143 | 196 | 204 | 242 | 250 | 251 | 253 | 139 | 155 | 159 | 163 | 211 | 215 | 217 | 217 | 191 | 207 | 208 | 210 | 248 | 254 | 166 | 170 | 201 | 209 | 153 | 181 | 291 | 295 | 304 | 316 | 228 | 232 | 159 | 159 | 237 | 237 | 206 | 222 | 247 | 253 | 94 | 104 | 201 | 201 | 79 | 85 | 263 | 263 | 190 | 192 |
| Bracciola nera N. | 233 | 239 | 253 | 253 | 243 | 257 | 184 | 193 | 238 | 248 | 251 | 259 | 133 | 145 | 194 | 200 | 250 | 250 | 263 | 265 | 139 | 139 | 139 | 159 | 209 | 211 | 209 | 213 | 201 | 203 | 200 | 208 | 248 | 248 | 170 | 170 | 203 | 209 | 149 | 181 | 291 | 291 | 302 | 316 | 218 | 228 | 159 | 169 | 221 | 237 | 200 | 200 | 247 | 247 | 98 | 100 | 201 | 205 | 79 | 85 | 263 | 265 | 184 | 184 |
| Brachetto (false, from Nizza M.to) | 231 | 235 | 247 | 249 | 251 | 251 | 178 | 178 | 236 | 248 | 251 | 273 | 133 | 139 | 186 | 204 | 248 | 254 | 243 | 263 | 126 | 153 | 137 | 141 | 211 | 215 | 217 | 217 | 203 | 207 | 210 | 210 | 254 | 264 | 166 | 172 | 217 | 217 | 139 | 153 | 291 | 291 | 302 | 316 | 230 | 232 | 159 | 169 | 237 | 237 | 206 | 222 | 247 | 249 | 100 | 108 | 199 | 201 | 79 | 81 | 263 | 263 | 188 | 194 |
| Brachetto Migliardi | 225 | 227 | 233 | 255 | 251 | 257 | 178 | 193 | 260 | 270 | 241 | 273 | 133 | 143 | 196 | 204 | 250 | 254 | 253 | 263 | 139 | 139 | 159 | 159 | 211 | 215 | 213 | 217 | 191 | 207 | 190 | 208 | 248 | 248 | 160 | 160 | 217 | 217 | 139 | 165 | 291 | 295 | 316 | 316 | 228 | 230 | 159 | 165 | 237 | 245 | 206 | 206 | 247 | 247 | 100 | 104 | 201 | 203 | 79 | 79 | 261 | 263 | 180 | 190 |
| Brachetto N. | 227 | 235 | 233 | 247 | 243 | 243 | 178 | 178 | 248 | 260 | 241 | 265 | 133 | 139 | 194 | 196 | 248 | 254 | 243 | 263 | 126 | 150 | 141 | 163 | 211 | 211 | 213 | 217 | 207 | 207 | 208 | 210 | 248 | 248 | 160 | 166 | 201 | 217 | 165 | 181 | 295 | 299 | 312 | 316 | 228 | 230 | 159 | 159 | 237 | 237 | 206 | 206 | 247 | 249 | 94 | 104 | 199 | 201 | 79 | 79 | 263 | 263 | 188 | 190 |
| Bragat rosa N. | 227 | 231 | 247 | 253 | 251 | 257 | 178 | 182 | 236 | 246 | 263 | 265 | 143 | 151 | 188 | 204 | 250 | 254 | 263 | 263 | 126 | 153 | 137 | 139 | 211 | 215 | 209 | 217 | 203 | 207 | 208 | 210 | 248 | 254 | 160 | 160 | 201 | 217 | 165 | 165 | 291 | 299 | 312 | 316 | 208 | 230 | 159 | 169 | 237 | 237 | 206 | 218 | 247 | 249 | 94 | 100 | 201 | 201 | 79 | 79 | 263 | 263 | 188 | 194 |
| Bressana | 231 | 235 | 247 | 247 | 243 | 257 | 188 | 193 | 260 | 270 | 257 | 273 | 135 | 155 | 194 | 196 | 250 | 250 | 253 | 263 | 155 | 163 | 149 | 163 | 211 | 215 | 209 | 217 | 207 | 207 | 208 | 210 | 248 | 254 | 166 | 170 | 209 | 217 | 131 | 181 | 295 | 299 | 280 | 312 | 222 | 232 | 159 | 165 | 237 | 239 | 200 | 222 | 247 | 253 | 94 | 94 | 201 | 201 | 81 | 85 | 263 | 263 | 190 | 192 |
| Brugnola | 231 | 235 | 247 | 253 | 243 | 243 | 182 | 188 | 260 | 270 | 241 | 263 | 145 | 155 | 194 | 194 | 250 | 250 | 263 | 263 | 126 | 153 | 141 | 163 | 211 | 215 | 211 | 213 | 191 | 215 | 208 | 210 | 248 | 254 | 164 | 166 | 217 | 217 | 153 | 159 | 291 | 299 | 302 | 312 | 208 | 218 | 159 | 165 | 237 | 239 | 200 | 206 | 247 | 249 | 104 | 104 | 201 | 201 | 85 | 85 | 263 | 263 | 180 | 194 |
| Brunetta (from Rivoli) | 225 | 231 | 247 | 263 | 241 | 257 | 184 | 184 | 260 | 262 | 241 | 265 | 133 | 135 | 196 | 202 | 238 | 242 | 253 | 265 | 139 | 155 | 139 | 159 | 209 | 211 | 211 | 213 | 191 | 201 | 208 | 208 | 248 | 248 | 160 | 166 | 209 | 227 | 153 | 153 | 291 | 295 | 302 | 316 | 208 | 228 | 159 | 159 | 237 | 245 | 200 | 212 | 247 | 247 | 94 | 94 | 201 | 205 | 81 | 81 | 263 | 263 | 190 | 192 |
| Bubbierasco | 225 | 235 | 249 | 249 | 243 | 259 | 184 | 188 | 246 | 270 | 263 | 273 | 155 | 155 | 200 | 200 | 242 | 250 | 251 | 263 | 139 | 163 | 149 | 159 | 195 | 211 | 209 | 213 | 191 | 207 | 208 | 208 | 248 | 254 | 166 | 170 | 217 | 217 | 153 | 153 | 291 | 295 | 302 | 310 | 208 | 222 | 159 | 167 | 237 | 237 | 198 | 206 | 247 | 249 | 90 | 104 | 201 | 203 | 79 | 85 | 255 | 263 | 192 | 196 |
| **Cabernet franc N.** | 225 | 239 | 239 | 263 | 241 | 257 | 180 | 188 | 230 | 238 | 241 | 259 | 139 | 147 | 194 | 204 | 246 | 258 | 253 | 253 | 139 | 139 | 159 | 159 | 205 | 215 | 209 | 209 | 191 | 201 | 200 | 208 | 248 | 256 | 168 | 170 | 215 | 217 | 153 | 153 | 291 | 291 | 316 | 320 | 222 | 228 | 165 | 165 | 237 | 237 | 196 | 222 | 247 | 247 | 94 | 104 | 201 | 201 | 79 | 79 | 263 | 267 | 190 | 190 |
| **Cabernet sauvignon N.** | 231 | 239 | 239 | 239 | 241 | 251 | 174 | 188 | 236 | 238 | 241 | 241 | 139 | 151 | 188 | 194 | 246 | 246 | 253 | 263 | 126 | 139 | 139 | 159 | 205 | 209 | 209 | 217 | 201 | 207 | 208 | 208 | 248 | 256 | 166 | 168 | 215 | 223 | 153 | 165 | 291 | 291 | 312 | 320 | 222 | 222 | 165 | 177 | 237 | 245 | 200 | 222 | 247 | 249 | 94 | 104 | 199 | 201 | 79 | 85 | 263 | 267 | 190 | 190 |
| Cagambraga | 225 | 245 | 249 | 251 | 241 | 251 | 180 | 188 | 262 | 270 | 273 | 273 | 139 | 155 | 196 | 200 | 244 | 258 | 263 | 265 | 153 | 153 | 137 | 137 | 211 | 211 | 209 | 213 | 191 | 207 | 190 | 208 | 242 | 248 | 160 | 170 | 209 | 217 | 165 | 181 | 289 | 295 | 302 | 302 | 218 | 230 | 165 | 165 | 237 | 237 | 200 | 206 | 247 | 247 | 94 | 94 | 201 | 203 | 79 | 81 | 263 | 263 | 180 | 194 |
| Calora (presumed) | 225 | 225 | 239 | 247 | 241 | 241 | 184 | 188 | 260 | 270 | 253 | 273 | 133 | 139 | 186 | 186 | 238 | 244 | 263 | 275 | 150 | 150 | 143 | 163 | 203 | 215 | 209 | 217 | 203 | 207 | 208 | 210 | 242 | 242 | 170 | 170 | 209 | 217 | 139 | 153 | 291 | 295 | 312 | 316 | 218 | 218 | 165 | 169 | 237 | 245 | 206 | 212 | 247 | 247 | 94 | 94 | 199 | 201 | 85 | 85 | 261 | 263 | 190 | 194 |
| Calora bianca (presumed) | 225 | 231 | 247 | 247 | 241 | 241 | 184 | 188 | 260 | 260 | 273 | 273 | 133 | 139 | 186 | 204 | 238 | 244 | 263 | 275 | 150 | 150 | 143 | 163 | 211 | 215 | 211 | 213 | 203 | 207 | 190 | 210 | 242 | 242 | 170 | 172 | 217 | 227 | 153 | 165 | 291 | 295 | 302 | 316 | 218 | 232 | 165 | 169 | 237 | 237 | 212 | 222 | 247 | 247 | 94 | 94 | 199 | 201 | 81 | 85 | 263 | 263 | 182 | 190 |
| *Canaiolo nero N.* | 227 | 239 | 233 | 239 | 243 | 257 | 184 | 188 | 260 | 262 | 253 | 275 | 133 | 135 | 188 | 204 | 250 | 258 | 263 | 265 | 132 | 139 | 137 | 159 | 211 | 215 | 213 | 215 | 191 | 203 | 190 | 208 | 248 | 248 | 166 | 170 | 217 | 217 | 153 | 163 | 291 | 291 | 302 | 316 | 208 | 218 | 165 | 169 | 221 | 237 | 202 | 206 | 247 | 261 | 90 | 94 | 201 | 205 | 81 | 85 | 263 | 263 | 174 | 184 |
| Cardìn | 233 | 235 | 247 | 249 | 257 | 257 | 178 | 193 | 236 | 260 | 251 | 273 | 135 | 139 | 204 | 204 | 248 | 250 | 253 | 263 | 139 | 153 | 137 | 159 | 215 | 215 | 213 | 217 | 191 | 203 | 210 | 210 | 248 | 254 | 160 | 166 | 201 | 217 | 153 | 165 | 291 | 295 | 304 | 312 | 228 | 230 | 159 | 167 | 237 | 237 | 206 | 222 | 247 | 253 | 94 | 104 | 201 | 203 | 79 | 85 | 261 | 263 | 190 | 194 |
| Cascarolo | 227 | 227 | 249 | 253 | 241 | 269 | 184 | 188 | 220 | 270 | 241 | 263 | 133 | 151 | 200 | 200 | 238 | 246 | 263 | 294 | 126 | 163 | 139 | 141 | 211 | 211 | 209 | 213 | 203 | 203 | 208 | 208 | 254 | 264 | 166 | 166 | 201 | 209 | 153 | 153 | 289 | 291 | 310 | 316 | 222 | 230 | 165 | 165 | 237 | 237 | 206 | 206 | 249 | 249 | 100 | 108 | 201 | 203 | 79 | 85 | 263 | 263 | 184 | 194 |
| Cavazzina | 225 | 231 | 247 | 257 | 241 | 257 | 184 | 193 | 236 | 260 | 253 | 273 | 143 | 151 | 194 | 196 | 250 | 250 | 247 | 253 | 148 | 150 | 139 | 143 | 211 | 215 | 209 | 217 | 191 | 207 | 200 | 208 | 248 | 248 | 166 | 170 | 209 | 217 | 153 | 181 | 295 | 295 | 304 | 316 | 230 | 232 | 159 | 165 | 237 | 237 | 200 | 200 | 247 | 247 | 90 | 94 | 201 | 203 | 79 | 85 | 263 | 263 | 174 | 184 |
| Chardonnay B. | 233 | 237 | 239 | 243 | 241 | 257 | 180 | 188 | 220 | 230 | 241 | 273 | 137 | 143 | 188 | 196 | 242 | 244 | 253 | 275 | 139 | 153 | 159 | 163 | 213 | 215 | 209 | 217 | 201 | 207 | 200 | 210 | 248 | 248 | 156 | 170 | 203 | 237 | 139 | 153 | 289 | 295 | 310 | 312 | 208 | 226 | 159 | 165 | 237 | 237 | 206 | 222 | 247 | 253 | 94 | 98 | 201 | 205 | 79 | 85 | 263 | 265 | 180 | 184 |
| **Charmont B.** | 227 | 237 | 243 | 247 | 241 | 243 | 184 | 188 | 230 | 270 | 241 | 241 | 133 | 137 | 188 | 204 | 244 | 250 | 263 | 275 | 153 | 153 | 137 | 163 | 211 | 215 | 209 | 217 | 201 | 207 | 200 | 208 | 248 | 264 | 156 | 166 | 203 | 217 | 139 | 165 | 289 | 295 | 302 | 310 | 208 | 230 | 159 | 165 | 237 | 237 | 206 | 206 | 249 | 253 | 98 | 100 | 201 | 205 | 85 | 85 | 263 | 263 | 182 | 184 |
| **Chasselas blanc B.** | 227 | 235 | 239 | 247 | 243 | 257 | 184 | 188 | 220 | 270 | 241 | 241 | 133 | 143 | 194 | 204 | 250 | 258 | 263 | 294 | 126 | 153 | 137 | 139 | 211 | 215 | 209 | 213 | 201 | 207 | 200 | 208 | 248 | 264 | 166 | 166 | 203 | 217 | 153 | 165 | 291 | 295 | 302 | 302 | 228 | 230 | 165 | 165 | 237 | 237 | 206 | 222 | 247 | 249 | 94 | 100 | 201 | 201 | 81 | 85 | 263 | 263 | 182 | 194 |
| Chatus N. | 225 | 227 | 239 | 249 | 241 | 243 | 178 | 193 | 220 | 238 | 241 | 273 | 133 | 137 | 188 | 194 | 250 | 250 | 253 | 294 | 153 | 153 | 137 | 163 | 211 | 215 | 215 | 217 | 201 | 207 | 190 | 200 | 242 | 248 | 166 | 170 | 217 | 229 | 143 | 153 | 295 | 299 | 302 | 316 | 226 | 228 | 165 | 165 | 237 | 237 | 200 | 206 | 249 | 253 | 94 | 116 | 199 | 201 | 85 | 85 | 263 | 267 | 180 | 180 |
| Chiavennasca bianca | 235 | 245 | 247 | 249 | 241 | 243 | 184 | 188 | 238 | 270 | 251 | 273 | 139 | 155 | 194 | 200 | 242 | 244 | 263 | 269 | 150 | 153 | 137 | 143 | 203 | 211 | 209 | 213 | 201 | 207 | 208 | 208 | 242 | 248 | 166 | 170 | 203 | 217 | 153 | 181 | 289 | 299 | 302 | 302 | 218 | 230 | 159 | 165 | 237 | 237 | 206 | 222 | 247 | 247 | 94 | 104 | 201 | 201 | 79 | 81 | 263 | 263 | 194 | 196 |
| Citronino | 235 | 245 | 249 | 253 | 243 | 251 | 180 | 180 | 236 | 270 | 253 | 253 | 151 | 155 | 200 | 204 | 248 | 258 | 249 | 263 | 139 | 155 | 139 | 159 | 209 | 211 | 209 | 211 | 191 | 203 | 190 | 208 | 242 | 248 | 164 | 172 | 201 | 217 | 153 | 159 | 289 | 299 | 316 | 316 | 228 | 230 | 159 | 169 | 237 | 237 | 212 | 222 | 247 | 249 | 94 | 94 | 199 | 201 | 79 | 81 | 263 | 263 | 180 | 192 |
| Coccalona bianca | 231 | 245 | 239 | 249 | 241 | 241 | 180 | 193 | 236 | 260 | 251 | 263 | 133 | 143 | 188 | 200 | 250 | 258 | 249 | 296 | 153 | 153 | 137 | 163 | 211 | 211 | 209 | 209 | 191 | 201 | 208 | 208 | 248 | 248 | 160 | 166 | 217 | 217 | 153 | 153 | 291 | 295 | 312 | 316 | 208 | 228 | 165 | 169 | 221 | 237 | 200 | 222 | 247 | 261 | 100 | 100 | 201 | 205 | 79 | 81 | 263 | 263 | 190 | 192 |
| Coccalona nera (presumed, Rohrtraube blaurot) | 225 | 239 | 247 | 253 | 257 | 257 | 184 | 184 | 260 | 262 | 241 | 253 | 135 | 143 | 192 | 196 | 242 | 250 | 253 | 265 | 139 | 150 | 139 | 159 | 211 | 211 | 213 | 217 | 191 | 191 | 190 | 208 | 248 | 248 | 170 | 170 | 209 | 217 | 163 | 181 | 291 | 295 | 302 | 304 | 206 | 232 | 159 | 165 | 237 | 237 | 200 | 222 | 247 | 253 | 90 | 104 | 201 | 205 | 79 | 85 | 263 | 263 | 174 | 192 |
| Corbeau N. | 227 | 237 | 249 | 263 | 251 | 265 | 188 | 188 | 230 | 246 | 241 | 273 | 151 | 151 | 194 | 200 | 250 | 258 | 294 | 294 | 126 | 132 | 139 | 163 | 203 | 211 | 217 | 217 | 201 | 207 | 208 | 208 | 254 | 264 | 156 | 166 | 203 | 217 | 153 | 153 | 289 | 289 | 312 | 316 | 228 | 230 | 159 | 165 | 237 | 237 | 206 | 222 | 253 | 255 | 90 | 100 | 201 | 201 | 85 | 85 | 263 | 263 | 182 | 196 |
| Cortese B. | 225 | 235 | 249 | 249 | 243 | 251 | 178 | 182 | 236 | 250 | 253 | 253 | 133 | 151 | 200 | 202 | 246 | 250 | 265 | 265 | 148 | 150 | 143 | 143 | 209 | 211 | 207 | 209 | 191 | 203 | 190 | 208 | 242 | 248 | 170 | 170 | 209 | 227 | 163 | 171 | 291 | 291 | 312 | 320 | 208 | 230 | 165 | 165 | 221 | 237 | 196 | 200 | 247 | 249 | 94 | 108 | 201 | 207 | 79 | 81 | 263 | 263 | 176 | 190 |
| Croatina N. | 235 | 235 | 247 | 249 | 241 | 251 | 188 | 193 | 236 | 238 | 253 | 273 | 139 | 151 | 186 | 196 | 244 | 244 | 286 | 296 | 139 | 153 | 137 | 159 | 215 | 215 | 209 | 209 | 191 | 207 | 210 | 210 | 248 | 254 | 166 | 166 | 217 | 217 | 165 | 165 | 291 | 299 | 302 | 316 | 218 | 226 | 165 | 165 | 221 | 237 | 196 | 206 | 247 | 261 | 90 | 94 | 201 | 201 | 79 | 79 | 263 | 263 | 190 | 196 |
| Croetto | 225 | 245 | 247 | 251 | 243 | 251 | 178 | 193 | 236 | 260 | 263 | 273 | 133 | 151 | 186 | 188 | 248 | 250 | 247 | 247 | 139 | 150 | 143 | 159 | 215 | 215 | 209 | 209 | 203 | 207 | 210 | 210 | 254 | 264 | 164 | 170 | 203 | 217 | 165 | 165 | 295 | 307 | 310 | 316 | 218 | 230 | 169 | 177 | 237 | 237 | 200 | 206 | 249 | 253 | 90 | 100 | 201 | 203 | 79 | 79 | 263 | 263 | 184 | 188 |
| Crova (elliptic berry) (Uva crova) | 225 | 235 | 247 | 247 | 243 | 257 | 184 | 184 | 236 | 260 | 241 | 253 | 135 | 145 | 192 | 196 | 242 | 250 | 247 | 253 | 139 | 150 | 139 | 159 | 195 | 211 | 213 | 217 | 191 | 203 | 208 | 210 | 242 | 248 | 166 | 170 | 209 | 217 | 163 | 167 | 291 | 295 | 302 | 302 | 206 | 208 | 159 | 159 | 237 | 237 | 200 | 206 | 247 | 247 | 90 | 94 | 201 | 205 | 79 | 85 | 263 | 263 | 180 | 192 |
| Crovaro | 225 | 239 | 247 | 253 | 241 | 257 | 184 | 184 | 238 | 260 | 253 | 263 | 135 | 139 | 192 | 204 | 242 | 242 | 253 | 253 | 150 | 150 | 139 | 143 | 211 | 211 | 217 | 217 | 191 | 201 | 190 | 210 | 242 | 248 | 170 | 170 | 209 | 217 | 153 | 181 | 289 | 295 | 304 | 310 | 218 | 232 | 165 | 169 | 237 | 237 | 206 | 222 | 247 | 253 | 94 | 104 | 201 | 203 | 79 | 85 | 263 | 263 | 180 | 192 |
| Crovin (from Perti) | 227 | 239 | 239 | 253 | 251 | 251 | 178 | 180 | 250 | 270 | 273 | 273 | 143 | 151 | 200 | 204 | 250 | 260 | 253 | 263 | 139 | 155 | 139 | 159 | 211 | 223 | 209 | 213 | 193 | 207 | 190 | 208 | 248 | 248 | 160 | 164 | 207 | 217 | 139 | 153 | 295 | 295 | 312 | 316 | 210 | 230 | 165 | 165 | 237 | 239 | 206 | 206 | 249 | 249 | 100 | 116 | 201 | 203 | 79 | 85 | 263 | 263 | 186 | 190 |
| Dolcetto bianco | 225 | 233 | 243 | 247 | 241 | 257 | 178 | 182 | 236 | 246 | 273 | 273 | 139 | 143 | 188 | 204 | 246 | 248 | 263 | 263 | 139 | 153 | 137 | 159 | 215 | 215 | 213 | 217 | 207 | 207 | 210 | 210 | 248 | 254 | 160 | 170 | 201 | 217 | 165 | 165 | 291 | 299 | 316 | 316 | 218 | 230 | 165 | 169 | 237 | 237 | 218 | 222 | 249 | 249 | 94 | 100 | 201 | 203 | 79 | 83 | 263 | 263 | 180 | 190 |
| Dolcetto N. | 233 | 245 | 247 | 255 | 241 | 241 | 178 | 193 | 230 | 236 | 263 | 273 | 139 | 143 | 204 | 204 | 248 | 250 | 251 | 263 | 139 | 153 | 137 | 159 | 215 | 215 | 209 | 213 | 207 | 207 | 210 | 210 | 248 | 254 | 160 | 166 | 201 | 217 | 163 | 165 | 295 | 299 | 316 | 316 | 208 | 218 | 169 | 177 | 237 | 237 | 222 | 222 | 249 | 251 | 90 | 94 | 201 | 201 | 79 | 81 | 263 | 263 | 176 | 190 |
| Doux d'Henry N. | 235 | 245 | 247 | 249 | 251 | 257 | 188 | 193 | 230 | 260 | 253 | 273 | 143 | 151 | 186 | 196 | 244 | 250 | 251 | 253 | 150 | 153 | 137 | 163 | 215 | 215 | 209 | 217 | 191 | 207 | 210 | 210 | 248 | 254 | 166 | 166 | 217 | 217 | 153 | 163 | 291 | 295 | 316 | 316 | 228 | 230 | 165 | 165 | 237 | 237 | 206 | 222 | 253 | 261 | 90 | 104 | 201 | 201 | 79 | 85 | 263 | 263 | 188 | 190 |
| Durasa N. | 225 | 239 | 253 | 263 | 243 | 257 | 184 | 188 | 236 | 262 | 241 | 253 | 133 | 143 | 192 | 194 | 242 | 250 | 253 | 265 | 139 | 153 | 159 | 163 | 211 | 211 | 213 | 217 | 191 | 191 | 208 | 210 | 248 | 248 | 170 | 170 | 209 | 217 | 163 | 181 | 295 | 299 | 302 | 304 | 206 | 230 | 165 | 167 | 237 | 239 | 206 | 222 | 247 | 253 | 94 | 104 | 201 | 205 | 79 | 79 | 263 | 263 | 174 | 196 |
| Erbaluce B. | 225 | 227 | 247 | 253 | 243 | 269 | 178 | 184 | 246 | 270 | 241 | 253 | 145 | 151 | 194 | 200 | 242 | 254 | 271 | 294 | 150 | 153 | 137 | 143 | 209 | 211 | 209 | 213 | 203 | 203 | 208 | 208 | 254 | 254 | 166 | 170 | 217 | 217 | 153 | 153 | 289 | 291 | 312 | 316 | 208 | 222 | 159 | 165 | 237 | 237 | 206 | 206 | 249 | 249 | 90 | 108 | 201 | 201 | 81 | 85 | 261 | 263 | 192 | 194 |
| Feral accession | 233 | 237 | 255 | 257 | 241 | 271 | 188 | 193 | 230 | 248 | 273 | 273 | 139 | 151 | 194 | 204 | 250 | 250 | 251 | 263 | 139 | 153 | 137 | 159 | 215 | 215 | 213 | 213 | 207 | 207 | 200 | 210 | 248 | 254 | 160 | 160 | 201 | 209 | 163 | 165 | 291 | 295 | 304 | 316 | 206 | 218 | 159 | 177 | 237 | 237 | 200 | 222 | 247 | 249 | 90 | 94 | 201 | 201 | 81 | 85 | 263 | 263 | 176 | 190 |
| Fortana N. | 225 | 231 | 247 | 253 | 241 | 257 | 180 | 190 | 236 | 236 | 251 | 273 | 135 | 151 | 192 | 204 | 244 | 258 | 249 | 253 | 132 | 148 | 137 | 143 | 209 | 215 | 209 | 213 | 203 | 207 | 208 | 210 | 248 | 248 | 166 | 170 | 201 | 209 | 153 | 153 | 295 | 299 | 302 | 302 | 222 | 228 | 159 | 169 | 237 | 237 | 200 | 206 | 247 | 261 | 94 | 94 | 203 | 205 | 79 | 79 | 263 | 263 | 184 | 196 |
| Freisa N. | 225 | 231 | 247 | 249 | 241 | 243 | 184 | 188 | 246 | 270 | 241 | 273 | 133 | 155 | 194 | 200 | 250 | 250 | 263 | 271 | 153 | 163 | 149 | 163 | 211 | 215 | 213 | 213 | 191 | 207 | 208 | 210 | 248 | 254 | 166 | 170 | 217 | 217 | 153 | 171 | 291 | 299 | 302 | 316 | 208 | 222 | 159 | 167 | 237 | 239 | 198 | 206 | 247 | 249 | 90 | 104 | 201 | 201 | 79 | 79 | 263 | 263 | 180 | 182 |
| Fumin N. | 227 | 235 | 247 | 263 | 241 | 243 | 184 | 188 | 260 | 270 | 251 | 253 | 139 | 151 | 194 | 204 | 244 | 246 | 263 | 263 | 139 | 155 | 137 | 163 | 211 | 211 | 207 | 213 | 203 | 207 | 190 | 208 | 248 | 248 | 166 | 166 | 201 | 201 | 153 | 165 | 289 | 295 | 302 | 316 | 218 | 218 | 165 | 169 | 237 | 237 | 206 | 206 | 247 | 249 | 94 | 94 | 201 | 203 | 79 | 83 | 263 | 263 | 180 | 194 |
| Galletta | 227 | 235 | 243 | 249 | 257 | 257 | 178 | 184 | 238 | 246 | 241 | 273 | 143 | 143 | 188 | 204 | 242 | 254 | 251 | 263 | 132 | 139 | 159 | 163 | 203 | 215 | 209 | 217 | 191 | 207 | 208 | 210 | 248 | 254 | 160 | 166 | 201 | 227 | 153 | 159 | 289 | 295 | 304 | 312 | 218 | 228 | 159 | 169 | 237 | 237 | 206 | 206 | 247 | 253 | 94 | 94 | 201 | 203 | 79 | 85 | 263 | 263 | 180 | 192 |
| Galletto | 225 | 231 | 243 | 255 | 241 | 251 | 182 | 193 | 230 | 250 | 253 | 273 | 143 | 151 | 188 | 204 | 250 | 250 | 251 | 263 | 126 | 150 | 141 | 163 | 203 | 215 | 207 | 217 | 207 | 215 | 208 | 210 | 242 | 254 | 166 | 170 | 203 | 229 | 153 | 165 | 291 | 291 | 316 | 316 | 230 | 230 | 165 | 169 | 237 | 237 | 206 | 218 | 249 | 251 | 90 | 94 | 201 | 201 | 81 | 85 | 263 | 263 | 176 | 180 |
| Gamay N. | 233 | 237 | 239 | 249 | 241 | 241 | 180 | 188 | 220 | 248 | 241 | 273 | 133 | 137 | 194 | 204 | 242 | 244 | 253 | 263 | 139 | 153 | 159 | 163 | 213 | 215 | 209 | 217 | 201 | 207 | 200 | 210 | 248 | 248 | 156 | 170 | 203 | 223 | 153 | 165 | 289 | 299 | 310 | 312 | 222 | 226 | 159 | 165 | 237 | 239 | 200 | 222 | 247 | 249 | 94 | 98 | 201 | 205 | 85 | 85 | 263 | 263 | 176 | 180 |
| Gamba rossa N. (Gamba di pernice) | 225 | 231 | 239 | 253 | 241 | 257 | 178 | 188 | 260 | 260 | 273 | 273 | 133 | 143 | 186 | 200 | 238 | 244 | 263 | 275 | 150 | 153 | 137 | 163 | 203 | 211 | 211 | 217 | 203 | 207 | 208 | 208 | 248 | 248 | 160 | 170 | 209 | 227 | 159 | 165 | 295 | 295 | 302 | 316 | 218 | 218 | 165 | 169 | 237 | 245 | 206 | 222 | 247 | 247 | 94 | 94 | 201 | 205 | 81 | 85 | 263 | 263 | 182 | 190 |
| **Garanoir N.** | 227 | 237 | 249 | 257 | 241 | 251 | 180 | 188 | 220 | 250 | 265 | 273 | 137 | 151 | 194 | 194 | 244 | 244 | 253 | 263 | 139 | 150 | 159 | 163 | 215 | 223 | 209 | 217 | 201 | 207 | 190 | 200 | 248 | 248 | 156 | 166 | 203 | 223 | 153 | 153 | 289 | 295 | 312 | 312 | 222 | 226 | 159 | 165 | 237 | 245 | 200 | 222 | 247 | 253 | 94 | 94 | 201 | 201 | 81 | 85 | 263 | 263 | 180 | 184 |
| *Garganega B.* | 225 | 231 | 249 | 253 | 243 | 257 | 178 | 193 | 238 | 250 | 251 | 259 | 133 | 143 | 200 | 200 | 250 | 250 | 253 | 265 | 132 | 150 | 137 | 139 | 209 | 211 | 209 | 209 | 191 | 203 | 200 | 208 | 248 | 248 | 160 | 170 | 209 | 217 | 149 | 163 | 291 | 295 | 316 | 316 | 208 | 228 | 165 | 165 | 237 | 237 | 200 | 218 | 249 | 261 | 100 | 108 | 201 | 207 | 79 | 79 | 255 | 265 | 176 | 190 |
| Giamellotto (false) | 225 | 235 | 249 | 249 | 241 | 257 | 188 | 193 | 246 | 260 | 241 | 273 | 135 | 139 | 200 | 204 | 244 | 250 | 253 | 263 | 139 | 153 | 137 | 159 | 195 | 215 | 209 | 217 | 191 | 207 | 208 | 210 | 248 | 248 | 170 | 170 | 209 | 217 | 153 | 165 | 289 | 295 | 316 | 316 | 208 | 228 | 159 | 165 | 221 | 237 | 196 | 222 | 247 | 253 | 98 | 104 | 201 | 201 | 81 | 85 | 263 | 263 | 190 | 190 |
| Gouais blanc (Liseiret B.) | 233 | 239 | 239 | 249 | 241 | 257 | 178 | 180 | 230 | 248 | 251 | 273 | 133 | 143 | 196 | 204 | 236 | 242 | 263 | 275 | 139 | 139 | 159 | 159 | 211 | 213 | 209 | 209 | 203 | 207 | 190 | 210 | 248 | 248 | 160 | 170 | 203 | 223 | 139 | 165 | 295 | 299 | 302 | 310 | 208 | 222 | 159 | 165 | 237 | 239 | 200 | 206 | 247 | 249 | 98 | 100 | 201 | 205 | 79 | 85 | 263 | 263 | 176 | 184 |
| Granaccia bianca | 225 | 231 | 239 | 247 | 251 | 257 | 184 | 190 | 260 | 260 | 241 | 241 | 133 | 143 | 196 | 204 | 242 | 250 | 275 | 275 | 126 | 153 | 137 | 139 | 211 | 215 | 209 | 213 | 191 | 203 | 190 | 210 | 242 | 248 | 160 | 170 | 201 | 227 | 153 | 181 | 289 | 291 | 302 | 316 | 208 | 218 | 159 | 159 | 237 | 245 | 200 | 200 | 247 | 247 | 94 | 100 | 201 | 205 | 81 | 85 | 263 | 263 | 184 | 190 |
| Grandurey | 225 | 231 | 247 | 247 | 251 | 259 | 178 | 193 | 236 | 270 | 253 | 273 | 151 | 155 | 186 | 194 | 248 | 250 | 294 | 294 | 150 | 163 | 139 | 163 | 209 | 215 | 209 | 209 | 203 | 207 | 208 | 210 | 248 | 254 | 164 | 170 | 203 | 217 | 163 | 165 | 291 | 295 | 312 | 316 | 208 | 230 | 159 | 165 | 237 | 237 | 206 | 206 | 249 | 261 | 94 | 100 | 201 | 201 | 79 | 85 | 255 | 261 | 176 | 188 |
| Grec rouge | 227 | 235 | 247 | 253 | 243 | 257 | 184 | 184 | 238 | 260 | 257 | 263 | 133 | 143 | 188 | 194 | 238 | 250 | 253 | 263 | 132 | 150 | 137 | 143 | 215 | 215 | 209 | 217 | 201 | 203 | 210 | 210 | 248 | 254 | 160 | 170 | 217 | 227 | 153 | 159 | 291 | 295 | 302 | 312 | 208 | 230 | 153 | 165 | 237 | 245 | 198 | 200 | 247 | 247 | 100 | 108 | 203 | 205 | 81 | 85 | 263 | 263 | 182 | 196 |
| Grignolino N. | 225 | 245 | 247 | 255 | 241 | 257 | 180 | 188 | 260 | 260 | 241 | 265 | 133 | 135 | 194 | 204 | 250 | 258 | 253 | 263 | 126 | 150 | 141 | 163 | 211 | 211 | 209 | 217 | 191 | 201 | 208 | 210 | 248 | 254 | 164 | 170 | 201 | 203 | 153 | 153 | 291 | 291 | 302 | 316 | 218 | 228 | 159 | 165 | 237 | 237 | 206 | 206 | 247 | 261 | 90 | 104 | 201 | 207 | 79 | 85 | 263 | 265 | 180 | 182 |
| Gris | 225 | 245 | 247 | 247 | 241 | 243 | 178 | 178 | 230 | 260 | 253 | 273 | 139 | 143 | 186 | 200 | 248 | 258 | 251 | 263 | 139 | 150 | 159 | 163 | 195 | 215 | 213 | 217 | 203 | 207 | 208 | 210 | 248 | 254 | 164 | 170 | 209 | 217 | 163 | 165 | 291 | 295 | 302 | 302 | 230 | 230 | 169 | 177 | 237 | 237 | 206 | 206 | 247 | 261 | 90 | 94 | 201 | 201 | 81 | 81 | 263 | 263 | 176 | 184 |
| Grisa (from Cumiana) | 225 | 235 | 247 | 247 | 243 | 257 | 184 | 184 | 238 | 246 | 241 | 253 | 135 | 139 | 196 | 200 | 242 | 258 | 251 | 251 | 139 | 150 | 159 | 163 | 195 | 211 | 213 | 217 | 191 | 203 | 208 | 208 | 248 | 254 | 166 | 170 | 201 | 209 | 165 | 181 | 295 | 295 | 302 | 316 | 208 | 228 | 159 | 169 | 237 | 237 | 206 | 222 | 247 | 253 | 94 | 94 | 201 | 201 | 81 | 85 | 263 | 263 | 184 | 190 |
| Groppello gentile N. | 225 | 227 | 247 | 253 | 245 | 257 | 184 | 184 | 238 | 246 | 241 | 251 | 143 | 155 | 194 | 204 | 238 | 250 | 247 | 294 | 126 | 153 | 141 | 163 | 211 | 215 | 213 | 213 | 191 | 201 | 200 | 210 | 248 | 254 | 160 | 170 | 201 | 209 | 153 | 181 | 291 | 295 | 310 | 316 | 218 | 228 | 159 | 165 | 237 | 237 | 198 | 222 | 247 | 249 | 94 | 100 | 199 | 203 | 79 | 85 | 261 | 263 | 184 | 190 |
| *Harslevelu* | 225 | 231 | 239 | 247 | 241 | 243 | 178 | 178 | 230 | 250 | 265 | 273 | 133 | 145 | 188 | 204 | 236 | 250 | 263 | 275 | 150 | 159 | 143 | 163 | 211 | 213 | 209 | 209 | 201 | 207 | 190 | 208 | 242 | 256 | 170 | 170 | 217 | 217 | 139 | 165 | 295 | 295 | 316 | 316 | 226 | 228 | 159 | 159 | 237 | 237 | 196 | 236 | 247 | 249 | 94 | 94 | 199 | 205 | 73 | 79 | 263 | 263 | 176 | 178 |
| **Heptakilo** | 225 | 231 | 239 | 251 | 247 | 251 | 178 | 193 | 246 | 246 | 251 | 273 | 149 | 153 | 188 | 204 | 246 | 256 | 253 | 265 | 126 | 148 | 139 | 143 | 215 | 223 | 207 | 213 | 191 | 207 | 190 | 208 | 248 | 254 | 168 | 170 | 205 | 207 | 141 | 181 | 291 | 291 | 302 | 316 | 208 | 230 | 169 | 169 | 237 | 245 | 218 | 236 | 247 | 247 | 94 | 100 | 201 | 207 | 79 | 81 | 263 | 263 | 190 | 192 |
| Lacrima Cristi | 225 | 235 | 247 | 247 | 243 | 257 | 184 | 193 | 260 | 260 | 241 | 273 | 139 | 143 | 186 | 200 | 250 | 258 | 253 | 263 | 139 | 153 | 137 | 159 | 195 | 215 | 209 | 213 | 191 | 207 | 208 | 210 | 248 | 248 | 166 | 170 | 201 | 217 | 163 | 165 | 291 | 299 | 302 | 316 | 208 | 230 | 165 | 169 | 237 | 237 | 206 | 206 | 247 | 253 | 104 | 104 | 201 | 201 | 81 | 85 | 263 | 263 | 180 | 190 |
| Lambrusca di Alessandria N. | 225 | 239 | 253 | 255 | 251 | 257 | 178 | 193 | 230 | 270 | 265 | 273 | 143 | 151 | 200 | 204 | 250 | 250 | 251 | 263 | 139 | 150 | 157 | 159 | 211 | 223 | 209 | 213 | 201 | 207 | 190 | 190 | 248 | 248 | 160 | 164 | 207 | 217 | 139 | 153 | 295 | 295 | 316 | 316 | 208 | 230 | 165 | 165 | 239 | 245 | 206 | 222 | 247 | 249 | 100 | 100 | 203 | 205 | 79 | 85 | 255 | 263 | 180 | 190 |
| Lambrusca pignata | 227 | 245 | 239 | 247 | 243 | 257 | 188 | 193 | 238 | 260 | 273 | 273 | 133 | 143 | 186 | 194 | 244 | 250 | 253 | 294 | 153 | 153 | 137 | 137 | 215 | 215 | 209 | 217 | 207 | 207 | 200 | 210 | 242 | 254 | 166 | 166 | 217 | 229 | 153 | 163 | 295 | 295 | 316 | 316 | 228 | 230 | 165 | 165 | 237 | 237 | 200 | 206 | 249 | 261 | 90 | 94 | 199 | 201 | 79 | 85 | 263 | 263 | 180 | 188 |
| Lambrusca vittona | 235 | 237 | 249 | 261 | 243 | 257 | 188 | 193 | 238 | 260 | 253 | 273 | 139 | 143 | 194 | 204 | 246 | 250 | 253 | 253 | 139 | 139 | 159 | 159 | 195 | 215 | 213 | 217 | 203 | 207 | 208 | 210 | 248 | 248 | 166 | 166 | 201 | 203 | 153 | 165 | 289 | 295 | 302 | 316 | 230 | 232 | 159 | 169 | 237 | 237 | 206 | 206 | 247 | 247 | 102 | 104 | 201 | 201 | 81 | 85 | 263 | 263 | 184 | 192 |
| Lambruschetta | 225 | 225 | 243 | 247 | 241 | 243 | 178 | 188 | 236 | 246 | 253 | 257 | 133 | 139 | 188 | 196 | 250 | 250 | 263 | 263 | 139 | 153 | 159 | 163 | 215 | 215 | 209 | 213 | 195 | 215 | 200 | 210 | 248 | 254 | 170 | 170 | 203 | 209 | 165 | 165 | 289 | 291 | 316 | 316 | 218 | 218 | 159 | 165 | 237 | 237 | 206 | 218 | 247 | 247 | 90 | 94 | 201 | 201 | 79 | 81 | 263 | 263 | 184 | 194 |
| *Lambrusco Barghi N.* | 227 | 239 | 233 | 257 | 251 | 257 | 184 | 193 | 236 | 260 | 251 | 273 | 143 | 151 | 192 | 204 | 250 | 250 | 249 | 253 | 139 | 155 | 159 | 193 | 211 | 211 | 209 | 213 | 191 | 203 | 190 | 208 | 248 | 248 | 166 | 170 | 209 | 217 | 153 | 165 | 289 | 295 | 310 | 316 | 222 | 230 | 159 | 159 | 237 | 237 | 200 | 206 | 253 | 255 | 94 | 102 | 205 | 205 | 79 | 85 | 263 | 263 | 184 | 184 |
| Lambrusco del pellegrino N. (L. di Fiorano) | 225 | 231 | 239 | 253 | 257 | 259 | 184 | 184 | 236 | 262 | 253 | 263 | 143 | 155 | 188 | 192 | 242 | 250 | 263 | 265 | 139 | 153 | 159 | 163 | 211 | 211 | 211 | 213 | 191 | 215 | 190 | 200 | 248 | 248 | 160 | 170 | 203 | 217 | 163 | 165 | 295 | 299 | 302 | 302 | 206 | 226 | 165 | 165 | 237 | 239 | 200 | 200 | 247 | 253 | 90 | 98 | 201 | 205 | 79 | 85 | 263 | 263 | 176 | 192 |
| Lambrusco Maestri N. | 227 | 231 | 253 | 257 | 257 | 257 | 190 | 193 | 236 | 266 | 251 | 253 | 135 | 155 | 192 | 202 | 244 | 250 | 253 | 269 | 148 | 153 | 143 | 163 | 209 | 215 | 213 | 213 | 191 | 203 | 208 | 208 | 248 | 248 | 166 | 166 | 209 | 211 | 153 | 153 | 291 | 299 | 302 | 310 | 222 | 228 | 159 | 169 | 237 | 237 | 206 | 206 | 249 | 261 | 94 | 130 | 203 | 205 | 79 | 79 | 261 | 263 | 184 | 196 |
| *Lambrusco Salamino N.* | 227 | 231 | 233 | 263 | 251 | 257 | 184 | 188 | 238 | 260 | 241 | 251 | 151 | 151 | 194 | 204 | 246 | 250 | 253 | 267 | 155 | 163 | 149 | 193 | 209 | 215 | 213 | 217 | 191 | 203 | 208 | 208 | 248 | 248 | 166 | 166 | 201 | 229 | 153 | 153 | 289 | 295 | 302 | 316 | 222 | 222 | 159 | 159 | 237 | 237 | 200 | 204 | 247 | 247 | 94 | 102 | 201 | 205 | 79 | 85 | 263 | 263 | 180 | 184 |
| Luglienga bianca (S. Anna di Lipsia B.) | 227 | 235 | 247 | 247 | 243 | 251 | 184 | 184 | 236 | 248 | 253 | 263 | 145 | 155 | 192 | 194 | 238 | 250 | 263 | 296 | 126 | 126 | 139 | 141 | 195 | 195 | 209 | 209 | 191 | 203 | 210 | 210 | 248 | 254 | 166 | 166 | 203 | 217 | 153 | 165 | 289 | 291 | 312 | 320 | 208 | 208 | 165 | 165 | 237 | 237 | 206 | 218 | 247 | 247 | 94 | 100 | 201 | 201 | 81 | 85 | 255 | 263 | 194 | 196 |
| Luglienga moscata | 227 | 235 | 233 | 247 | 251 | 251 | 178 | 184 | 236 | 248 | 263 | 273 | 133 | 155 | 192 | 196 | 250 | 254 | 243 | 296 | 126 | 139 | 139 | 159 | 195 | 215 | 209 | 213 | 203 | 207 | 210 | 210 | 254 | 264 | 160 | 166 | 203 | 203 | 165 | 165 | 289 | 295 | 312 | 312 | 208 | 228 | 159 | 165 | 237 | 237 | 206 | 218 | 247 | 249 | 100 | 100 | 199 | 201 | 79 | 85 | 261 | 263 | 188 | 196 |
| Luglienga nera | 225 | 231 | 247 | 247 | 243 | 243 | 178 | 184 | 248 | 270 | 263 | 273 | 133 | 145 | 192 | 194 | 248 | 250 | 263 | 263 | 150 | 153 | 143 | 163 | 195 | 215 | 213 | 217 | 191 | 207 | 210 | 210 | 248 | 254 | 166 | 170 | 217 | 217 | 153 | 153 | 291 | 299 | 310 | 312 | 218 | 218 | 159 | 169 | 237 | 237 | 200 | 218 | 249 | 249 | 90 | 94 | 201 | 201 | 79 | 85 | 263 | 263 | 182 | 192 |
| Maiolina N. | 225 | 231 | 247 | 253 | 257 | 259 | 180 | 184 | 230 | 246 | 263 | 273 | 135 | 155 | 194 | 194 | 250 | 258 | 247 | 253 | 150 | 155 | 143 | 193 | 211 | 211 | 209 | 217 | 191 | 207 | 200 | 210 | 242 | 248 | 168 | 170 | 201 | 217 | 165 | 181 | 289 | 295 | 316 | 316 | 218 | 230 | 159 | 159 | 237 | 239 | 206 | 222 | 247 | 251 | 94 | 104 | 199 | 201 | 79 | 79 | 263 | 263 | 184 | 196 |
| Malvasia aromatica di Parma (Malvasia Casalini B.) | 227 | 235 | 233 | 247 | 251 | 257 | 178 | 184 | 248 | 260 | 241 | 265 | 133 | 135 | 196 | 196 | 242 | 254 | 243 | 253 | 126 | 139 | 141 | 159 | 211 | 215 | 217 | 217 | 191 | 207 | 208 | 208 | 248 | 264 | 160 | 170 | 201 | 217 | 153 | 165 | 291 | 299 | 312 | 316 | 228 | 228 | 159 | 159 | 237 | 239 | 206 | 206 | 247 | 249 | 100 | 104 | 199 | 201 | 79 | 85 | 261 | 263 | 180 | 184 |
| Malvasia bianca (from Vignale) | 227 | 245 | 233 | 239 | 241 | 257 | 178 | 180 | 248 | 260 | 263 | 265 | 135 | 143 | 188 | 196 | 242 | 258 | 243 | 249 | 139 | 153 | 137 | 159 | 211 | 211 | 209 | 217 | 191 | 201 | 208 | 208 | 248 | 248 | 160 | 166 | 201 | 217 | 153 | 165 | 291 | 291 | 312 | 312 | 208 | 228 | 159 | 169 | 237 | 237 | 206 | 222 | 247 | 247 | 100 | 104 | 199 | 201 | 81 | 85 | 263 | 263 | 184 | 190 |
| *Malvasia bianca lunga B.* | 225 | 239 | 239 | 253 | 241 | 243 | 178 | 178 | 250 | 256 | 253 | 257 | 145 | 145 | 196 | 200 | 242 | 250 | 253 | 253 | 132 | 139 | 137 | 159 | 211 | 213 | 209 | 209 | 205 | 207 | 190 | 210 | 248 | 248 | 170 | 170 | 201 | 203 | 153 | 159 | 295 | 299 | 320 | 320 | 208 | 210 | 163 | 165 | 237 | 239 | 206 | 212 | 247 | 249 | 94 | 114 | 201 | 201 | 79 | 89 | 255 | 263 | 174 | 178 |
| Malvasia di Boca | 227 | 231 | 239 | 247 | 241 | 243 | 178 | 184 | 230 | 260 | 241 | 241 | 139 | 155 | 194 | 204 | 238 | 238 | 253 | 253 | 126 | 139 | 141 | 159 | 211 | 215 | 209 | 213 | 191 | 201 | 190 | 210 | 242 | 248 | 166 | 166 | 201 | 203 | 159 | 181 | 295 | 295 | 302 | 316 | 208 | 218 | 159 | 165 | 237 | 237 | 206 | 206 | 249 | 253 | 94 | 94 | 201 | 201 | 81 | 85 | 263 | 263 | 184 | 194 |
| Malvasia di Candia aromatica B. | 227 | 227 | 233 | 233 | 257 | 257 | 178 | 184 | 236 | 248 | 241 | 265 | 135 | 143 | 196 | 204 | 250 | 254 | 243 | 249 | 139 | 139 | 159 | 159 | 209 | 211 | 215 | 217 | 191 | 207 | 208 | 208 | 248 | 264 | 160 | 166 | 217 | 217 | 165 | 165 | 295 | 299 | 302 | 316 | 218 | 228 | 159 | 165 | 237 | 239 | 206 | 206 | 249 | 253 | 94 | 100 | 199 | 205 | 85 | 85 | 261 | 263 | 184 | 196 |
| Malvasia di Casorzo N. | 227 | 239 | 233 | 253 | 251 | 257 | 178 | 178 | 230 | 248 | 265 | 273 | 133 | 151 | 196 | 200 | 242 | 250 | 243 | 251 | 139 | 150 | 157 | 159 | 211 | 223 | 213 | 217 | 201 | 207 | 190 | 208 | 248 | 248 | 160 | 164 | 201 | 207 | 139 | 165 | 291 | 295 | 312 | 316 | 208 | 228 | 159 | 165 | 237 | 245 | 206 | 222 | 247 | 249 | 100 | 104 | 199 | 203 | 79 | 79 | 255 | 263 | 180 | 190 |
| Malvasia di Schierano N. | 235 | 235 | 249 | 249 | 251 | 257 | 178 | 188 | 236 | 248 | 273 | 273 | 133 | 135 | 186 | 196 | 244 | 254 | 243 | 296 | 139 | 139 | 159 | 159 | 215 | 215 | 213 | 217 | 207 | 207 | 210 | 210 | 248 | 264 | 166 | 166 | 209 | 217 | 165 | 181 | 291 | 295 | 304 | 316 | 228 | 230 | 159 | 165 | 237 | 237 | 206 | 206 | 249 | 253 | 100 | 108 | 199 | 201 | 79 | 85 | 263 | 263 | 188 | 190 |
| Malvasia moscata B. | 225 | 227 | 233 | 239 | 243 | 251 | 178 | 180 | 246 | 260 | 253 | 265 | 133 | 133 | 188 | 196 | 254 | 258 | 253 | 263 | 139 | 139 | 159 | 159 | 211 | 211 | 209 | 217 | 207 | 215 | 208 | 210 | 248 | 248 | 160 | 170 | 201 | 227 | 153 | 165 | 291 | 299 | 302 | 312 | 218 | 228 | 159 | 165 | 237 | 239 | 206 | 206 | 247 | 249 | 94 | 104 | 199 | 201 | 85 | 85 | 263 | 263 | 180 | 184 |
| Malvasia nera (from Costa V.) | 225 | 227 | 233 | 253 | 251 | 257 | 178 | 178 | 230 | 260 | 241 | 265 | 133 | 143 | 196 | 200 | 250 | 254 | 251 | 253 | 126 | 139 | 141 | 159 | 211 | 223 | 209 | 217 | 191 | 201 | 190 | 208 | 248 | 248 | 160 | 160 | 201 | 207 | 153 | 153 | 291 | 295 | 316 | 316 | 208 | 228 | 159 | 165 | 237 | 239 | 206 | 206 | 249 | 249 | 100 | 104 | 201 | 205 | 79 | 85 | 255 | 263 | 180 | 180 |
| Malvasia nera a grappolo corto | 227 | 245 | 247 | 247 | 251 | 257 | 178 | 180 | 236 | 260 | 241 | 273 | 135 | 151 | 186 | 196 | 254 | 258 | 253 | 263 | 126 | 153 | 137 | 141 | 215 | 215 | 209 | 217 | 191 | 201 | 208 | 210 | 248 | 248 | 160 | 164 | 209 | 217 | 153 | 165 | 291 | 295 | 316 | 316 | 208 | 228 | 159 | 165 | 237 | 237 | 206 | 206 | 249 | 251 | 100 | 104 | 201 | 201 | 85 | 85 | 263 | 263 | 176 | 184 |
| Malvasia nera lunga N. | 227 | 231 | 233 | 249 | 241 | 257 | 178 | 184 | 246 | 260 | 241 | 273 | 135 | 155 | 196 | 200 | 250 | 254 | 253 | 271 | 126 | 163 | 141 | 149 | 211 | 211 | 213 | 217 | 191 | 207 | 208 | 208 | 254 | 264 | 160 | 166 | 217 | 217 | 153 | 153 | 291 | 299 | 312 | 316 | 222 | 228 | 159 | 169 | 237 | 239 | 206 | 206 | 247 | 247 | 90 | 100 | 199 | 201 | 79 | 85 | 263 | 263 | 180 | 184 |
| Marzemino N. | 225 | 231 | 239 | 263 | 243 | 257 | 184 | 188 | 236 | 238 | 241 | 263 | 133 | 133 | 194 | 194 | 242 | 250 | 253 | 253 | 150 | 153 | 143 | 163 | 211 | 215 | 209 | 213 | 191 | 191 | 200 | 210 | 242 | 248 | 166 | 170 | 203 | 217 | 181 | 181 | 291 | 299 | 302 | 302 | 208 | 230 | 159 | 169 | 237 | 239 | 206 | 222 | 247 | 253 | 94 | 94 | 201 | 203 | 79 | 79 | 261 | 263 | 184 | 196 |
| Mayolet Rs. | 225 | 237 | 247 | 263 | 241 | 243 | 184 | 188 | 246 | 260 | 251 | 253 | 133 | 139 | 194 | 204 | 238 | 250 | 251 | 263 | 126 | 150 | 141 | 163 | 211 | 215 | 207 | 213 | 191 | 201 | 208 | 210 | 248 | 254 | 160 | 170 | 201 | 223 | 153 | 159 | 291 | 295 | 310 | 320 | 208 | 218 | 159 | 169 | 237 | 237 | 206 | 218 | 247 | 249 | 90 | 94 | 201 | 205 | 79 | 83 | 261 | 263 | 192 | 196 |
| Millegusti | 227 | 231 | 247 | 249 | 251 | 251 | 178 | 178 | 236 | 248 | 251 | 265 | 133 | 139 | 186 | 204 | 254 | 258 | 243 | 263 | 126 | 153 | 137 | 141 | 203 | 215 | 213 | 213 | 203 | 207 | 208 | 210 | 248 | 254 | 160 | 172 | 203 | 217 | 153 | 165 | 291 | 291 | 302 | 312 | 228 | 232 | 159 | 169 | 237 | 237 | 206 | 222 | 249 | 249 | 94 | 108 | 199 | 203 | 79 | 81 | 263 | 263 | 180 | 184 |
| Moissan | 231 | 245 | 247 | 255 | 241 | 251 | 178 | 193 | 230 | 236 | 263 | 273 | 143 | 151 | 186 | 204 | 250 | 258 | 251 | 251 | 139 | 150 | 159 | 163 | 209 | 215 | 209 | 217 | 201 | 207 | 190 | 210 | 248 | 254 | 164 | 166 | 203 | 217 | 163 | 165 | 291 | 295 | 302 | 316 | 208 | 230 | 165 | 177 | 237 | 237 | 206 | 222 | 251 | 261 | 90 | 100 | 201 | 201 | 79 | 81 | 255 | 263 | 176 | 188 |
| Montanera N. | 231 | 231 | 247 | 249 | 257 | 259 | 180 | 184 | 230 | 260 | 263 | 273 | 143 | 155 | 194 | 194 | 250 | 258 | 253 | 263 | 153 | 155 | 163 | 193 | 211 | 215 | 209 | 215 | 191 | 207 | 200 | 210 | 248 | 248 | 168 | 170 | 217 | 217 | 165 | 165 | 295 | 295 | 302 | 316 | 230 | 232 | 153 | 159 | 237 | 237 | 206 | 222 | 247 | 255 | 94 | 104 | 201 | 203 | 79 | 79 | 263 | 263 | 196 | 196 |
| Moradella N. | 235 | 239 | 247 | 249 | 257 | 257 | 184 | 188 | 260 | 262 | 253 | 273 | 143 | 143 | 196 | 196 | 242 | 244 | 253 | 265 | 150 | 155 | 139 | 163 | 211 | 215 | 217 | 217 | 191 | 207 | 208 | 210 | 248 | 248 | 166 | 170 | 201 | 217 | 153 | 163 | 291 | 295 | 304 | 316 | 206 | 208 | 159 | 165 | 237 | 237 | 200 | 222 | 247 | 253 | 104 | 108 | 201 | 205 | 77 | 85 | 263 | 263 | 174 | 190 |
| Morio muskat | 225 | 227 | 247 | 249 | 245 | 251 | 178 | 193 | 230 | 248 | 273 | 273 | 133 | 151 | 186 | 204 | 248 | 254 | 243 | 275 | 126 | 126 | 139 | 141 | 203 | 215 | 209 | 213 | 207 | 207 | 190 | 210 | 248 | 264 | 160 | 170 | 217 | 223 | 165 | 165 | 289 | 295 | 312 | 312 | 228 | 230 | 159 | 165 | 237 | 237 | 206 | 206 | 247 | 249 | 94 | 100 | 199 | 205 | 79 | 85 | 255 | 261 | 188 | 194 |
| Moscato bianco B. | 227 | 235 | 233 | 249 | 243 | 251 | 178 | 193 | 248 | 270 | 265 | 273 | 133 | 133 | 186 | 196 | 250 | 254 | 243 | 263 | 126 | 139 | 141 | 159 | 211 | 215 | 213 | 217 | 207 | 207 | 208 | 210 | 248 | 264 | 160 | 166 | 203 | 217 | 139 | 165 | 291 | 295 | 312 | 316 | 228 | 230 | 159 | 159 | 237 | 237 | 206 | 206 | 249 | 249 | 94 | 100 | 199 | 199 | 79 | 79 | 261 | 263 | 184 | 188 |
| Moscato bianco precoce | 227 | 233 | 233 | 247 | 243 | 257 | 178 | 182 | 246 | 248 | 265 | 273 | 133 | 143 | 196 | 204 | 246 | 254 | 243 | 263 | 126 | 153 | 137 | 141 | 211 | 215 | 217 | 217 | 207 | 207 | 208 | 210 | 248 | 254 | 160 | 160 | 217 | 217 | 139 | 165 | 295 | 299 | 316 | 316 | 218 | 230 | 159 | 169 | 237 | 237 | 206 | 218 | 249 | 249 | 94 | 100 | 199 | 201 | 79 | 79 | 263 | 263 | 188 | 190 |
| **Moscato d'Amburgo N. (Muscat of Hamburg)** | 231 | 237 | 247 | 249 | 251 | 257 | 178 | 184 | 238 | 246 | 273 | 273 | 135 | 149 | 186 | 192 | 238 | 254 | 253 | 294 | 126 | 155 | 139 | 193 | 211 | 215 | 213 | 213 | 191 | 207 | 208 | 210 | 248 | 254 | 166 | 168 | 203 | 205 | 159 | 165 | 295 | 295 | 316 | 316 | 208 | 230 | 165 | 169 | 237 | 237 | 206 | 206 | 247 | 249 | 94 | 94 | 201 | 207 | 79 | 83 | 263 | 263 | 180 | 188 |
| Moscato di Scanzo N. | 235 | 239 | 239 | 249 | 243 | 257 | 178 | 193 | 236 | 248 | 241 | 273 | 133 | 135 | 186 | 194 | 250 | 254 | 243 | 294 | 132 | 139 | 159 | 163 | 215 | 215 | 217 | 217 | 191 | 207 | 200 | 210 | 248 | 264 | 166 | 166 | 211 | 217 | 165 | 171 | 291 | 295 | 310 | 316 | 206 | 228 | 159 | 159 | 237 | 237 | 206 | 222 | 249 | 253 | 94 | 100 | 199 | 201 | 79 | 79 | 261 | 263 | 188 | 196 |

| | | | | | | | | | | | | | | | | | | | | | | | | | | | | | | | | | | | | | | | | | | | | | | | | | | | | | | | | | | | | | | | | |
|---|---|---|---|---|---|---|---|---|---|---|---|---|---|---|---|---|---|---|---|---|---|---|---|---|---|---|---|---|---|---|---|---|---|---|---|---|---|---|---|---|---|---|---|---|---|---|---|---|---|---|---|---|---|---|---|---|---|---|---|---|---|---|---|---|
| Moscato giallo B. | 227 | 239 | 239 | 249 | 243 | 257 | 178 | 178 | 238 | 248 | 259 | 273 | 133 | 143 | 186 | 188 | 248 | 254 | 263 | 294 | 126 | 139 | 141 | 159 | 209 | 211 | 213 | 217 | 191 | 207 | 208 | 208 | 254 | 264 | 160 | 170 | 201 | 217 | 165 | 165 | 295 | 295 | 312 | 320 | 218 | 228 | 159 | 169 | 237 | 237 | 206 | 218 | 247 | 249 | 94 | 100 | 199 | 201 | 79 | 81 | 263 | 263 | 184 | 184 |
| Moscato nero (from Borbera valley) | 227 | 239 | 247 | 249 | 251 | 257 | 184 | 193 | 248 | 260 | 253 | 265 | 133 | 135 | 186 | 196 | 250 | 250 | 243 | 253 | 126 | 150 | 139 | 141 | 211 | 215 | 213 | 217 | 191 | 207 | 190 | 210 | 248 | 248 | 160 | 170 | 217 | 217 | 165 | 181 | 295 | 295 | 304 | 312 | 230 | 232 | 159 | 169 | 237 | 237 | 200 | 206 | 249 | 253 | 90 | 100 | 199 | 205 | 79 | 85 | 263 | 263 | 184 | 192 |
| Moscato nero di Acqui N. | 225 | 235 | 247 | 249 | 251 | 257 | 178 | 184 | 248 | 260 | 253 | 265 | 133 | 135 | 186 | 196 | 242 | 254 | 243 | 253 | 126 | 150 | 139 | 141 | 211 | 211 | 217 | 217 | 191 | 207 | 208 | 210 | 248 | 264 | 166 | 170 | 209 | 217 | 139 | 181 | 291 | 295 | 302 | 312 | 206 | 228 | 159 | 159 | 237 | 237 | 206 | 222 | 249 | 253 | 90 | 100 | 199 | 205 | 79 | 85 | 261 | 263 | 174 | 188 |
| Moscato rosa Rs. | 235 | 239 | 239 | 249 | 243 | 257 | 178 | 193 | 250 | 270 | 265 | 273 | 133 | 135 | 186 | 188 | 248 | 254 | 253 | 263 | 126 | 139 | 141 | 159 | 211 | 215 | 209 | 217 | 201 | 207 | 208 | 210 | 242 | 264 | 160 | 166 | 217 | 223 | 139 | 159 | 291 | 295 | 312 | 316 | 208 | 230 | 159 | 163 | 237 | 245 | 206 | 212 | 249 | 249 | 94 | 100 | 199 | 205 | 79 | 81 | 255 | 261 | 188 | 196 |
| Moschato mavro | 227 | 235 | 245 | 249 | 243 | 257 | 178 | 184 | 248 | 260 | 265 | 273 | 133 | 135 | 186 | 202 | 238 | 254 | 243 | 265 | 126 | 132 | 137 | 141 | 211 | 215 | 213 | 213 | 207 | 207 | 208 | 210 | 248 | 254 | 160 | 170 | 217 | 217 | 153 | 165 | 295 | 295 | 312 | 316 | 208 | 228 | 153 | 159 | 237 | 245 | 206 | 206 | 249 | 261 | 90 | 100 | 199 | 201 | 79 | 89 | 261 | 263 | 188 | 188 |
| Mossano (from Canavese area) | 235 | 245 | 247 | 255 | 251 | 257 | 193 | 193 | 236 | 246 | 263 | 273 | 135 | 151 | 196 | 204 | 250 | 250 | 251 | 251 | 139 | 150 | 159 | 163 | 211 | 215 | 209 | 217 | 201 | 207 | 208 | 210 | 248 | 248 | 164 | 166 | 203 | 209 | 153 | 165 | 291 | 295 | 304 | 316 | 208 | 232 | 159 | 165 | 237 | 237 | 206 | 222 | 253 | 261 | 100 | 104 | 201 | 201 | 81 | 85 | 263 | 263 | 176 | 190 |
| Mostarino | 225 | 231 | 247 | 249 | 241 | 245 | 184 | 184 | 238 | 270 | 253 | 263 | 133 | 155 | 186 | 200 | 238 | 250 | 263 | 263 | 150 | 153 | 163 | 163 | 211 | 215 | 209 | 213 | 203 | 215 | 208 | 210 | 248 | 254 | 160 | 170 | 209 | 227 | 159 | 181 | 289 | 295 | 302 | 316 | 218 | 230 | 165 | 177 | 237 | 237 | 206 | 206 | 247 | 247 | 94 | 108 | 201 | 201 | 79 | 85 | 263 | 263 | 176 | 190 |
| Mulinè | 225 | 235 | 247 | 249 | 241 | 241 | 188 | 188 | 270 | 270 | 263 | 273 | 133 | 139 | 200 | 204 | 244 | 246 | 263 | 263 | 150 | 153 | 137 | 163 | 211 | 215 | 207 | 209 | 201 | 207 | 190 | 210 | 248 | 254 | 164 | 170 | 209 | 217 | 153 | 153 | 289 | 291 | 302 | 312 | 208 | 218 | 165 | 169 | 237 | 237 | 206 | 218 | 249 | 249 | 90 | 100 | 199 | 201 | 83 | 85 | 263 | 263 | 180 | 190 |
| Muscat rouge de Madère | 225 | 227 | 247 | 249 | 243 | 251 | 178 | 182 | 248 | 260 | 253 | 273 | 133 | 133 | 186 | 204 | 244 | 254 | 243 | 269 | 126 | 139 | 141 | 159 | 215 | 215 | 209 | 217 | 191 | 207 | 210 | 210 | 254 | 264 | 160 | 170 | 203 | 217 | 139 | 163 | 291 | 295 | 302 | 316 | 218 | 230 | 159 | 165 | 237 | 237 | 200 | 206 | 247 | 249 | 94 | 108 | 199 | 201 | 79 | 79 | 263 | 263 | 184 | 188 |
| Nascetta B. | 225 | 231 | 247 | 249 | 241 | 241 | 178 | 188 | 270 | 270 | 241 | 273 | 133 | 139 | 200 | 204 | 244 | 258 | 263 | 263 | 126 | 150 | 141 | 143 | 211 | 215 | 209 | 217 | 191 | 203 | 190 | 210 | 248 | 248 | 170 | 172 | 209 | 209 | 141 | 153 | 289 | 295 | 302 | 316 | 218 | 222 | 165 | 167 | 237 | 237 | 200 | 222 | 247 | 249 | 90 | 94 | 199 | 201 | 79 | 85 | 263 | 263 | 190 | 194 |
| Nebbiolo (false) 217 | 231 | 231 | 247 | 247 | 241 | 243 | 188 | 193 | 260 | 270 | 241 | 273 | 133 | 155 | 194 | 196 | 250 | 250 | 253 | 263 | 126 | 153 | 137 | 141 | 211 | 211 | 213 | 217 | 191 | 207 | 208 | 208 | 248 | 248 | 166 | 172 | 209 | 217 | 165 | 171 | 295 | 299 | 302 | 302 | 218 | 232 | 159 | 159 | 237 | 239 | 206 | 222 | 247 | 253 | 94 | 94 | 201 | 201 | 79 | 85 | 263 | 263 | 180 | 194 |
| Nebbiolo d'Antom | 231 | 239 | 247 | 249 | 241 | 249 | 178 | 184 | 238 | 246 | 263 | 273 | 133 | 139 | 188 | 204 | 238 | 250 | 263 | 294 | 139 | 153 | 137 | 159 | 211 | 215 | 209 | 209 | 187 | 201 | 190 | 210 | 248 | 248 | 160 | 160 | 203 | 217 | 159 | 165 | 289 | 295 | 310 | 316 | 222 | 228 | 165 | 165 | 237 | 237 | 200 | 206 | 247 | 253 | 94 | 100 | 199 | 205 | 85 | 85 | 263 | 267 | 180 | 194 |
| Nebbiolo di Dronero (false) | 225 | 245 | 249 | 249 | 243 | 257 | 178 | 193 | 220 | 230 | 253 | 273 | 133 | 143 | 188 | 196 | 250 | 252 | 253 | 294 | 153 | 153 | 137 | 137 | 211 | 215 | 209 | 215 | 191 | 207 | 190 | 210 | 242 | 248 | 166 | 170 | 217 | 217 | 153 | 163 | 295 | 295 | 316 | 316 | 226 | 228 | 159 | 165 | 237 | 237 | 200 | 222 | 253 | 261 | 94 | 104 | 201 | 201 | 85 | 85 | 263 | 267 | 180 | 190 |
| Nebbiolo gabardin | 225 | 225 | 249 | 263 | 243 | 257 | 193 | 193 | 220 | 246 | 263 | 273 | 133 | 143 | 188 | 194 | 238 | 250 | 253 | 263 | 139 | 153 | 137 | 159 | 211 | 211 | 209 | 215 | 201 | 207 | 190 | 210 | 242 | 248 | 160 | 170 | 203 | 217 | 143 | 159 | 291 | 299 | 316 | 316 | 208 | 226 | 159 | 165 | 237 | 237 | 200 | 206 | 247 | 253 | 94 | 116 | 199 | 201 | 81 | 85 | 263 | 267 | 180 | 194 |
| Nebbiolo N. | 231 | 235 | 247 | 249 | 241 | 243 | 184 | 188 | 238 | 270 | 241 | 263 | 155 | 155 | 194 | 200 | 242 | 250 | 263 | 263 | 126 | 163 | 141 | 149 | 211 | 211 | 209 | 213 | 191 | 191 | 208 | 210 | 248 | 248 | 166 | 166 | 217 | 217 | 153 | 171 | 291 | 299 | 302 | 316 | 218 | 222 | 159 | 159 | 237 | 239 | 198 | 206 | 247 | 249 | 94 | 104 | 201 | 201 | 79 | 85 | 263 | 263 | 180 | 196 |
| Nebbiolo rosé | 225 | 231 | 247 | 253 | 243 | 259 | 180 | 188 | 236 | 270 | 241 | 253 | 155 | 155 | 194 | 194 | 250 | 258 | 263 | 263 | 126 | 163 | 141 | 149 | 211 | 211 | 209 | 213 | 191 | 207 | 200 | 210 | 242 | 248 | 166 | 170 | 217 | 217 | 153 | 171 | 291 | 299 | 302 | 316 | 218 | 222 | 159 | 165 | 237 | 239 | 198 | 206 | 247 | 249 | 94 | 94 | 201 | 201 | 79 | 85 | 263 | 263 | 176 | 180 |
| Nebùe | 225 | 227 | 233 | 255 | 251 | 257 | 178 | 184 | 238 | 248 | 241 | 265 | 133 | 143 | 196 | 204 | 242 | 248 | 253 | 271 | 126 | 153 | 137 | 141 | 209 | 211 | 209 | 217 | 191 | 201 | 190 | 208 | 248 | 264 | 160 | 160 | 217 | 217 | 153 | 153 | 291 | 295 | 302 | 312 | 208 | 228 | 159 | 165 | 237 | 245 | 206 | 206 | 247 | 249 | 94 | 100 | 201 | 201 | 79 | 85 | 261 | 261 | 176 | 180 |
| Negrello | 225 | 231 | 239 | 249 | 241 | 243 | 180 | 188 | 220 | 230 | 241 | 241 | 133 | 137 | 188 | 196 | 246 | 258 | 253 | 253 | 126 | 153 | 137 | 139 | 195 | 203 | 209 | 213 | 195 | 195 | 208 | 210 | 248 | 254 | 166 | 170 | 201 | 217 | 131 | 159 | 289 | 295 | 316 | 316 | 208 | 226 | 159 | 165 | 237 | 237 | 200 | 206 | 247 | 249 | 100 | 116 | 201 | 201 | 79 | 85 | 263 | 263 | 180 | 184 |
| Negrera (from Valtellina) | 231 | 235 | 247 | 249 | 241 | 243 | 184 | 190 | 238 | 270 | 241 | 263 | 137 | 155 | 194 | 200 | 242 | 250 | 263 | 296 | 126 | 139 | 141 | 159 | 211 | 211 | 209 | 213 | 191 | 195 | 208 | 210 | 248 | 248 | 166 | 168 | 217 | 229 | 163 | 171 | 291 | 299 | 302 | 316 | 208 | 222 | 159 | 167 | 237 | 239 | 198 | 200 | 247 | 253 | 104 | 108 | 201 | 201 | 79 | 81 | 263 | 263 | 180 | 194 |
| Neirera | 225 | 245 | 247 | 249 | 241 | 241 | 178 | 178 | 230 | 246 | 253 | 273 | 133 | 143 | 186 | 200 | 248 | 258 | 251 | 263 | 139 | 153 | 137 | 159 | 211 | 215 | 209 | 209 | 207 | 215 | 208 | 210 | 248 | 248 | 164 | 170 | 217 | 217 | 153 | 165 | 295 | 295 | 302 | 316 | 208 | 208 | 169 | 177 | 237 | 237 | 206 | 222 | 247 | 261 | 94 | 100 | 201 | 201 | 81 | 85 | 255 | 263 | 180 | 188 |
| Neretta cuneese N. | 225 | 237 | 247 | 263 | 241 | 257 | 180 | 193 | 220 | 246 | 263 | 273 | 139 | 143 | 194 | 204 | 238 | 258 | 253 | 263 | 139 | 153 | 159 | 163 | 211 | 215 | 209 | 209 | 201 | 207 | 210 | 210 | 242 | 248 | 156 | 160 | 203 | 217 | 153 | 159 | 291 | 295 | 316 | 316 | 208 | 230 | 159 | 165 | 237 | 237 | 206 | 206 | 247 | 247 | 94 | 94 | 199 | 201 | 81 | 85 | 263 | 263 | 182 | 194 |
| Neretto di Bairo N. | 225 | 235 | 247 | 247 | 243 | 243 | 184 | 188 | 260 | 270 | 263 | 273 | 133 | 155 | 194 | 196 | 242 | 250 | 253 | 263 | 139 | 163 | 149 | 151 | 211 | 211 | 209 | 209 | 191 | 207 | 208 | 210 | 248 | 248 | 166 | 170 | 209 | 217 | 171 | 181 | 291 | 295 | 304 | 316 | 222 | 230 | 159 | 165 | 237 | 237 | 206 | 206 | 247 | 249 | 94 | 104 | 201 | 201 | 79 | 79 | 263 | 263 | 180 | 196 |
| Neretto di Marengo | 225 | 245 | 249 | 255 | 241 | 257 | 178 | 193 | 230 | 238 | 251 | 265 | 137 | 143 | 200 | 204 | 248 | 250 | 251 | 271 | 150 | 153 | 137 | 157 | 209 | 211 | 209 | 209 | 191 | 201 | 190 | 208 | 248 | 248 | 160 | 164 | 203 | 217 | 153 | 153 | 291 | 295 | 302 | 316 | 208 | 208 | 159 | 165 | 237 | 245 | 206 | 222 | 247 | 251 | 94 | 100 | 201 | 205 | 79 | 85 | 255 | 261 | 176 | 180 |
| Neretto di Salto | 225 | 245 | 247 | 255 | 251 | 257 | 184 | 193 | 236 | 260 | 273 | 273 | 135 | 151 | 196 | 204 | 242 | 250 | 226 | 243 | 126 | 139 | 141 | 159 | 215 | 215 | 209 | 217 | 207 | 207 | 208 | 210 | 248 | 254 | 164 | 170 | 201 | 217 | 163 | 171 | 291 | 291 | 316 | 316 | 218 | 230 | 159 | 165 | 237 | 237 | 206 | 222 | 247 | 261 | 90 | 94 | 201 | 201 | 81 | 85 | 255 | 263 | 180 | 188 |
| Neretto duro (Balau) | 225 | 235 | 247 | 247 | 243 | 257 | 184 | 193 | 236 | 236 | 241 | 253 | 133 | 135 | 194 | 196 | 258 | 258 | 296 | 296 | 139 | 150 | 159 | 163 | 211 | 211 | 213 | 217 | 191 | 201 | 190 | 208 | 248 | 254 | 166 | 170 | 201 | 217 | 159 | 181 | 291 | 299 | 312 | 316 | 228 | 230 | 165 | 169 | 237 | 237 | 206 | 206 | 247 | 253 | 90 | 108 | 201 | 201 | 79 | 81 | 263 | 263 | 190 | 190 |
| *Neyret N.* | 225 | 227 | 247 | 263 | 241 | 243 | 184 | 188 | 260 | 270 | 263 | 273 | 133 | 139 | 194 | 204 | 238 | 244 | 263 | 263 | 126 | 153 | 137 | 141 | 209 | 215 | 207 | 213 | 203 | 207 | 208 | 210 | 248 | 254 | 166 | 170 | 201 | 223 | 153 | 165 | 289 | 291 | 302 | 316 | 218 | 218 | 165 | 167 | 237 | 237 | 206 | 222 | 247 | 247 | 90 | 94 | 201 | 203 | 83 | 85 | 263 | 263 | 176 | 190 |
| Orange muscat (M. fleur d'oranger) | 227 | 235 | 247 | 249 | 243 | 251 | 178 | 184 | 248 | 270 | 241 | 273 | 133 | 133 | 186 | 204 | 250 | 254 | 243 | 263 | 139 | 153 | 137 | 159 | 211 | 215 | 209 | 213 | 201 | 207 | 208 | 210 | 248 | 264 | 160 | 166 | 203 | 203 | 165 | 165 | 295 | 295 | 302 | 316 | 228 | 230 | 159 | 165 | 237 | 237 | 206 | 206 | 247 | 249 | 94 | 100 | 199 | 201 | 79 | 81 | 261 | 263 | 184 | 194 |
| Orsanella | 227 | 231 | 249 | 253 | 241 | 257 | 184 | 188 | 260 | 270 | 241 | 253 | 143 | 155 | 194 | 202 | 250 | 250 | 263 | 275 | 126 | 132 | 137 | 141 | 211 | 211 | 213 | 217 | 191 | 207 | 200 | 208 | 242 | 248 | 160 | 166 | 209 | 217 | 153 | 153 | 291 | 295 | 312 | 316 | 222 | 228 | 165 | 165 | 237 | 237 | 198 | 218 | 249 | 249 | 94 | 94 | 201 | 203 | 79 | 85 | 261 | 263 | 180 | 190 |
| *Ortrugo B.* | 225 | 237 | 247 | 253 | 243 | 259 | 180 | 184 | 220 | 250 | 241 | 263 | 133 | 155 | 194 | 200 | 250 | 258 | 253 | 263 | 126 | 150 | 141 | 143 | 211 | 211 | 209 | 213 | 191 | 201 | 208 | 210 | 242 | 242 | 166 | 170 | 209 | 217 | 153 | 181 | 291 | 299 | 316 | 316 | 208 | 230 | 159 | 165 | 237 | 237 | 206 | 218 | 247 | 249 | 94 | 94 | 201 | 205 | 79 | 85 | 263 | 263 | 176 | 194 |
| Parmesana | 235 | 239 | 243 | 247 | 243 | 257 | 184 | 184 | 248 | 262 | 253 | 273 | 133 | 143 | 188 | 192 | 242 | 250 | 263 | 265 | 126 | 139 | 141 | 159 | 211 | 211 | 209 | 213 | 191 | 207 | 190 | 208 | 242 | 248 | 166 | 170 | 209 | 217 | 131 | 163 | 295 | 295 | 302 | 316 | 206 | 208 | 165 | 165 | 237 | 237 | 206 | 222 | 247 | 249 | 90 | 94 | 203 | 205 | 85 | 85 | 263 | 263 | 176 | 192 |
| Patlassa (long internodes, presumed) | 225 | 239 | 247 | 253 | 241 | 257 | 180 | 188 | 236 | 260 | 241 | 251 | 135 | 143 | 192 | 200 | 242 | 258 | 275 | 286 | 126 | 139 | 141 | 153 | 211 | 211 | 209 | 217 | 191 | 201 | 190 | 208 | 242 | 248 | 156 | 170 | 217 | 217 | 153 | 159 | 291 | 291 | 312 | 316 | 226 | 232 | 153 | 165 | 221 | 237 | 222 | 224 | 247 | 249 | 94 | 104 | 201 | 201 | 79 | 85 | 263 | 263 | 180 | 190 |
| Pelaverga N. | 227 | 235 | 247 | 253 | 241 | 243 | 178 | 188 | 260 | 270 | 241 | 273 | 133 | 151 | 194 | 194 | 250 | 258 | 263 | 263 | 150 | 155 | 139 | 163 | 211 | 215 | 209 | 217 | 191 | 203 | 200 | 210 | 248 | 254 | 160 | 164 | 201 | 217 | 153 | 153 | 291 | 291 | 312 | 316 | 208 | 218 | 159 | 165 | 237 | 237 | 206 | 222 | 247 | 249 | 90 | 94 | 201 | 201 | 79 | 79 | 263 | 263 | 176 | 182 |
| Pelaverga piccolo N. | 233 | 239 | 243 | 253 | 251 | 251 | 178 | 188 | 236 | 250 | 251 | 253 | 133 | 151 | 188 | 200 | 248 | 250 | 263 | 263 | 126 | 132 | 137 | 141 | 203 | 211 | 209 | 211 | 203 | 215 | 208 | 208 | 248 | 248 | 160 | 166 | 209 | 229 | 153 | 153 | 291 | 291 | 312 | 316 | 228 | 230 | 153 | 169 | 237 | 237 | 196 | 218 | 249 | 251 | 94 | 94 | 201 | 205 | 81 | 85 | 263 | 263 | 180 | 196 |
| **Perla di Csaba B. (Csaba gyoengye)** | 235 | 235 | 247 | 249 | 243 | 243 | 178 | 180 | 220 | 270 | 273 | 273 | 133 | 155 | 186 | 204 | 254 | 258 | 263 | 294 | 139 | 139 | 159 | 159 | 215 | 223 | 209 | 213 | 201 | 207 | 190 | 210 | 242 | 264 | 166 | 166 | 203 | 221 | 165 | 181 | 291 | 295 | 312 | 316 | 216 | 230 | 159 | 165 | 221 | 237 | 206 | 222 | 247 | 249 | 94 | 100 | 199 | 201 | 79 | 85 | 263 | 263 | 180 | 194 |
| Petit rouge N. | 225 | 227 | 247 | 247 | 241 | 243 | 188 | 188 | 246 | 260 | 253 | 263 | 133 | 139 | 194 | 204 | 244 | 246 | 251 | 263 | 126 | 155 | 139 | 141 | 209 | 215 | 213 | 217 | 203 | 203 | 208 | 210 | 248 | 248 | 166 | 170 | 201 | 201 | 153 | 165 | 289 | 291 | 302 | 310 | 218 | 222 | 165 | 169 | 237 | 237 | 206 | 222 | 247 | 249 | 94 | 94 | 203 | 203 | 79 | 83 | 263 | 263 | 176 | 190 |
| *Petite Arvine B.* | 237 | 237 | 257 | 263 | 243 | 251 | 184 | 188 | 238 | 246 | 241 | 251 | 133 | 151 | 194 | 194 | 238 | 250 | 253 | 263 | 126 | 139 | 139 | 151 | 195 | 211 | 213 | 217 | 191 | 201 | 208 | 210 | 248 | 264 | 156 | 156 | 229 | 229 | 153 | 153 | 291 | 295 | 302 | 302 | 208 | 222 | 159 | 169 | 237 | 237 | 200 | 200 | 247 | 247 | 94 | 94 | 201 | 203 | 79 | 81 | 261 | 263 | 176 | 180 |
| Picolit (false) | 235 | 239 | 253 | 263 | 243 | 257 | 184 | 190 | 248 | 260 | 253 | 253 | 133 | 143 | 192 | 194 | 242 | 250 | 253 | 263 | 126 | 150 | 139 | 139 | 211 | 215 | 209 | 217 | 191 | 203 | 190 | 208 | 248 | 248 | 166 | 170 | 203 | 217 | 165 | 181 | 291 | 291 | 302 | 310 | 206 | 222 | 165 | 165 | 237 | 237 | 200 | 222 | 247 | 253 | 90 | 102 | 201 | 205 | 79 | 85 | 263 | 263 | 192 | 196 |
| Pignola N. | 231 | 235 | 253 | 263 | 241 | 243 | 180 | 188 | 236 | 238 | 241 | 263 | 155 | 155 | 194 | 194 | 250 | 258 | 294 | 294 | 150 | 153 | 143 | 163 | 211 | 211 | 209 | 213 | 191 | 191 | 200 | 210 | 242 | 248 | 166 | 166 | 217 | 217 | 153 | 171 | 291 | 299 | 302 | 316 | 218 | 230 | 159 | 165 | 237 | 239 | 206 | 206 | 247 | 249 | 94 | 94 | 201 | 201 | 79 | 85 | 263 | 263 | 180 | 196 |
| **Pinot nero N. (Pinot noir)** | 227 | 237 | 239 | 243 | 241 | 251 | 184 | 188 | 220 | 238 | 241 | 273 | 137 | 151 | 188 | 194 | 238 | 244 | 253 | 253 | 126 | 153 | 139 | 163 | 215 | 215 | 215 | 217 | 201 | 207 | 200 | 200 | 248 | 248 | 156 | 166 | 203 | 237 | 153 | 153 | 289 | 295 | 312 | 316 | 226 | 230 | 165 | 165 | 237 | 237 | 206 | 222 | 247 | 253 | 94 | 116 | 201 | 201 | 85 | 85 | 263 | 265 | 180 | 184 |
| Plassa N. | 225 | 225 | 247 | 249 | 257 | 257 | 180 | 193 | 246 | 280 | 257 | 273 | 135 | 143 | 204 | 204 | 250 | 258 | 251 | 263 | 139 | 139 | 159 | 159 | 211 | 211 | 209 | 217 | 205 | 207 | 190 | 208 | 242 | 248 | 170 | 170 | 201 | 217 | 153 | 163 | 291 | 307 | 312 | 316 | 218 | 228 | 157 | 159 | 237 | 237 | 206 | 206 | 247 | 253 | 98 | 104 | 201 | 201 | 81 | 85 | 263 | 263 | 180 | 190 |
| Prié blanc B. | 227 | 237 | 233 | 247 | 241 | 251 | 184 | 188 | 236 | 246 | 253 | 273 | 133 | 155 | 194 | 196 | 238 | 250 | 251 | 296 | 126 | 126 | 139 | 141 | 195 | 215 | 209 | 209 | 191 | 207 | 208 | 210 | 248 | 254 | 156 | 166 | 203 | 223 | 141 | 153 | 289 | 295 | 316 | 320 | 208 | 222 | 159 | 165 | 237 | 237 | 206 | 206 | 247 | 247 | 90 | 94 | 201 | 203 | 81 | 85 | 255 | 255 | 176 | 194 |
| Primaticcia | 235 | 239 | 249 | 255 | 251 | 251 | 178 | 188 | 230 | 236 | 273 | 273 | 151 | 151 | 196 | 204 | 244 | 250 | 251 | 296 | 150 | 153 | 137 | 157 | 211 | 215 | 209 | 213 | 207 | 207 | 190 | 210 | 248 | 254 | 164 | 166 | 207 | 217 | 139 | 165 | 295 | 299 | 316 | 316 | 218 | 230 | 159 | 165 | 237 | 245 | 206 | 222 | 249 | 261 | 90 | 100 | 201 | 203 | 79 | 79 | 263 | 263 | 190 | 190 |
| Quagliano N. (Bouteillan) | 225 | 225 | 239 | 243 | 241 | 269 | 178 | 188 | 250 | 260 | 241 | 251 | 133 | 143 | 186 | 188 | 250 | 254 | 263 | 275 | 130 | 139 | 159 | 159 | 203 | 209 | 209 | 209 | 191 | 205 | 208 | 208 | 248 | 248 | 170 | 170 | 217 | 227 | 149 | 153 | 295 | 295 | 302 | 316 | 228 | 232 | 159 | 165 | 237 | 245 | 212 | 222 | 247 | 253 | 94 | 100 | 201 | 201 | 81 | 81 | 263 | 263 | 182 | 190 |
| Rachina | 231 | 231 | 239 | 247 | 243 | 259 | 188 | 188 | 230 | 248 | 241 | 263 | 133 | 155 | 188 | 194 | 246 | 246 | 253 | 263 | 126 | 153 | 137 | 139 | 203 | 215 | 209 | 213 | 195 | 203 | 208 | 210 | 254 | 254 | 164 | 166 | 201 | 217 | 131 | 165 | 295 | 299 | 316 | 316 | 222 | 226 | 165 | 165 | 237 | 237 | 200 | 206 | 247 | 251 | 90 | 100 | 201 | 201 | 79 | 85 | 263 | 263 | 180 | 194 |
| Rapallino | 225 | 231 | 249 | 253 | 241 | 243 | 180 | 184 | 238 | 250 | 251 | 257 | 133 | 155 | 200 | 200 | 250 | 258 | 263 | 263 | 126 | 139 | 141 | 159 | 211 | 215 | 209 | 213 | 191 | 191 | 208 | 210 | 248 | 248 | 166 | 170 | 217 | 227 | 153 | 181 | 291 | 299 | 302 | 312 | 218 | 230 | 165 | 165 | 237 | 237 | 206 | 212 | 247 | 249 | 94 | 108 | 201 | 201 | 79 | 85 | 263 | 263 | 190 | 192 |
| Rastajola | 227 | 239 | 247 | 247 | 241 | 257 | 184 | 184 | 260 | 262 | 253 | 273 | 143 | 155 | 194 | 196 | 242 | 250 | 253 | 265 | 126 | 150 | 139 | 141 | 211 | 211 | 213 | 217 | 191 | 207 | 190 | 210 | 248 | 248 | 170 | 170 | 201 | 217 | 171 | 181 | 291 | 299 | 302 | 316 | 206 | 218 | 159 | 159 | 237 | 239 | 206 | 222 | 247 | 253 | 90 | 94 | 201 | 205 | 85 | 85 | 263 | 263 | 180 | 192 |
| Refosco dal peduncolo rosso N. | 225 | 231 | 239 | 247 | 243 | 257 | 180 | 188 | 236 | 238 | 251 | 263 | 133 | 133 | 194 | 194 | 250 | 258 | 253 | 253 | 150 | 153 | 143 | 163 | 211 | 215 | 209 | 217 | 191 | 201 | 200 | 210 | 248 | 248 | 166 | 170 | 203 | 217 | 171 | 181 | 291 | 295 | 302 | 316 | 208 | 230 | 159 | 169 | 237 | 237 | 212 | 222 | 247 | 253 | 94 | 104 | 201 | 203 | 79 | 79 | 261 | 263 | 176 | 184 |
| Refosco gentile | 225 | 225 | 247 | 263 | 243 | 259 | 178 | 184 | 236 | 246 | 253 | 263 | 133 | 155 | 194 | 204 | 250 | 258 | 251 | 263 | 155 | 163 | 149 | 163 | 211 | 215 | 209 | 213 | 191 | 215 | 208 | 210 | 248 | 254 | 170 | 170 | 203 | 229 | 153 | 181 | 291 | 295 | 302 | 312 | 218 | 222 | 165 | 169 | 237 | 237 | 204 | 206 | 247 | 247 | 94 | 100 | 201 | 201 | 79 | 85 | 263 | 263 | 180 | 196 |
| Refosco nostrano N. | 225 | 231 | 249 | 263 | 259 | 265 | 184 | 184 | 236 | 270 | 253 | 263 | 151 | 155 | 194 | 200 | 242 | 250 | 263 | 263 | 139 | 163 | 149 | 159 | 209 | 211 | 209 | 209 | 191 | 215 | 208 | 208 | 242 | 248 | 166 | 170 | 203 | 217 | 181 | 181 | 291 | 295 | 312 | 316 | 218 | 230 | 159 | 169 | 237 | 237 | 206 | 206 | 247 | 247 | 94 | 100 | 201 | 203 | 79 | 81 | 263 | 263 | 176 | 180 |
| Riesling B. | 225 | 233 | 249 | 257 | 251 | 257 | 180 | 188 | 230 | 236 | 253 | 273 | 143 | 151 | 194 | 204 | 242 | 244 | 253 | 263 | 139 | 153 | 137 | 159 | 203 | 213 | 209 | 217 | 203 | 207 | 208 | 210 | 248 | 248 | 170 | 170 | 223 | 227 | 139 | 165 | 289 | 299 | 302 | 312 | 222 | 230 | 159 | 169 | 237 | 237 | 200 | 206 | 249 | 249 | 98 | 102 | 201 | 205 | 79 | 85 | 263 | 263 | 176 | 184 |
| Riesling italico B. | 225 | 237 | 247 | 257 | 257 | 271 | 184 | 188 | 248 | 260 | 241 | 273 | 135 | 151 | 194 | 196 | 250 | 250 | 253 | 263 | 139 | 139 | 159 | 159 | 211 | 215 | 213 | 217 | 191 | 207 | 200 | 208 | 248 | 248 | 160 | 170 | 209 | 223 | 153 | 163 | 291 | 299 | 302 | 304 | 206 | 222 | 159 | 165 | 237 | 239 | 200 | 222 | 247 | 253 | 90 | 94 | 201 | 205 | 79 | 85 | 263 | 263 | 174 | 176 |
| Rossara N. (Schiava N.) | 225 | 233 | 239 | 253 | 241 | 257 | 178 | 184 | 246 | 248 | 251 | 273 | 133 | 143 | 194 | 196 | 236 | 250 | 263 | 269 | 126 | 139 | 141 | 159 | 211 | 211 | 209 | 213 | 201 | 207 | 190 | 200 | 242 | 248 | 170 | 170 | 201 | 203 | 165 | 181 | 291 | 299 | 310 | 316 | 208 | 218 | 159 | 165 | 237 | 239 | 200 | 218 | 247 | 251 | 94 | 98 | 203 | 205 | 79 | 85 | 263 | 263 | 184 | 184 |
| Rossese bianco B. | 225 | 227 | 247 | 253 | 241 | 259 | 178 | 184 | 238 | 260 | 253 | 273 | 139 | 155 | 194 | 204 | 248 | 250 | 263 | 294 | 132 | 155 | 137 | 139 | 211 | 211 | 213 | 217 | 203 | 207 | 200 | 208 | 242 | 248 | 160 | 170 | 203 | 217 | 153 | 165 | 291 | 291 | 304 | 316 | 218 | 218 | 165 | 169 | 237 | 237 | 218 | 218 | 247 | 249 | 94 | 94 | 201 | 203 | 83 | 85 | 261 | 263 | 184 | 190 |
| Rossola (false) | 231 | 245 | 249 | 263 | 241 | 243 | 184 | 188 | 220 | 270 | 241 | 273 | 137 | 139 | 194 | 200 | 238 | 244 | 263 | 284 | 150 | 153 | 137 | 163 | 205 | 211 | 213 | 213 | 191 | 201 | 200 | 208 | 242 | 248 | 166 | 170 | 203 | 231 | 153 | 153 | 295 | 299 | 302 | 310 | 230 | 230 | 159 | 165 | 237 | 237 | 200 | 206 | 247 | 247 | 94 | 94 | 201 | 201 | 79 | 81 | 263 | 263 | 180 | 194 |
| Rossola nera N. | 231 | 231 | 253 | 263 | 241 | 243 | 184 | 188 | 236 | 270 | 257 | 263 | 155 | 155 | 194 | 194 | 242 | 250 | 263 | 263 | 126 | 163 | 141 | 149 | 203 | 211 | 209 | 213 | 191 | 207 | 200 | 208 | 242 | 248 | 166 | 166 | 203 | 217 | 153 | 171 | 291 | 299 | 302 | 316 | 218 | 230 | 159 | 165 | 237 | 239 | 206 | 206 | 247 | 249 | 94 | 94 | 201 | 201 | 79 | 85 | 263 | 263 | 176 | 180 |
| Rossola verde | 225 | 231 | 247 | 253 | 241 | 243 | 178 | 184 | 260 | 270 | 253 | 263 | 139 | 155 | 194 | 204 | 242 | 248 | 263 | 263 | 139 | 163 | 149 | 159 | 195 | 211 | 209 | 211 | 191 | 203 | 200 | 208 | 248 | 248 | 166 | 170 | 203 | 217 | 165 | 171 | 289 | 291 | 302 | 316 | 208 | 230 | 159 | 165 | 237 | 237 | 206 | 222 | 249 | 249 | 94 | 104 | 201 | 201 | 81 | 85 | 263 | 263 | 180 | 184 |
| Rossoletta | 231 | 231 | 249 | 253 | 241 | 259 | 184 | 188 | 236 | 270 | 257 | 273 | 137 | 155 | 194 | 200 | 238 | 250 | 263 | 263 | 126 | 153 | 137 | 141 | 209 | 211 | 209 | 213 | 207 | 207 | 200 | 208 | 248 | 248 | 166 | 166 | 203 | 217 | 153 | 165 | 291 | 299 | 302 | 316 | 222 | 228 | 159 | 165 | 237 | 239 | 198 | 206 | 247 | 249 | 94 | 94 | 199 | 201 | 79 | 81 | 261 | 263 | 176 | 180 |
| Ruché N. | 235 | 235 | 233 | 247 | 251 | 257 | 178 | 188 | 236 | 248 | 265 | 273 | 135 | 151 | 186 | 196 | 244 | 254 | 243 | 296 | 139 | 139 | 159 | 159 | 211 | 215 | 209 | 217 | 191 | 207 | 208 | 210 | 254 | 264 | 166 | 170 | 201 | 217 | 153 | 165 | 299 | 299 | 302 | 316 | 218 | 228 | 159 | 165 | 237 | 239 | 196 | 206 | 249 | 261 | 90 | 100 | 199 | 201 | 79 | 79 | 263 | 263 | 180 | 196 |
| *Sangiovese N.* | 225 | 235 | 239 | 263 | 243 | 243 | 178 | 184 | 236 | 246 | 253 | 257 | 133 | 133 | 194 | 196 | 242 | 258 | 263 | 263 | 132 | 155 | 137 | 139 | 211 | 211 | 209 | 215 | 203 | 205 | 190 | 208 | 242 | 248 | 168 | 170 | 217 | 217 | 141 | 141 | 289 | 291 | 308 | 308 | 208 | 222 | 159 | 165 | 237 | 237 | 206 | 206 | 247 | 247 | 90 | 94 | 201 | 201 | 79 | 85 | 263 | 263 | 192 | 196 |
| **Sauvignon blanc B.** | 227 | 231 | 239 | 257 | 243 | 251 | 174 | 188 | 236 | 238 | 241 | 257 | 133 | 151 | 188 | 194 | 244 | 246 | 263 | 294 | 126 | 150 | 139 | 143 | 209 | 215 | 217 | 217 | 205 | 207 | 200 | 208 | 242 | 248 | 160 | 166 | 223 | 237 | 165 | 171 | 289 | 291 | 302 | 312 | 222 | 230 | 169 | 177 | 237 | 245 | 200 | 206 | 249 | 249 | 94 | 104 | 199 | 201 | 79 | 85 | 263 | 263 | 190 | 194 |
| **Savagnin blanc B** | 231 | 237 | 243 | 257 | 251 | 251 | 188 | 188 | 236 | 238 | 241 | 273 | 151 | 151 | 188 | 194 | 244 | 250 | 253 | 263 | 126 | 132 | 139 | 163 | 203 | 215 | 213 | 217 | 201 | 207 | 200 | 208 | 248 | 248 | 156 | 166 | 203 | 237 | 153 | 165 | 289 | 295 | 312 | 316 | 230 | 230 | 165 | 169 | 237 | 237 | 200 | 206 | 247 | 249 | 94 | 104 | 201 | 201 | 79 | 85 | 263 | 263 | 180 | 194 |
| Schiava gentile N. | 235 | 237 | 247 | 249 | 241 | 243 | 180 | 184 | 238 | 256 | 253 | 273 | 141 | 155 | 192 | 200 | 238 | 258 | 253 | 257 | 139 | 155 | 159 | 193 | 203 | 215 | 209 | 217 | 191 | 207 | 208 | 210 | 248 | 248 | 156 | 166 | 203 | 217 | 159 | 181 | 291 | 295 | 310 | 316 | 230 | 230 | 159 | 165 | 237 | 237 | 206 | 206 | 247 | 249 | 94 | 94 | 201 | 201 | 79 | 81 | 261 | 263 | 190 | 192 |
| **Sciaccarello** | 225 | 227 | 239 | 247 | 241 | 243 | 182 | 188 | 238 | 260 | 253 | 273 | 133 | 133 | 194 | 204 | 244 | 244 | 257 | 269 | 132 | 139 | 137 | 159 | 203 | 215 | 209 | 209 | 191 | 207 | 200 | 210 | 248 | 254 | 166 | 170 | 217 | 217 | 153 | 163 | 289 | 295 | 302 | 316 | 218 | 218 | 165 | 169 | 237 | 237 | 200 | 206 | 247 | 247 | 94 | 108 | 201 | 205 | 79 | 81 | 263 | 263 | 180 | 188 |
| *Scimiscià B.* | 225 | 235 | 239 | 253 | 243 | 251 | 178 | 188 | 238 | 246 | 238 | 263 | 133 | 133 | 188 | 200 | 250 | 258 | 253 | 263 | 139 | 153 | 151 | 151 | 211 | 211 | 213 | 217 | 201 | 201 | 208 | 210 | 248 | 248 | 166 | 170 | 217 | 229 | 153 | 171 | 289 | 295 | 302 | 312 | 230 | 230 | 165 | 169 | 237 | 237 | 206 | 206 | 249 | 251 | 94 | 100 | 201 | 205 | 79 | 81 | 263 | 263 | 188 | 196 |
| Servavillano | 225 | 225 | 249 | 249 | 241 | 257 | 184 | 188 | 246 | 260 | 273 | 273 | 135 | 139 | 200 | 204 | 242 | 244 | 253 | 263 | 139 | 139 | 159 | 159 | 211 | 215 | 209 | 217 | 207 | 207 | 208 | 210 | 248 | 254 | 170 | 170 | 201 | 209 | 153 | 153 | 291 | 291 | 304 | 316 | 218 | 228 | 159 | 165 | 237 | 237 | 218 | 222 | 247 | 247 | 94 | 94 | 201 | 205 | 81 | 85 | 263 | 263 | 190 | 194 |
| Sgavetta N. | 231 | 239 | 239 | 247 | 241 | 257 | 184 | 184 | 236 | 262 | 241 | 253 | 133 | 135 | 196 | 196 | 242 | 242 | 265 | 265 | 139 | 153 | 137 | 159 | 211 | 215 | 217 | 217 | 191 | 191 | 190 | 210 | 242 | 248 | 166 | 170 | 211 | 217 | 153 | 163 | 289 | 291 | 302 | 316 | 206 | 230 | 159 | 169 | 237 | 237 | 200 | 200 | 247 | 253 | 90 | 94 | 199 | 205 | 81 | 85 | 263 | 263 | 174 | 180 |
| Slarina N. | 225 | 233 | 247 | 249 | 251 | 257 | 178 | 193 | 236 | 238 | 241 | 265 | 139 | 143 | 200 | 204 | 248 | 250 | 263 | 271 | 150 | 153 | 137 | 157 | 203 | 211 | 209 | 213 | 201 | 207 | 208 | 208 | 248 | 248 | 160 | 160 | 203 | 217 | 153 | 165 | 291 | 295 | 302 | 312 | 208 | 230 | 159 | 169 | 237 | 245 | 206 | 222 | 247 | 249 | 100 | 104 | 201 | 203 | 79 | 85 | 255 | 261 | 176 | 180 |
| Spergola B. (Vernaccia di Oristano B.) | 231 | 245 | 249 | 263 | 243 | 251 | 180 | 193 | 236 | 238 | 253 | 257 | 133 | 155 | 194 | 200 | 250 | 258 | 263 | 263 | 139 | 163 | 149 | 159 | 209 | 211 | 209 | 217 | 191 | 191 | 208 | 210 | 242 | 248 | 166 | 170 | 217 | 217 | 153 | 181 | 299 | 299 | 302 | 316 | 228 | 230 | 159 | 165 | 237 | 239 | 206 | 212 | 247 | 249 | 94 | 108 | 201 | 201 | 79 | 85 | 263 | 263 | 176 | 192 |
| *Syrah N.* | 225 | 231 | 239 | 239 | 243 | 243 | 188 | 190 | 220 | 230 | 241 | 273 | 133 | 133 | 188 | 194 | 244 | 250 | 253 | 294 | 126 | 150 | 139 | 143 | 211 | 215 | 209 | 215 | 191 | 207 | 200 | 210 | 248 | 264 | 166 | 170 | 217 | 217 | 153 | 165 | 291 | 295 | 310 | 316 | 226 | 228 | 159 | 177 | 241 | 245 | 200 | 218 | 249 | 253 | 90 | 104 | 199 | 201 | 85 | 85 | 261 | 263 | 182 | 190 |
| Tadone 1 (from Saluzzo) | 227 | 235 | 247 | 249 | 241 | 243 | 184 | 184 | 260 | 262 | 263 | 265 | 133 | 143 | 194 | 200 | 242 | 250 | 263 | 265 | 132 | 150 | 137 | 143 | 211 | 215 | 209 | 209 | 201 | 207 | 208 | 210 | 248 | 248 | 160 | 166 | 217 | 217 | 153 | 159 | 291 | 291 | 302 | 316 | 230 | 230 | 165 | 165 | 237 | 245 | 200 | 222 | 247 | 261 | 104 | 108 | 203 | 207 | 79 | 85 | 263 | 263 | 182 | 196 |
| Tadone 2 (from Saluzzo) | 227 | 235 | 249 | 261 | 241 | 241 | 184 | 193 | 238 | 262 | 265 | 273 | 139 | 143 | 198 | 200 | 242 | 250 | 265 | 265 | 150 | 150 | 143 | 163 | 209 | 211 | 209 | 213 | 207 | 207 | 208 | 208 | 248 | 248 | 160 | 166 | 217 | 217 | 153 | 181 | 291 | 299 | 302 | 316 | 218 | 230 | 159 | 165 | 237 | 237 | 206 | 222 | 247 | 261 | 100 | 104 | 201 | 207 | 79 | 79 | 255 | 263 | 176 | 196 |
| Teinturier ad acino rotondo | 235 | 237 | 239 | 247 | 251 | 257 | 188 | 193 | 230 | 236 | 253 | 273 | 133 | 151 | 194 | 196 | 244 | 250 | 253 | 296 | 126 | 139 | 139 | 159 | 211 | 215 | 209 | 213 | 191 | 207 | 200 | 208 | 248 | 254 | 156 | 166 | 201 | 237 | 153 | 181 | 291 | 295 | 312 | 316 | 230 | 230 | 165 | 165 | 237 | 237 | 206 | 206 | 253 | 253 | 94 | 108 | 201 | 201 | 79 | 81 | 263 | 263 | 184 | 190 |
| Teinturier du Cher | 237 | 237 | 239 | 243 | 241 | 251 | 188 | 188 | 230 | 238 | 273 | 273 | 137 | 151 | 188 | 194 | 244 | 254 | 253 | 253 | 126 | 126 | 139 | 139 | 203 | 215 | 209 | 213 | 201 | 207 | 200 | 208 | 248 | 248 | 156 | 156 | 201 | 237 | 153 | 153 | 295 | 295 | 312 | 316 | 208 | 230 | 165 | 165 | 237 | 237 | 206 | 222 | 247 | 253 | 94 | 94 | 201 | 201 | 81 | 85 | 263 | 263 | 184 | 194 |
| Teroldego N. | 225 | 227 | 239 | 247 | 241 | 243 | 178 | 184 | 230 | 238 | 241 | 263 | 137 | 155 | 194 | 194 | 242 | 254 | 253 | 253 | 153 | 163 | 149 | 163 | 211 | 215 | 209 | 215 | 191 | 195 | 200 | 210 | 242 | 248 | 166 | 170 | 203 | 217 | 153 | 181 | 295 | 299 | 302 | 316 | 226 | 230 | 159 | 177 | 237 | 239 | 212 | 222 | 247 | 253 | 94 | 94 | 201 | 201 | 79 | 81 | 263 | 263 | 182 | 196 |
| *Terrano N.* | 225 | 227 | 247 | 249 | 243 | 257 | 188 | 188 | 220 | 236 | 251 | 273 | 135 | 155 | 192 | 194 | 238 | 250 | 253 | 253 | 132 | 153 | 151 | 163 | 209 | 211 | 213 | 217 | 191 | 201 | 200 | 208 | 248 | 254 | 160 | 170 | 201 | 203 | 153 | 159 | 289 | 295 | 302 | 316 | 226 | 232 | 165 | 169 | 237 | 237 | 206 | 222 | 247 | 253 | 94 | 114 | 203 | 203 | 79 | 85 | 255 | 263 | 184 | 194 |
| Timorasso B. | 225 | 225 | 247 | 247 | 241 | 243 | 178 | 178 | 236 | 260 | 253 | 257 | 133 | 133 | 196 | 204 | 250 | 258 | 263 | 263 | 126 | 153 | 141 | 163 | 215 | 215 | 209 | 209 | 191 | 215 | 210 | 210 | 248 | 254 | 170 | 170 | 209 | 217 | 153 | 165 | 289 | 291 | 302 | 316 | 218 | 222 | 165 | 169 | 237 | 237 | 206 | 218 | 247 | 247 | 90 | 94 | 201 | 201 | 81 | 85 | 263 | 263 | 180 | 194 |
| Trebbianino di Tortona (presumed) | 225 | 231 | 247 | 253 | 241 | 251 | 180 | 184 | 270 | 270 | 273 | 273 | 133 | 151 | 200 | 204 | 238 | 258 | 263 | 265 | 139 | 150 | 159 | 163 | 211 | 211 | 209 | 213 | 201 | 207 | 190 | 208 | 248 | 248 | 160 | 170 | 201 | 217 | 153 | 165 | 291 | 295 | 316 | 316 | 218 | 228 | 165 | 169 | 237 | 237 | 206 | 212 | 247 | 247 | 94 | 94 | 201 | 205 | 81 | 81 | 263 | 263 | 180 | 190 |
| *Trebbiano toscano B.* | 225 | 231 | 249 | 253 | 243 | 257 | 178 | 182 | 246 | 250 | 251 | 273 | 133 | 143 | 194 | 200 | 244 | 250 | 263 | 265 | 132 | 150 | 137 | 163 | 209 | 211 | 209 | 209 | 203 | 207 | 200 | 210 | 242 | 248 | 160 | 170 | 209 | 217 | 149 | 153 | 295 | 295 | 302 | 316 | 208 | 228 | 159 | 165 | 237 | 237 | 200 | 206 | 249 | 261 | 94 | 100 | 201 | 207 | 79 | 85 | 255 | 263 | 184 | 190 |
| Unknown black (from Fresonara) | 225 | 235 | 249 | 249 | 241 | 251 | 178 | 188 | 236 | 236 | 253 | 273 | 133 | 133 | 200 | 202 | 250 | 258 | 243 | 243 | 132 | 150 | 143 | 163 | 211 | 211 | 207 | 217 | 201 | 203 | 190 | 190 | 242 | 242 | 170 | 170 | 209 | 227 | 171 | 181 | 291 | 295 | 302 | 312 | 206 | 208 | 165 | 165 | 221 | 237 | 196 | 222 | 247 | 249 | 94 | 94 | 201 | 207 | 79 | 85 | 263 | 263 | 190 | 192 |
| Unknown black (from Pomaretto) | 231 | 245 | 247 | 249 | 243 | 251 | 178 | 188 | 230 | 238 | 273 | 273 | 151 | 155 | 186 | 200 | 244 | 258 | 251 | 251 | 139 | 153 | 137 | 159 | 209 | 211 | 209 | 209 | 207 | 207 | 208 | 210 | 248 | 254 | 166 | 170 | 217 | 217 | 165 | 181 | 291 | 299 | 302 | 316 | 218 | 230 | 165 | 177 | 237 | 237 | 206 | 222 | 247 | 261 | 90 | 104 | 201 | 201 | 81 | 81 | 263 | 263 | 188 | 196 |
| Unknown black (from Sale) | 231 | 239 | 247 | 249 | 241 | 257 | 184 | 188 | 260 | 260 | 241 | 273 | 139 | 143 | 196 | 196 | 244 | 250 | 253 | 263 | 139 | 153 | 137 | 159 | 211 | 215 | 209 | 217 | 191 | 207 | 208 | 210 | 248 | 248 | 168 | 170 | 209 | 217 | 163 | 165 | 291 | 295 | 302 | 316 | 206 | 208 | 159 | 165 | 221 | 237 | 200 | 222 | 247 | 247 | 90 | 108 | 201 | 205 | 79 | 85 | 263 | 263 | 174 | 180 |
| Unknown black (from Spineto) | 225 | 239 | 247 | 255 | 241 | 257 | 188 | 193 | 260 | 270 | 241 | 265 | 143 | 155 | 194 | 204 | 250 | 250 | 253 | 263 | 126 | 150 | 141 | 157 | 211 | 215 | 209 | 213 | 191 | 201 | 190 | 208 | 248 | 248 | 164 | 170 | 201 | 217 | 153 | 153 | 295 | 299 | 316 | 316 | 208 | 228 | 159 | 165 | 239 | 239 | 206 | 206 | 247 | 247 | 94 | 100 | 201 | 203 | 79 | 85 | 263 | 263 | 180 | 190 |
| Unknown black (from Tortona) | 225 | 235 | 247 | 249 | 241 | 257 | 180 | 184 | 236 | 262 | 253 | 257 | 135 | 139 | 196 | 200 | 250 | 258 | 265 | 265 | 150 | 153 | 137 | 139 | 211 | 211 | 209 | 213 | 191 | 191 | 190 | 208 | 242 | 248 | 166 | 170 | 209 | 217 | 153 | 163 | 295 | 299 | 302 | 316 | 206 | 230 | 159 | 159 | 237 | 237 | 212 | 222 | 247 | 247 | 90 | 94 | 201 | 201 | 79 | 85 | 263 | 263 | 176 | 192 |
| Unknown black 1 (from Abbadia L.) | 227 | 231 | 247 | 249 | 241 | 243 | 178 | 188 | 238 | 260 | 241 | 253 | 137 | 155 | 188 | 200 | 244 | 250 | 263 | 294 | 126 | 153 | 137 | 139 | 195 | 211 | 213 | 217 | 201 | 203 | 190 | 208 | 248 | 248 | 160 | 166 | 201 | 217 | 143 | 165 | 295 | 299 | 302 | 302 | 228 | 228 | 165 | 165 | 237 | 237 | 206 | 206 | 247 | 249 | 94 | 116 | 201 | 201 | 81 | 85 | 263 | 267 | 180 | 184 |
| Unknown black 2 (from Abbadia L.) | 225 | 245 | 249 | 253 | 243 | 257 | 180 | 184 | 260 | 260 | 241 | 273 | 143 | 145 | 190 | 192 | 250 | 258 | 253 | 275 | 150 | 153 | 139 | 163 | 211 | 211 | 213 | 217 | 191 | 207 | 190 | 208 | 248 | 254 | 164 | 170 | 209 | 217 | 153 | 163 | 293 | 295 | 302 | 316 | 206 | 228 | 165 | 165 | 237 | 237 | 196 | 200 | 249 | 253 | 90 | 90 | 201 | 203 | 79 | 79 | 263 | 263 | 174 | 180 |
| Unknown white (from Paderna) | 225 | 235 | 247 | 253 | 257 | 259 | 193 | 193 | 238 | 260 | 263 | 273 | 135 | 155 | 194 | 196 | 250 | 250 | 253 | 253 | 139 | 139 | 159 | 163 | 211 | 211 | 213 | 217 | 191 | 207 | 200 | 208 | 254 | 254 | 166 | 170 | 209 | 217 | 165 | 181 | 291 | 295 | 302 | 316 | 228 | 230 | 159 | 169 | 237 | 237 | 206 | 206 | 247 | 247 | 94 | 100 | 201 | 201 | 79 | 85 | 261 | 263 | 180 | 192 |
| Uva 'd Batita | 225 | 231 | 247 | 247 | 251 | 257 | 188 | 193 | 236 | 260 | 273 | 273 | 135 | 151 | 186 | 204 | 246 | 250 | 251 | 253 | 139 | 153 | 159 | 163 | 215 | 215 | 209 | 209 | 193 | 207 | 210 | 210 | 248 | 254 | 166 | 170 | 203 | 217 | 165 | 171 | 291 | 295 | 312 | 316 | 208 | 222 | 165 | 165 | 237 | 237 | 200 | 206 | 247 | 249 | 90 | 94 | 201 | 203 | 81 | 85 | 255 | 263 | 176 | 180 |
| Uva delle cascine | 233 | 239 | 247 | 249 | 257 | 257 | 180 | 184 | 236 | 262 | 253 | 273 | 143 | 143 | 196 | 204 | 242 | 258 | 263 | 265 | 139 | 150 | 139 | 153 | 215 | 215 | 217 | 217 | 191 | 207 | 210 | 210 | 248 | 248 | 160 | 170 | 217 | 217 | 163 | 165 | 291 | 299 | 316 | 316 | 208 | 226 | 153 | 159 | 237 | 237 | 200 | 218 | 247 | 249 | 94 | 108 | 201 | 201 | 79 | 85 | 263 | 263 | 180 | 190 |
| Uva rara N. | 235 | 239 | 247 | 249 | 251 | 257 | 184 | 188 | 236 | 260 | 253 | 273 | 133 | 143 | 196 | 200 | 242 | 244 | 265 | 296 | 150 | 153 | 137 | 139 | 211 | 211 | 209 | 217 | 191 | 207 | 208 | 208 | 248 | 248 | 168 | 170 | 209 | 209 | 153 | 181 | 289 | 295 | 302 | 316 | 226 | 232 | 165 | 165 | 237 | 237 | 196 | 200 | 247 | 253 | 90 | 94 | 201 | 201 | 85 | 85 | 263 | 263 | 174 | 180 |
| Uvalino N. | 235 | 245 | 247 | 255 | 257 | 257 | 178 | 188 | 230 | 260 | 251 | 273 | 135 | 143 | 196 | 204 | 248 | 250 | 251 | 253 | 126 | 153 | 137 | 141 | 209 | 215 | 209 | 217 | 201 | 207 | 190 | 208 | 248 | 248 | 164 | 166 | 203 | 217 | 153 | 153 | 295 | 299 | 302 | 316 | 208 | 228 | 159 | 159 | 237 | 245 | 206 | 222 | 247 | 251 | 94 | 94 | 201 | 205 | 79 | 85 | 255 | 263 | 176 | 180 |
| Verdea B. | 233 | 239 | 247 | 247 | 241 | 257 | 178 | 188 | 238 | 262 | 251 | 273 | 133 | 133 | 194 | 204 | 244 | 248 | 263 | 265 | 139 | 139 | 159 | 159 | 209 | 209 | 209 | 209 | 191 | 207 | 208 | 208 | 248 | 254 | 160 | 170 | 203 | 217 | 153 | 163 | 289 | 295 | 302 | 316 | 206 | 228 | 159 | 165 | 237 | 237 | 206 | 206 | 247 | 247 | 100 | 100 | 201 | 205 | 79 | 79 | 263 | 263 | 184 | 190 |
| Vermentino B. | 233 | 237 | 249 | 249 | 243 | 251 | 178 | 180 | 238 | 246 | 251 | 257 | 133 | 151 | 200 | 204 | 248 | 258 | 263 | 263 | 132 | 132 | 137 | 137 | 203 | 211 | 209 | 213 | 201 | 205 | 190 | 210 | 242 | 248 | 156 | 160 | 201 | 209 | 149 | 159 | 295 | 299 | 302 | 312 | 226 | 230 | 169 | 169 | 237 | 237 | 206 | 206 | 247 | 249 | 94 | 100 | 201 | 205 | 81 | 85 | 263 | 263 | 176 | 184 |
| Vermentino nero N. | 225 | 239 | 239 | 253 | 257 | 257 | 184 | 188 | 236 | 262 | 253 | 263 | 135 | 143 | 188 | 192 | 238 | 250 | 249 | 249 | 132 | 163 | 137 | 149 | 209 | 211 | 209 | 213 | 191 | 191 | 208 | 208 | 248 | 254 | 170 | 170 | 209 | 217 | 153 | 163 | 291 | 299 | 302 | 316 | 206 | 228 | 165 | 169 | 237 | 239 | 200 | 212 | 249 | 261 | 94 | 100 | 201 | 205 | 81 | 85 | 263 | 263 | 174 | 196 |
| *Vernaccia di S. Gimignano B.* | 225 | 225 | 239 | 249 | 243 | 243 | 182 | 188 | 246 | 260 | 251 | 273 | 135 | 143 | 188 | 190 | 238 | 244 | 263 | 275 | 132 | 139 | 137 | 159 | 211 | 211 | 209 | 213 | 191 | 207 | 208 | 208 | 254 | 264 | 170 | 170 | 217 | 227 | 153 | 165 | 291 | 295 | 316 | 316 | 208 | 228 | 159 | 169 | 221 | 237 | 196 | 218 | 249 | 261 | 90 | 100 | 201 | 205 | 79 | 85 | 255 | 263 | 180 | 194 |
| Vespolina N. | 235 | 239 | 247 | 249 | 243 | 257 | 184 | 184 | 238 | 262 | 241 | 263 | 143 | 155 | 196 | 200 | 242 | 250 | 265 | 265 | 126 | 139 | 141 | 159 | 211 | 211 | 213 | 217 | 191 | 191 | 208 | 210 | 248 | 248 | 166 | 170 | 217 | 217 | 163 | 171 | 291 | 295 | 302 | 316 | 222 | 232 | 159 | 159 | 237 | 237 | 206 | 222 | 247 | 249 | 94 | 90 | 201 | 205 | 79 | 85 | 263 | 263 | 174 | 196 |
| Vien de Nus N. | 225 | 235 | 239 | 247 | 243 | 243 | 188 | 188 | 260 | 260 | 241 | 253 | 133 | 155 | 194 | 194 | 246 | 250 | 253 | 263 | 150 | 155 | 139 | 143 | 209 | 215 | 213 | 217 | 191 | 203 | 208 | 210 | 248 | 254 | 166 | 170 | 201 | 217 | 153 | 181 | 289 | 299 | 310 | 316 | 208 | 222 | 165 | 169 | 237 | 237 | 206 | 222 | 247 | 249 | 94 | 94 | 201 | 203 | 79 | 81 | 263 | 263 | 176 | 184 |
| Viognier B. | 225 | 231 | 239 | 249 | 241 | 243 | 184 | 190 | 220 | 246 | 263 | 273 | 133 | 139 | 188 | 200 | 250 | 250 | 263 | 294 | 139 | 150 | 143 | 159 | 211 | 211 | 209 | 213 | 203 | 207 | 208 | 210 | 248 | 248 | 166 | 170 | 201 | 217 | 153 | 153 | 291 | 295 | 302 | 302 | 222 | 230 | 159 | 165 | 237 | 237 | 206 | 218 | 247 | 249 | 90 | 104 | 199 | 201 | 85 | 85 | 261 | 263 | 184 | 194 |
| Vurpin (false) | 235 | 239 | 247 | 249 | 243 | 257 | 178 | 184 | 248 | 260 | 273 | 273 | 133 | 135 | 188 | 194 | 236 | 242 | 253 | 294 | 126 | 153 | 137 | 141 | 211 | 211 | 209 | 217 | 207 | 207 | 190 | 210 | 248 | 248 | 160 | 166 | 201 | 217 | 153 | 171 | 291 | 295 | 310 | 316 | 218 | 228 | 159 | 165 | 237 | 237 | 200 | 206 | 247 | 253 | 94 | 100 | 201 | 201 | 85 | 85 | 263 | 267 | 176 | 192 |
| Zanello | 225 | 231 | 247 | 247 | 241 | 257 | 184 | 184 | 260 | 270 | 241 | 273 | 135 | 139 | 194 | 196 | 242 | 250 | 253 | 263 | 139 | 153 | 137 | 159 | 211 | 211 | 209 | 217 | 191 | 207 | 208 | 210 | 248 | 248 | 170 | 172 | 201 | 201 | 141 | 153 | 291 | 299 | 312 | 316 | 218 | 232 | 159 | 159 | 237 | 239 | 206 | 222 | 247 | 247 | 94 | 104 | 201 | 201 | 85 | 85 | 261 | 263 | 180 | 194 |
| Zibibbo B. (Muscat of Alexandria) | 227 | 231 | 249 | 251 | 251 | 251 | 178 | 193 | 246 | 270 | 265 | 273 | 133 | 149 | 186 | 204 | 246 | 254 | 253 | 263 | 126 | 126 | 139 | 141 | 215 | 223 | 213 | 213 | 191 | 207 | 190 | 210 | 254 | 264 | 160 | 168 | 205 | 217 | 141 | 165 | 291 | 295 | 312 | 316 | 230 | 230 | 159 | 169 | 237 | 237 | 206 | 218 | 247 | 249 | 94 | 100 | 199 | 207 | 79 | 79 | 263 | 263 | 188 | 192 |

Table S5 - List of the resulting kinship pairs putative parent/offspring and related statistics based on nSSR and SNPs. The cultivars used as references for parent/offspring relations are in bold. Teorethical values for IBD coefficients, k0, k1, k2 and kinship are for PO relationships 0, 1, 0 and 0.25 respectively. * indicates that the other parent is missing from this dataset.

| | | | nSSR | | | SNP | | | | | |
|---|---|---|---|---|---|---|---|---|---|---|---|
| **Cultivar 1** | **Cultivar 2** | **PO relationship included in a family trio (2 parents/ 1 offspring)** | **Proportion of matching loci** | **LOD score** | **Mismatching loci** | **Mendelian errors** | **Mendelian errors (%)** | **k0** | **k1** | **k2** | **kinship** |
| Albana B. | Garganega B. | | 29/32 | 1,22E+15 | VvMD7, VMC2F10, VMC4C6 | 14 | 0.26 | 0.02 | 0.98 | 0 | 0.25 |
| Aleatico (false, from Paderna) | Moscato bianco B. | | 31/32 | 1,64E+15 | | | | | | | |
| Aleatico N. | Moscato bianco B. | | 32/32 | 1,97E+15 | | | | | | | |
| Avanà N. | Bianchetto (from Saluzzo) | | 32/32 | 1,51E+15 | | 8 | 0.14 | 0.01 | 0.92 | 0.07 | 0.27 |
| Avarenchetto | Bottagera (false) | yes | 32/32 | 1,12E+15 | | | | | | | |
| Avarenchetto | Gouais blanc (Liseiret B.) | yes | 32/32 | 2,04E+15 | | | | | | | |
| Avarengo N. | Barbera ciarìa | | 31/32 | 7,51E+14 | VvMD36 (null allele) | | | | | | |
| Avarengo N. | Gris | yes | 32/32 | 1,63E+15 | | | | | | | |
| Avarengo N. | Grisa (from Cumiana) | yes | 30/32 | 1,01E+15 | VvMD36 (null allele), VMC1E8 | 10 | 0.18 | 0.01 | 0.9 | 0.08 | 0.27 |
| Avarengo N. | Lacrima Cristi | yes | 32/32 | 1,43E+15 | | | | | | | |
| Balsamina (from Reggio Emilia area) | Coccalona nera (presumed, Rohrtraube blaurot) | | 32/32 | 1,73E+15 | | | | | | | |
| Baratuciat B. | Cascarolo | | 31/32 | 2,09E+15 | VvMD6 (null allele) | 8 | 0.16 | 0 | 1 | 0 | 0.25 |
| Barbassese | Timorasso B. | | 32/32 | 1,80E+15 | | 8 | 0.15 | 0.01 | 0.99 | 0 | 0.25 |
| Barbera bianca B. | Crovin (from Perti) | yes | 32/32 | 2,86E+15 | | 12 | 0.23 | 0.02 | 0.98 | 0 | 0.25 |
| Barbera bianca B. | Timorasso B. | yes | 32/32 | 1,14E+15 | | 11 | 0.22 | 0 | 1 | 0 | 0.25 |
| Barbera di Patrunat | Moscato bianco B. | yes | 32/32 | 2,04E+15 | | | | | | | |
| Barbera di Patrunat | Neretto duro (Balau) | yes | 31/32 | 9,24E+14 | VvMD36 (null allele) | | | | | | |
| Barbera N. | Coccalona nera (presumed, Rohrtraube blaurot) | | 32/32 | 1,95E+15 | | 14 | 0.27 | 0 | 1 | 0 | 0.25 |
| Barbera N. | Unknown black (from Fresonara) | yes | 31/32 | 1,46E+15 | VvMD36 | | | | | | |
| Barberùn | Bottagera (false) | | 32/32 | 1,12E+15 | | | | | | | |
| Barbrassa | Galletta | | 32/32 | 1,37E+15 | | | | | | | |
| Berla grossa | Gouais blanc (Liseiret B.) | yes | 32/32 | 2,13E+15 | | | | | | | |
| Berla grossa | Lambrusca vittona | yes | 32/32 | 1,71E+15 | | | | | | | |
| Bianchetta (from Frascata) | Dolcetto bianco | yes | 32/32 | 1,49E+15 | | | | | | | |
| Bianchetta (from Frascata) | Patlassa (long internodes, presumed) | yes | 32/32 | 2,23E+15 | | | | | | | |
| Bianchetta (from Frascata) | Uva delle cascine | yes | 32/32 | 2,03E+15 | | | | | | | |
| Bianchetto (from Saluzzo) | Bubbierasco | yes | 32/32 | 2,26E+15 | | 12 | 0.21 | 0.02 | 0.89 | 0.09 | 0.27 |
| Bibiola | Moissan | | 32/32 | 1,56E+15 | | 13 | 0.24 | 0.02 | 0.96 | 0.02 | 0.25 |
| Biestro (presumed) | Dolcetto bianco | yes | 32/32 | 2,19E+15 | | | | | | | |
| Biestro (presumed) | Moissan | yes | 31/32 | 9,58E+14 | VvMD36 (null allele) | | | | | | |
| Bonarda N. | Moissan | | 32/32 | 1,93E+15 | | 9 | 0.17 | 0 | 1 | 0 | 0.25 |
| Bonardina | Croatina N. | yes | 31/32 | 1,37E+15 | VvMD36 | 10 | 0.18 | 0.01 | 0.95 | 0.03 | 0.26 |
| Bonardina | Malvasia aromatica di Parma (Malvasia Casalini B.) | yes | 32/32 | 1,70E+15 | | 13 | 0.24 | 0.02 | 0.97 | 0.01 | 0.25 |
| Bonda N. | Moissan | | 32/32 | 1,47E+15 | | | | | | | |
| Bonda N. | Uva 'd Batita | | 32/32 | 2,12E+15 | | | | | | | |
| Bordò | Moscato bianco B. | | 32/32 | 1,64E+15 | | 15 | 0.28 | 0 | 1 | 0 | 0.25 |
| Bottagera (false) | Bressana | | 31/32 | 1,03E+15 | VMC5G8 | 12 | 0.23 | 0 | 1 | 0 | 0.25 |
| Bottagera (false) | Cardìn | | 32/32 | 1,11E+15 | | 11 | 0.21 | 0.01 | 0.98 | 0.01 | 0.25 |
| Bottagera (false) | Coccalona nera (presumed, Rohrtraube blaurot) | | 32/32 | 1,40E+15 | | 5 | 0.09 | 0 | 0.99 | 0.01 | 0.25 |
| Bottagera (false) | Galletta | | 32/32 | 1,32E+15 | | | | | | | |
| Bottagera (false) | Giamellotto (false) | | 32/32 | 9,84E+14 | | | | | | | |
| Bottagera (false) | Grisa (from Cumiana) | yes | 32/32 | 1,38E+15 | | 12 | 0.22 | 0.01 | 0.98 | 0.01 | 0.25 |
| Bottagera (false) | Lambrusca vittona | | 32/32 | 7,76E+14 | | | | | | | |
| Bottagera (false) | Mossano (from Canavese area) | yes | 32/32 | 1,45E+15 | | | | | | | |
| Bottagera (false) | Plassa N. | | 32/32 | 9,38E+14 | | 9 | 0.17 | 0.01 | 0.99 | 0 | 0.25 |
| Bottagera (false) | Servavillano | | 32/32 | 1,36E+15 | | | | | | | |
| Bottagera (false) | Unknown white (from Paderna) | | 32/32 | 1,10E+15 | | 11 | 0.2 | 0.01 | 0.97 | 0.02 | 0.25 |
| Bracciola nera N. | Rossara N. (Schiava N.) | | 32/32 | 1,43E+15 | | | | | | | |
| Brachetto (false, from Nizza M.to) | Moscato bianco B. | | 32/32 | 1,62E+15 | | 16 | 0.29 | 0.02 | 0.97 | 0.01 | 0.25 |
| Brachetto Migliardi | Lambrusca di Alessandria N. | yes | 32/32 | 1,42E+15 | | 14 | 0.26 | 0.02 | 0.98 | 0 | 0.25 |
| Brachetto Migliardi | Malvasia aromatica di Parma (Malvasia Casalini B.) | yes | 32/32 | 1,18E+15 | | 8 | 0.15 | 0 | 1 | 0 | 0.25 |
| Brachetto N. | Moscato bianco B. | | 32/32 | 1,90E+15 | | 10 | 0.18 | 0 | 1 | 0 | 0.25 |
| Bragat rosa N. | Moscato bianco precoce | | 32/32 | 1,83E+15 | | 12 | 0.21 | 0.01 | 0.94 | 0.04 | 0.26 |
| Brugnola | Nebbiolo N. | | 32/32 | 1,40E+15 | | 6 | 0.12 | 0.01 | 0.94 | 0.05 | 0.26 |
| Bubbierasco | Nebbiolo N. | yes | 32/32 | 1,87E+15 | | 5 | 0.1 | 0.01 | 0.92 | 0.07 | 0.27 |
| **Cabernet franc N.** | **Cabernet sauvignon N.** | yes | 32/32 | 3,41E+15 | | 14 | 0.27 | 0.02 | 0.97 | 0.01 | 0.25 |
| **Cabernet sauvignon N.** | **Sauvignon B.** | yes | 32/32 | 2,52E+15 | | | | | | | |
| Cagambraga | Chiavennasca bianca | | 32/32 | 1,33E+15 | | 3 | 0.06 | 0 | 1 | 0 | 0.25 |
| Cavazzina | Coccalona nera (presumed, Rohrtraube blaurot) | | 32/32 | 1,33E+15 | | | | | | | |
| Chardonnay B. | **Charmont B** | yes | 32/32 | 2,53E+15 | | 18 | 0.33 | 0.02 | 0.92 | 0.06 | 0.26 |
| Chardonnay B. | Gouais blanc (Liseiret B.) | yes | 32/32 | 1,71E+15 | | 21 | 0.41 | 0 | 1 | 0 | 0.25 |
| Chardonnay B. | **Pinot nero N. (Pinot noir)** | yes | 32/32 | 2,79E+15 | | 7 | 0.13 | 0 | 0.94 | 0.05 | 0.26 |
| **Charmont B** | **Chasselas blanc B.** | yes | 32/32 | 1,15E+15 | | 6 | 0.11 | 0.01 | 0.85 | 0.14 | 0.28 |
| **Chasselas blanc B.** | Orange muscat (M. fleur d'oranger) | yes | 32/32 | 1,16E+15 | | | | | | | |
| Chatus N. | Lambrusca pignata | yes | 32/32 | 1,56E+15 | | | | | | | |
| Chatus N. | Nebbiolo di Dronero (false) | yes | 32/32 | 1,97E+15 | | 13 | 0.24 | 0.02 | 0.94 | 0.04 | 0.26 |
| Chatus N. | Nebbiolo gabardin | yes | 32/32 | 2,51E+15 | | | | | | | |
| Chatus N. | Unknown black 2 (from Abbadia L.) | | 32/32 | 2,11E+15 | | 12 | 0.23 | 0.02 | 0.97 | 0.01 | 0.25 |
| Chiavennasca bianca | Rossola (false) | | 32/32 | 1,44E+15 | | 3 | 0.06 | 0 | 1 | 0 | 0.25 |
| Chiavennasca bianca | Tadone 2 (from Saluzzo) | | 31/32 | 9,27E+14 | VvMD36 | 1 | 0.02 | 0 | 1 | 0 | 0.25 |
| Chiavennasca bianca | Unknown black (from Pomaretto) | yes | 31/32 | 9,09E+14 | VvMD36 | 3 | 0.06 | 0 | 0.93 | 0.07 | 0.27 |
| Citronino | Spergola B. (Vernaccia di Oristano B.) | | 31/32 | 1,10E+15 | VMC5A1 | 8 | 0.15 | 0.01 | 0.96 | 0.03 | 0.26 |
| Coccalona bianca | Malvasia bianca (from Vignale) | yes | 32/32 | 1,88E+15 | | | | | | | |
| Coccalona nera (presumed, Rohrtraube blaurot) | Crova (elliptic berry) (Uva crova) | | 32/32 | 1,79E+15 | | | | | | | |
| Coccalona nera (presumed, Rohrtraube blaurot) | Crovaro | | 32/32 | 2,11E+15 | | | | | | | |
| Coccalona nera (presumed, Rohrtraube blaurot) | Durasa N. | yes | 32/32 | 2,34E+15 | | 9 | 0.17 | 0.01 | 0.97 | 0.03 | 0.25 |
| Coccalona nera (presumed, Rohrtraube blaurot) | Lambrusco del pellegrino N. (L. di Fiorano) | | 32/32 | 1,80E+15 | | | | | | | |
| Coccalona nera (presumed, Rohrtraube blaurot) | Moradella N. | | 32/32 | 2,15E+15 | | 10 | 0.18 | 0.01 | 0.9 | 0.09 | 0.27 |
| Coccalona nera (presumed, Rohrtraube blaurot) | Moscato nero (from Borbera valley) | yes | 32/32 | 1,57E+15 | | | | | | | |
| Coccalona nera (presumed, Rohrtraube blaurot) | Moscato nero di Acqui N. | yes | 32/32 | 1,35E+15 | | 14 | 0.27 | 0 | 1 | 0 | 0.25 |
| Coccalona nera (presumed, Rohrtraube blaurot) | Parmesana | | 32/32 | 1,71E+15 | | | | | | | |
| Coccalona nera (presumed, Rohrtraube blaurot) | Picolit (false) | | 32/32 | 1,85E+15 | | | | | | | |
| Coccalona nera (presumed, Rohrtraube blaurot) | Rastajola | | 32/32 | 1,89E+15 | | 14 | 0.25 | 0.02 | 0.85 | 0.13 | 0.28 |
| Coccalona nera (presumed, Rohrtraube blaurot) | Riesling italico B. | | 32/32 | 1,56E+15 | | 15 | 0.3 | 0 | 1 | 0 | 0.25 |
| Coccalona nera (presumed, Rohrtraube blaurot) | Sgavetta N. | | 32/32 | 1,91E+15 | | | | | | | |
| Coccalona nera (presumed, Rohrtraube blaurot) | Unknown black (from Sale) | | 32/32 | 1,31E+15 | | 12 | 0.23 | 0 | 1 | 0 | 0.25 |
| Coccalona nera (presumed, Rohrtraube blaurot) | Unknown black (from Tortona) | | 32/32 | 1,50E+15 | | | | | | | |
| Coccalona nera (presumed, Rohrtraube blaurot) | Unknown black 1 (from Abbadia L.) | | 32/32 | 1,58E+15 | | | | | | | |
| Coccalona nera (presumed, Rohrtraube blaurot) | Uva rara N. | | 32/32 | 1,58E+15 | | 11 | 0.21 | 0 | 1 | 0 | 0.25 |
| Coccalona nera (presumed, Rohrtraube blaurot) | Vespolina N. | yes | 32/32 | 1,75E+15 | | 13 | 0.25 | 0.02 | 0.98 | 0 | 0.25 |
| Cortese B. | Unknown black (from Fresonara) | yes | 31/32 | 2,75E+15 | VvMD36 | | | | | | |
| Croatina N. | Primaticcia | yes | 32/32 | 1,50E+15 | | | | | | | |
| Croatina N. | Ruché N. | yes | 32/32 | 1,85E+15 | | 14 | 0.25 | 0.02 | 0.96 | 0.02 | 0.25 |
| Croetto | Moissan | | 30/32 | 7,02E+14 | VvMD36 (null allele), VvMD26 | | | | | | |
| Crovin (from Perti) | Lambrusca di Alessandria N. | yes | 32/32 | 2,24E+15 | | 12 | 0.23 | 0 | 1 | 0 | 0.25 |
| Dolcetto bianco | Dolcetto N. | yes | 32/32 | 1,69E+15 | | 4 | 0.07 | 0 | 0.98 | 0.01 | 0.25 |
| Dolcetto bianco | Moscato bianco precoce | yes | 32/32 | 1,82E+15 | | 7 | 0.12 | 0.01 | 0.91 | 0.08 | 0.27 |
| Dolcetto N. | Feral accession | yes | 32/32 | 2,41E+15 | | | | | | | |
| Dolcetto N. | Moissan | yes | 32/32 | 1,96E+15 | | 11 | 0.21 | 0.01 | 0.99 | 0 | 0.25 |
| Doux d'Henry N. | Lacrima Cristi | yes | 32/32 | 8,80E+14 | | | | | | | |
| Doux d'Henry N. | Lambrusca pignata | yes | 32/32 | 1,77E+15 | | | | | | | |
| Doux d'Henry N. | Moissan | | 32/32 | 1,45E+15 | | 7 | 0.13 | 0 | 1 | 0 | 0.25 |
| Doux d'Henry N. | Nebbiolo di Dronero (false) | yes | 32/32 | 1,47E+15 | | 9 | 0.16 | 0.01 | 0.97 | 0.02 | 0.25 |
| Durasa N. | Marzemino N. | yes | 31/32 | 1,06E+15 | VMC4C6 | 8 | 0.16 | 0.01 | 0.97 | 0.02 | 0.25 |
| Feral accession | Riesling italico B. | yes | 32/32 | 2,06E+15 | | | | | | | |
| Freisa N. | Malvasia nera lunga N. | yes | 32/32 | 1,51E+15 | | 6 | 0.11 | 0.01 | 0.89 | 0.1 | 0.27 |
| Freisa N. | Nebbiolo N. | | 32/32 | 2,24E+15 | | 10 | 0.19 | 0.02 | 0.97 | 0.02 | 0.25 |
| Fortana N. | Lambrusco Maestri N. | | 32/32 | 2,79E+15 | | | | | | | |
| Galletto | Moissan | | 32/32 | 1,28E+15 | | | | | | | |
| Gamay N. | **Garanoir N.** | yes* | 32/32 | 2,12E+15 | | 3 | 0.05 | 0 | 0.81 | 0.19 | 0.3 |
| Gamay N. | Gouais blanc (Liseiret B.) | yes | 32/32 | 2,10E+15 | | 14 | 0.27 | 0 | 1 | 0 | 0.25 |
| Gamay N. | **Pinot nero N. (Pinot noir)** | yes | 32/32 | 1,75E+15 | | 7 | 0.14 | 0 | 1 | 0 | 0.25 |
| Garganega B. | Trebbiano toscano B. | | 32/32 | 2,77E+15 | | 10 | 0.18 | 0.01 | 0.98 | 0.01 | 0.25 |
| Gouais blanc (Liseiret B.) | Riesling B. | | 32/32 | 2,03E+15 | | | | | | | |
| Gouais blanc (Liseiret B.) | Rossara N. (Schiava N.) | | 32/32 | 1,95E+15 | | 17 | 0.32 | 0.02 | 0.97 | 0.01 | 0.25 |
| Grandurey | Moissan | | 31/32 | 1,18E+15 | VvMD36 (null allele) | | | | | | |
| Grec rouge | Tadone 1 (from Saluzzo) | yes | 32/32 | 1,79E+15 | | 9 | 0.16 | 0 | 1 | 0 | 0.25 |
| Gris | Moissan | yes | 32/32 | 1,82E+15 | | | | | | | |
| **Heptakilo** | Zibibbo B. (Muscat of Alexandria) | yes | 32/32 | 3,39E+15 | | | | | | | |
| Lambrusca di Alessandria N. | Malvasia di Casorzo N. | yes | 32/32 | 3,12E+15 | | 17 | 0.31 | 0.02 | 0.94 | 0.04 | 0.25 |
| Lambrusca di Alessandria N. | Malvasia nera (from Costa V.) | yes | 32/32 | 2,23E+15 | | | | | | | |
| Lambrusca di Alessandria N. | Neretto di Marengo | yes | 32/32 | 2,11E+15 | | 13 | 0.24 | 0.02 | 0.98 | 0 | 0.25 |
| Lambrusca di Alessandria N. | Primaticcia | yes | 32/32 | 2,47E+15 | | | | | | | |
| Lambrusca di Alessandria N. | Unknown black (from Spineto) | | 32/32 | 1,97E+15 | | 16 | 0.31 | 0 | 1 | 0 | 0.25 |
| Lambruschetta | Timorasso B. | | 32/32 | 2,15E+15 | | | | | | | |
| Luglienga bianca (S. Anna di Lipsia B.) | Luglienga moscata | yes | 32/32 | 2,24E+15 | | | | | | | |

| | | | | | | | | | | | |
|---|---|---|---|---|---|---|---|---|---|---|---|
| Luglienga bianca (S. Anna di Lipsia B.) | Prié blanc B. | yes* | 32/32 | 2,24E+15 | | 11 | 0.22 | 0 | 1 | 0 | 0.25 |
| Luglienga moscata | Moscato bianco B. | yes | 32/32 | 2,02E+15 | | | | | | | |
| Maiolina N. | Montanera N. | | 32/32 | 2,05E+15 | | | | | | | |
| Malvasia aromatica di Parma (Malvasia Casalini B.) | Malvasia bianca (from Vignale) | yes | 32/32 | 1,51E+15 | | | | | | | |
| Malvasia aromatica di Parma (Malvasia Casalini B.) | Malvasia di Candia aromatica B. | | 32/32 | 2,24E+15 | | | | | | | |
| Malvasia aromatica di Parma (Malvasia Casalini B.) | Malvasia di Casorzo N. | yes | 32/32 | 1,13E+15 | | 9 | 0.17 | 0 | 1 | 0 | 0.25 |
| Malvasia aromatica di Parma (Malvasia Casalini B.) | Malvasia moscata B. | | 32/32 | 1,68E+15 | | 9 | 0.16 | 0.01 | 0.99 | 0 | 0.25 |
| Malvasia aromatica di Parma (Malvasia Casalini B.) | Malvasia nera (from Costa V.) | yes | 32/32 | 1,48E+15 | | | | | | | |
| Malvasia aromatica di Parma (Malvasia Casalini B.) | Malvasia nera a grappolo corto | | 32/32 | 9,88E+14 | | | | | | | |
| Malvasia aromatica di Parma (Malvasia Casalini B.) | Malvasia nera lunga N. | yes | 32/32 | 1,79E+15 | | 7 | 0.13 | 0.01 | 0.94 | 0.05 | 0.26 |
| Malvasia aromatica di Parma (Malvasia Casalini B.) | Moscato bianco B. | | 32/32 | 2,05E+15 | | 12 | 0.22 | 0.02 | 0.98 | 0 | 0.25 |
| Malvasia aromatica di Parma (Malvasia Casalini B.) | Nebùe | yes | 32/32 | 1,53E+15 | | | | | | | |
| Malvasia aromatica di Parma (Malvasia Casalini B.) | Ruché N. | yes | 32/32 | 1,93E+15 | | 9 | 0.16 | 0.01 | 0.99 | 0 | 0.25 |
| Malvasia di Schierano N. | Moscato bianco B. | | 32/32 | 1,81E+15 | | 11 | 0.2 | 0.01 | 0.99 | 0 | 0.25 |
| Millegusti | Moscato bianco B. | | 32/32 | 1,48E+15 | | 16 | 0.3 | 0 | 1 | 0 | 0.25 |
| Moissan | Mossano (from Canavese area) | yes | 32/32 | 1,78E+15 | | | | | | | |
| Moissan | Neirera | | 32/32 | 1,72E+15 | | | | | | | |
| Moissan | Neretto di Salto | | 31/32 | 1,23E+15 | VvMD36 | 12 | 0.22 | 0.01 | 0.99 | 0 | 0.25 |
| Moissan | Unknown black (from Pomaretto) | yes | 32/32 | 1,65E+15 | | 11 | 0.21 | 0.01 | 0.98 | 0.01 | 0.25 |
| Moradella N. | Uva delle cascine | yes | 32/32 | 1,63E+15 | | | | | | | |
| Morio muskat | Moscato bianco B. | yes* | 32/32 | 1,92E+15 | | | | | | | |
| Moscato bianco B. | Moscato bianco precoce | yes | 32/32 | 1,85E+15 | | 7 | 0.12 | 0.01 | 0.96 | 0.03 | 0.26 |
| Moscato bianco B. | Moscato di Scanzo N. | | 32/32 | 1,59E+15 | | 16 | 0.29 | 0 | 1 | 0 | 0.25 |
| Moscato bianco B. | Moscato giallo B. | | 32/32 | 1,36E+15 | | 15 | 0.27 | 0.02 | 0.97 | 0.01 | 0.25 |
| Moscato bianco B. | Moscato nero (from Borbera valley) | yes | 32/32 | 1,32E+15 | | | | | | | |
| Moscato bianco B. | Moscato nero di Acqui N. | yes | 32/32 | 1,93E+15 | | 12 | 0.22 | 0.01 | 0.95 | 0.04 | 0.26 |
| Moscato bianco B. | Moscato rosa Rs. | | 32/32 | 1,85E+15 | | 18 | 0.31 | 0.02 | 0.95 | 0.03 | 0.25 |
| Moscato bianco B. | Moschato mavro | | 32/32 | 1,79E+15 | | | | | | | |
| Moscato bianco B. | Muscat rouge de Madére | yes | 32/32 | 1,76E+15 | | | | | | | |
| Moscato bianco B. | Orange muscat (M. fleur d'oranger) | yes | 32/32 | 1,94E+15 | | | | | | | |
| Moscato bianco B. | Zibibbo B. (Muscat of Alexandria) | yes | 32/32 | 1,67E+15 | | 17 | 0.29 | 0.02 | 0.89 | 0.09 | 0.27 |
| **Moscato d'Amburgo N. (Muscat of Hamburg)** | Zibibbo B. (Muscat of Alexandria) | yes* | 32/32 | 2,36E+15 | | 25 | 0.44 | 0.03 | 0.91 | 0.06 | 0.26 |
| Muscat rouge de Madére | **Sciaccarello** | yes | 32/32 | 1,56E+15 | | | | | | | |
| Nebbiolo (false) 217 | Nebbiolo N. | | 32/32 | 1,39E+15 | | 8 | 0.16 | 0.01 | 0.99 | 0 | 0.25 |
| Nebbiolo gabardin | Neretta cuneese N. | yes | 32/32 | 1,79E+15 | | | | | | | |
| Nebbiolo N. | Negrera (from Valtellina) | | 32/32 | 2,06E+15 | | 6 | 0.12 | 0 | 0.92 | 0.08 | 0.27 |
| Nebbiolo N. | Neretto di Bairo N. | | 32/32 | 1,80E+15 | | 6 | 0.12 | 0.01 | 0.96 | 0.03 | 0.25 |
| Nebbiolo N. | Vespolina N. | yes | 31/32 | 1,19E+15 | VvMD36 (null allele) | 8 | 0.16 | 0 | 1 | 0 | 0.25 |
| Nebùe | Neretto di Marengo | yes | 32/32 | 2,01E+15 | | | | | | | |
| Negrello | Rachina | | 32/32 | 2,45E+15 | | | | | | | |
| Neretto di Marengo | Slarina N. | | 32/32 | 2,07E+15 | | 7 | 0.13 | 0.01 | 0.91 | 0.08 | 0.27 |
| Neretto di Marengo | Uvalino N. | | 32/32 | 2,60E+15 | | 12 | 0.22 | 0.02 | 0.98 | 0 | 0.25 |
| Neretto duro (Balau) | Teinturier ad acino rotondo | yes | 31/32 | 1,19E+15 | VrZAG79 | 7 | 0.12 | 0.01 | 0.91 | 0.08 | 0.27 |
| Orange muscat (M. fleur d'oranger) | **Perla di Csaba B. (Csaba gyoengye)** | yes* | 32/32 | 1,33E+15 | | | | | | | |
| Petit rouge N. | Vien de Nus N. | | 32/32 | 1,95E+15 | | 7 | 0.13 | 0.01 | 0.93 | 0.06 | 0.26 |
| **Pinot nero N. (Pinot noir)** | **Savagnin blanc** | | 32/32 | 2,23E+15 | | | | | | | |
| Rapallino | Spergola B. (Vernaccia di Oristano B.) | | 32/32 | 1,66E+15 | | 8 | 0.16 | 0.01 | 0.99 | 0 | 0.25 |
| Rossola nera N. | Rossola verde | | 32/32 | 1,54E+15 | | 8 | 0.16 | 0.01 | 0.99 | 0 | 0.25 |
| Tadone 1 (from Saluzzo) | Tadone 2 (from Saluzzo) | yes | 32/32 | 1,99E+15 | | 10 | 0.18 | 0.01 | 0.99 | 0 | 0.25 |
| Teinturier ad acino rotondo | Teinturier du Cher | yes | 32/32 | 2,21E+15 | | 10 | 0.17 | 0.02 | 0.97 | 0.02 | 0.25 |

Table S6 - List of cultivar pairs resulted linked by putative full-sibling (FS) relation and related statistics supported by likelihood of relation (nSSR) and by Cotterman's IBD coefficients (SNP). Putative half-siblings (HS) are reported only for unresolved FS or HS relations.

| | | SSR | SNPs | | | | |
|---|---|---|---|---|---|---|---|
| **Cultivar 1** | **Cultivar 2** | **Colony probability for FS pair relation** | **k0** | **k1** | **k2** | **kinship** | **Putative relation** |
| Aleatico (false, from Paderna) | Brachetto (false, from Nizza M.to) | 0.655 | Aleatico (false, from Paderna) not analyzed | | | | FS |
| Aleatico (false, from Paderna) | Millegusti | 0.681 | Aleatico (false, from Paderna) not analyzed | | | | FS |
| Arneis B. | Bottagera (false) | 0.770 | 0.22 | 0.57 | 0.21 | 0.25 | FS/HS |
| Biestro (presumed) | Dolcetto N. | not availaible | Biestro (presumed) not analyzed | | | | FS |
| Bonardina | Ruché N. | 1.000 | 0.22 | 0.52 | 0.26 | 0.26 | FS |
| Bordò | Malvasia aromatica di Parma (Malvasia Casalini B.) | 0.691 | 0.15 | 0.44 | 0.41 | 0.32 | FS |
| Brachetto (false, from Nizza M.to) | Millegusti | 0.658 | 0.16 | 0.53 | 0.32 | 0.29 | FS |
| Brachetto Migliardi | Malvasia di Casorzo N. | 1.000 | 0.33 | 0.47 | 0.20 | 0.22 | FS |
| Brachetto Migliardi | Malvasia nera (from Costa V.) | 1.000 | Malvasia nera (from Costa V.) not analyzed | | | | FS |
| Calora (presumed) | Gamba rossa N. (Gamba di pernice) | 0.464 | Calora (presumed) not analyzed | | | | HS |
| Calora (presumed) | Calora bianca (presumed) | 0.910 | Calora (presumed) not analyzed | | | | FS |
| Chardonnay B. | Gamay N. | 1.000 | 0.14 | 0.54 | 0.32 | 0.30 | FS |
| Fumin N. | Neyret N. | 0.990 | 0.32 | 0.63 | 0.05 | 0.18 | HS |
| Fumin N. | Petit rouge N. | 0.948 | 0.57 | 0.36 | 0.07 | 0.13 | HS |
| Gamba rossa N. (Gamba di pernice) | Calora bianca (presumed) | 0.841 | 0.33 | 0.58 | 0.09 | 0.19 | HS |
| Lambrusca pignata | Nebbiolo di Dronero (false) | 1.000 | Lambrusca pignata not analyzed | | | | FS |
| Malvasia di Casorzo N. | Malvasia nera (from Costa V.) | 1.000 | Malvasia nera (from Costa V.) not analyzed | | | | FS |
| Marzemino N. | Refosco dal peduncolo rosso N. | 0.771 | Refosco dal peduncolo rosso N. not analyzed | | | | FS |
| Moscato nero (from Borbera valley) | Moscato nero di Acqui N. | 0.488 | Moscato nero (from Borbera valley) not analyzed | | | | FS |
| Nebbiolo N. | Nebbiolo rosé | 0.948 | 0.08 | 0.45 | 0.47 | 0.35 | FS |
| Nebbiolo N. | Pignola N. | 0.948 | 0.24 | 0.46 | 0.30 | 0.26 | FS |
| Nebbiolo N. | Rossola nera N. | 1.000 | 0.26 | 0.40 | 0.35 | 0.27 | FS |
| Nebbiolo rosé | Pignola N. | 1.000 | 0.11 | 0.55 | 0.33 | 0.30 | FS |
| Nebbiolo rosé | Rossola nera N. | 0.948 | 0.19 | 0.54 | 0.26 | 0.27 | FS |
| Neyret N. | Petit rouge N. | 0.994 | 0.20 | 0.62 | 0.18 | 0.25 | FS/HS |
| Pignola N. | Rossola nera N. | 0.948 | 0.19 | 0.51 | 0.30 | 0.28 | FS |
| Sgavetta N. | Unknown black (from Tortona) | 1.000 | Both not analyzed | | | | FS |
| Syrah N. | Viognier B. | 0.073 | 0.38 | 0.49 | 0.13 | 0.19 | HS |
| Unknown black (from Sale) | Uva rara N. | 0.575 | 0.35 | 0.52 | 0.14 | 0.20 | FS/HS |

Table S7 - Most probable allele pairs (first row) and probability (second row) of the 7 reconstructed genotypes (by Colony) at 32 nSSR loci.

| Cultivar name | Average probability | VvMD5 | VvMD7 | VvMD25 | VvMD27 | VvMD28 | VvMD32 | VvS2 | VrZAG62 | VrZAG79 | VvMD36 | VrZAG67 | VrZAG64 | VvMD31 | VvMD24 | VRZAG21 | VvMD6 |
|---|---|---|---|---|---|---|---|---|---|---|---|---|---|---|---|---|---|
| Reconstructed genotype #1 | 0.719 | 239 239<br>0.485 | 233 253<br>0.504 | 257 257<br>0.870 | 184 184<br>0.812 | 262 262<br>0.413 | 253 275<br>0.378 | 135 143<br>0.633 | 192 204<br>0.878 | 250 250<br>0.733 | 249 265<br>0.965 | 132 150<br>0.949 | 137 139<br>0.982 | 209 211<br>0.407 | 213 213<br>0.481 | 191 203<br>0.962 | 190 208<br>0.999 |
| Reconstructed genotype #2 | 0.963 | 225 235<br>0.999 | 247 247<br>0.998 | 241 257<br>1 | 184 188<br>1 | 260 260<br>0.989 | 241 273<br>1 | 135 155<br>1 | 194 196<br>1 | 242 250<br>1 | 253 253<br>0.691 | 126 139<br>1 | 141 159<br>1 | 211 215<br>1 | 209 217<br>1 | 191 207<br>1 | 208 210<br>1 |
| Reconstructed genotype #3 | 0.916 | 231 233<br>1 | 247 247<br>0.921 | 251 257<br>1 | 178 178<br>0.927 | 236 238<br>0.988 | 241 251<br>0.994 | 139 139<br>0.711 | 204 204<br>0.849 | 248 258<br>1 | 263 271<br>0.766 | 153 153<br>0.780 | 137 137<br>0.857 | 203 215<br>0.998 | 213 217<br>0.984 | 203 207<br>0.995 | 208 210<br>0.997 |
| Reconstructed genotype #4 | 0.716 | 227 227<br>0.552 | 239 239<br>0.423 | 241 241<br>0.588 | 178 184<br>0.968 | 220 230<br>0.997 | 241 241<br>0.601 | 133 137<br>0.364 | 194 194<br>0.810 | 250 254<br>0.490 | 253 253<br>0.950 | 153 153<br>0.791 | 163 163<br>0.640 | 215 215<br>0.650 | 215 217<br>0.525 | 195 201<br>0.671 | 200 200<br>0.429 |
| Reconstructed genotype #5 | 0.673 | 225 225<br>0.563 | 239 249<br>0.521 | 257 257<br>0.676 | 188 188<br>0.708 | 220 236<br>0.407 | 241 251<br>0.996 | 133 135<br>0.875 | 194 194<br>0.710 | 250 250<br>0.748 | 253 253<br>1 | 153 153<br>0.663 | 163 163<br>0.744 | 211 215<br>0.500 | 213 217<br>0.986 | 191 201<br>0.979 | 200 208<br>0.365 |
| Reconstructed genotype #6 | 0.939 | 225 231<br>1 | 247 263<br>1 | 243 243<br>0.838 | 180 184<br>1 | 236 238<br>0.800 | 253 263<br>1 | 133 155<br>1 | 194 194<br>0.813 | 242 258<br>1 | 253 263<br>1 | 150 163<br>1 | 143 149<br>1 | 211 211<br>0.852 | 209 209<br>0.983 | 191 191<br>0.945 | 208 210<br>0.990 |
| Reconstructed genotype #7 | 0.838 | 231 235<br>1 | 249 253<br>0.999 | 241 259<br>0.861 | 188 188<br>0.939 | 236 270<br>0.686 | 241 257<br>0.999 | 155 155<br>0.653 | 194 200<br>0.828 | 250 250<br>0.893 | 263 294<br>0.942 | 126 153<br>0.988 | 141 163<br>0.987 | 203 211<br>0.701 | 213 213<br>0.830 | 191 207<br>1 | 200 208<br>0.898 |

| Cultivar name | VVMD21 | VMC5A1 | VMC2H4 | VMC1C10 | VVIB01 | VMC5G8 | VMC1E8 | VMC4C6 | VVMD34 | VMC3D12 | VvMD26 | VMC2F10 | VMC7F2 | VVIQ52 | VvIN73 | VVP31 |
|---|---|---|---|---|---|---|---|---|---|---|---|---|---|---|---|---|
| Reconstructed genotype #1 | 248 248<br>0.737 | 170 170<br>0.531 | 217 217<br>0.860 | 153 163<br>0.999 | 291 291<br>0.664 | 302 316<br>0.760 | 206 208<br>0.536 | 165 165<br>0.738 | 237 237<br>0.775 | 200 202<br>0.461 | 247 261<br>0.870 | 90 100<br>0.873 | 201 205<br>0.642 | 81 85<br>0.652 | 263 263<br>1 | 174 174<br>0.466 |
| Reconstructed genotype #2 | 248 248<br>0.839 | 166 170<br>0.999 | 201 217<br>0.999 | 153 171<br>1 | 291 299<br>1 | 316 316<br>0.989 | 218 228<br>1 | 159 159<br>0.750 | 237 239<br>1 | 206 206<br>0.967 | 247 247<br>0.996 | 94 104<br>1 | 201 201<br>0.999 | 85 85<br>0.618 | 263 263<br>0.990 | 180 192<br>1 |
| Reconstructed genotype #3 | 248 254<br>1 | 160 172<br>1 | 217 217<br>0.906 | 153 165<br>1 | 291 291<br>0.663 | 302 312<br>1 | 230 232<br>1 | 167 169<br>0.895 | 237 237<br>0.992 | 222 222<br>0.583 | 247 249<br>1 | 104 108<br>1 | 201 203<br>1 | 79 81<br>0.524 | 261 263<br>0.997 | 180 194<br>1 |
| Reconstructed genotype #4 | 242 248<br>0.695 | 166 170<br>0.689 | 203 229<br>0.709 | 143 153<br>0.744 | 289 299<br>0.909 | 316 316<br>0.467 | 226 230<br>0.397 | 165 177<br>0.946 | 237 237<br>0.951 | 206 222<br>0.942 | 249 253<br>0.570 | 94 116<br>0.996 | 201 201<br>0.844 | 81 85<br>0.956 | 263 265<br>0.664 | 180 182<br>0.983 |
| Reconstructed genotype #5 | 248 248<br>0.769 | 170 170<br>0.486 | 201 217<br>0.473 | 159 181<br>0.531 | 291 295<br>0.830 | 302 302<br>0.518 | 208 232<br>0.495 | 169 169<br>0.442 | 237 237<br>0.951 | 222 222<br>0.573 | 253 253<br>0.507 | 94 104<br>0.517 | 201 203<br>0.602 | 79 79<br>0.775 | 261 263<br>0.936 | 184 184<br>0.681 |
| Reconstructed genotype #6 | 242 248<br>1 | 166 170<br>0.661 | 203 217<br>0.985 | 171 181<br>1 | 295 299<br>0.943 | 302 316<br>0.999 | 218 230<br>0.961 | 159 159<br>0.957 | 237 239<br>0.998 | 206 212<br>0.999 | 247 247<br>0.974 | 94 104<br>0.569 | 201 201<br>0.964 | 79 79<br>0.821 | 263 263<br>0.989 | 176 196<br>1 |
| Reconstructed genotype #7 | 248 248<br>0.908 | 166 166<br>0.600 | 217 217<br>0.877 | 153 153<br>0.956 | 291 291<br>0.793 | 316 316<br>0.636 | 222 230<br>0.555 | 159 165<br>0.996 | 237 237<br>0.915 | 198 206<br>1 | 249 249<br>0.909 | 94 94<br>0.588 | 201 201<br>0.893 | 79 85<br>0.496 | 261 263<br>0.537 | 180 180<br>0.943 |

Table S8 - List of the nuclear and chloroplastic SSR markers used in this study.

| SSR marker | Reference | Type | Linkage group |
|---|---|---|---|
| VMC1C10 | Vitis Microsatellite Consortium (Agrogene SA) | Nuclear | IX |
| VMC1E8 | Vitis Microsatellite Consortium (Agrogene SA) | Nuclear | VIII |
| VMC2F10 | Vitis Microsatellite Consortium (Agrogene SA) | Nuclear | VI |
| VMC2H4 | Vitis Microsatellite Consortium (Agrogene SA) | Nuclear | XII |
| VMC3D12 | Vitis Microsatellite Consortium (Agrogene SA) | Nuclear | XIII |
| VMC4C6 | Vitis Microsatellite Consortium (Agrogene SA) | Nuclear | V |
| VMC5A1 | Vitis Microsatellite Consortium (Agrogene SA) | Nuclear | XVI |
| VMC5G8 | Vitis Microsatellite Consortium (Agrogene SA) | Nuclear | XV |
| VMC7F2 | Vitis Microsatellite Consortium (Agrogene SA) | Nuclear | XVIII |
| VRZAG21 | [84] | Nuclear | IV |
| VrZAG62 | [84] | Nuclear | VII |
| VrZAG64 | [84] | Nuclear | X |
| VrZAG67 | [84] | Nuclear | X |
| VrZAG79 | [84] | Nuclear | V |
| VVIB01 | [85] | Nuclear | II |
| VvIN73 | [85] | Nuclear | XVII |
| VVIQ52 | [85] | Nuclear | IX |
| VVMD21 | [86] | Nuclear | VI |
| VvMD24 | [86] | Nuclear | XIV |
| VvMD25 | [86] | Nuclear | XI |
| VvMD26 | [86] | Nuclear | I |
| VvMD27 | [86] | Nuclear | V |
| VvMD28 | [86] | Nuclear | III |
| VvMD31 | [86] | Nuclear | VII |
| VvMD32 | [86] | Nuclear | IV |
| VVMD34 | [87] | Nuclear | II |
| VvMD36 | [87] | Nuclear | III |
| VvMD5 | [86] | Nuclear | XVI |
| VvMD6 | [86] | Nuclear | VII |
| VvMD7 | [86] | Nuclear | VII |
| VVP31 | [85] | Nuclear | XIX |
| VvS2 | [88] | Nuclear | XI |
| ccmp3 | [89] | Chloroplastic | - |
| ccmp5 | [89] | Chloroplastic | - |
| ccmp10 | [89] | Chloroplastic | - |
| ccSSR9 | [34] | Chloroplastic | - |

Figure S1 - The complete kinship network of the traditional grape varieties from north western Italy and their close relatives. White and coloured grape cultivars are framed in green and purple lines respectively. Black connector lines indicate parent/offspring relationships; the arrows point to the offspring. Red connectors and red arrows refer to reconstructed genotypes (not included into the sample set).

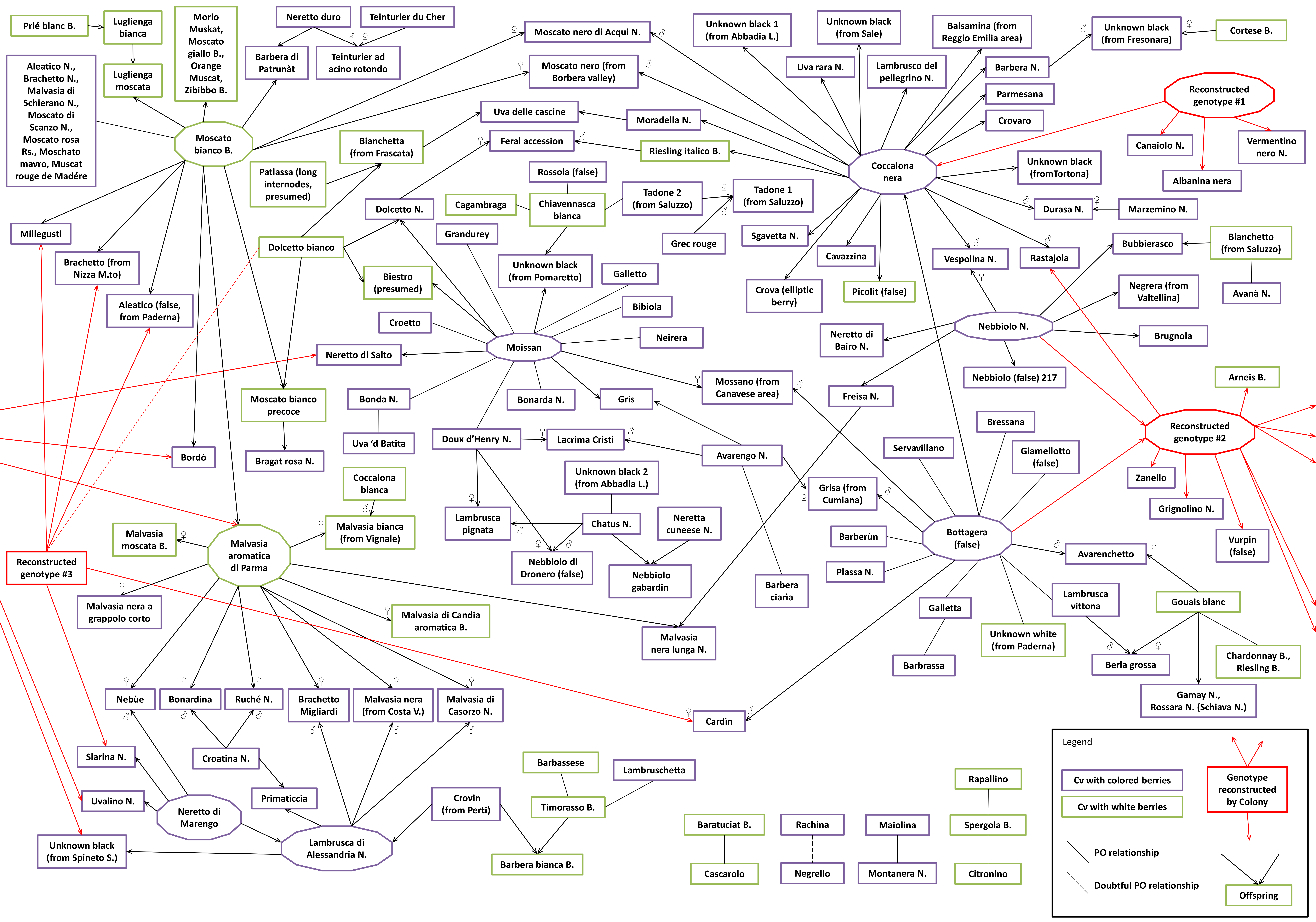

Supplement: Supplementary file 2 — Supplementary file2 [file 41598_2020_72799_MOESM2_ESM.pdf]
